# Predicting miRNA-Disease Association Based on Modularity Preserving Heterogeneous Network Embedding

**DOI:** 10.3389/fcell.2021.603758

**Published:** 2021-06-10

**Authors:** Wei Peng, Jielin Du, Wei Dai, Wei Lan

**Affiliations:** ^1^Faculty of Information Engineering and Automation, Kunming University of Science and Technology, Kunming, China; ^2^Computer Technology Application Key Laboratory of Yunnan Province, Kunming University of Science and Technology, Kunming, China; ^3^Guangxi Key Laboratory of Multimedia Communications and Network Technology, Guangxi University, Nanning, China

**Keywords:** heterogeneous network embedding, matrix factorization, miRNA, disease, miRNA-disease association prediction

## Abstract

MicroRNAs (miRNAs) are a category of small non-coding RNAs that profoundly impact various biological processes related to human disease. Inferring the potential miRNA-disease associations benefits the study of human diseases, such as disease prevention, disease diagnosis, and drug development. In this work, we propose a novel heterogeneous network embedding-based method called MDN-NMTF (Module-based Dynamic Neighborhood Non-negative Matrix Tri-Factorization) for predicting miRNA-disease associations. MDN-NMTF constructs a heterogeneous network of disease similarity network, miRNA similarity network and a known miRNA-disease association network. After that, it learns the latent vector representation for miRNAs and diseases in the heterogeneous network. Finally, the association probability is computed by the product of the latent miRNA and disease vectors. MDN-NMTF not only successfully integrates diverse biological information of miRNAs and diseases to predict miRNA-disease associations, but also considers the module properties of miRNAs and diseases in the course of learning vector representation, which can maximally preserve the heterogeneous network structural information and the network properties. At the same time, we also extend MDN-NMTF to a new version (called MDN-NMTF2) by using modular information to improve the miRNA-disease association prediction ability. Our methods and the other four existing methods are applied to predict miRNA-disease associations in four databases. The prediction results show that our methods can improve the miRNA-disease association prediction to a high level compared with the four existing methods.

## Introduction

MicroRNA (miRNA) is a category of small endogenous single-stranded non-coding RNA molecules with about 22 nucleotides in length. They play an essential role in regulating gene expression and complex gene regulatory networks by repressing target mRNAs expression at the post-transcriptional level (Bartel, 2004; Meister and Tuschl, 2004). Studies show that about 60% of human protein-coding genes are targeted by miRNAs, where the 5′ region of miRNA binds to 3′ UTR of the target mRNAs (Friedman et al., 2009). With the rapid development of biotechnology, increasing research has demonstrated that miRNAs play crucial roles at multiple stages of many critical biological processes such as early cell growth, development, proliferation, differentiation, tumor invasion, and apoptosis (Ambros, 2003). Furthermore, studies have shown that abnormality and dysregulations of disease-related miRNAs may cause human diseases (Garzon et al., 2010). Therefore, inferring the potential miRNA-disease association is of great benefit to studying human diseases, such as disease prevention, disease diagnosis, and drug development. As we all know, discovering the miRNA-disease associations through traditional biological experiments is a time-consuming and labor-intensive process. Instead, computational models would serve as a low-cost, and high-efficiency way of predicting miRNA-disease associations.

Previous researches observe that similar miRNAs tend to associate with the same diseases and similar diseases are highly likely related to the same miRNAs. Hence, many computational methods construct disease similarity network and miRNA similarity network and infer miRNA-disease associations based on the associations between or within the disease or miRNAs (Peng et al., 2016, 2017; Zou et al., 2016; Huang et al., 2019). Xuan et al. (2013) construct a miRNA similarity network according to the degree of two miRNAs sharing similar disease and consider the *k* most similar neighbors of each miRNA to infer miRNA-disease associations. Chen X. et al. (2012) implement a random walk on the miRNA functional similarity network and explore the potential miRNA-disease associations from the global network information. Xuan et al. (2015) divide the miRNA nodes in the miRNA similarity network into two categories: the given disease-related and the given disease-unrelated nodes. They assign different transition weights to different types of nodes and implement random walk on the miRNA similarity network to predict miRNA-disease associations. Besides the single network, some researchers build a heterogeneous network that consists of miRNAs, diseases, and their inter and intro associations. Liu et al. (2017) construct the miRNA similarity network, disease similarity network and known miRNA-disease association network. After that, they run a random walk on the heterogeneous network to propagate information and exploit potential miRNA-disease associations. Considering the difference in the network structure of the miRNA similarity network and disease similarity network, Luo and Xiao (2017) use an unbalanced Bi-Random walk (called UBiRW) on the heterogeneous network of disease similarity network, miRNA functional similarity network and a known miRNA-disease association network to infer potential miRNA-disease associations. Zeng et al. (2016) enumerate all of the paths from miRNA/disease to disease/miRNA in the heterogeneous network, and the final score between a miRNA and a disease is a linear combination of their path scores. You et al. (2017) construct the heterogeneous network by integrating known human miRNA-disease associations, miRNA functional similarity, disease semantic similarity, and the Gaussian Interaction Profile (GIP) kernel similarity. After that, they do a depth-first search to find the paths between the miRNAs and diseases on the heterogeneous network. Then they filter the long paths and calculate the association of miRNA and disease by combining all their paths.

Recently, a group of researchers proposes the network embedding-based method to predict miRNA-disease associations. The network embedding method designs an objective function and converts the network nodes into a low dimensional vector while maximally preserves the network structural information. Chen and Yan (2014) develop a regularized least square method to learn the latent vectors for miRNAs and diseases on the miRNA similarity network and disease similarity network. They combine the two vectors to give the final solution of predicting new miRNA-disease associations. Lan et al. (2016) construct multi-kernels to store the miRNA functional similarity network, miRNA sequence similarity network and disease semantic similarity network. Then they employ a Bayesian matrix factorization method to infer potential miRNA-disease associations by integrating these data sources. Yan et al. (2019) develop a dynamic neighborhood regularized logistic matrix factorization method called DNRLMF-MDA to learn representation vectors for miRNAs and diseases and predict potential miRNA-disease associations. Li et al. (2017) design an objective function to ensure the scores of known miRNA-disease association matrix are close to those in the predicted miRNA-disease association matrix. They utilize the matrix completion algorithm to update the matrix of known miRNA-disease associations and to predict the potential associations. Xiao et al. (2018) use a graph regularized non-negative matrix factorization framework (named GRNMF) to identify possible associations for all diseases simultaneously. Similarly, Chen’s group proposes two matrix completion-based methods, namely IMCMDA (Chen et al., 2018), and NCMCMDA (Chen et al., 2020) for miRNA-disease association prediction. The differences are IMCMDA uses inductive matrix completion for miRNA-disease association prediction, while NCMCMDA integrates neighborhood constraint in the course of matrix completion.

The methods mentioned above have achieved great success in predicting miRNA-disease associations. However, there are still some shortcomings in these existing methods. Firstly, the single network-based methods only use the miRNA similarity or disease similarity network. They may ignore the relationship between diseases or miRNAs. Secondly, seldom heterogeneous network-based methods consider the miRNA/disease similarity network’s modular structure. Although some network embedding-based methods, i.e., DNRLMF-MDA, NCMCMDA, learn node representation only considering the constraint from part of neighbors, most of them ignore the modular information of miRNAs and diseases. Lu et al. (2008) constructed disease network by giving two diseases an edge if they share at least one common associated miRNA. Diseases cluster together, which suggests that some diseases form modules sharing similar associations at the miRNA level. Moreover, the disease-associated miRNAs show various dysfunctions, such as mutation, upregulation, deleted, and downregulation. On the other hand, groups of homologous miRNA belong to the same miRNA families. They might have similar functions, and therefore, their dysfunction would lead to a similar phenotype. By analyzing members in disease modules or miRNA modules, researchers found that most of the members in the miRNA module are related to the same disease, and the members in the disease modules are mostly related to the same miRNA too. Therefore, this finding can guide us to predict novel disease-related miRNAs.

In this work, we propose a novel heterogeneous network embedding-based method for predicting miRNA-disease associations. We calculate the disease semantic similarity, diseases functional similarity, miRNA functional similarity, and compute the GIP kernel similarity of miRNAs and diseases. Then, we integrate these similarities and construct a heterogeneous network of the disease similarity network, miRNA functional similarity network and a known miRNA-disease association network. After that, we propose a Module-based Dynamic Neighborhood Non-negative Matrix Tri-Factorization (MDN-NMTF) to learn the latent vector representation for miRNAs and diseases in the heterogeneous network. Finally, the association probability is computed by the product of the latent miRNA and disease vectors. MDN-NMTF not only successfully integrates diverse biological information of miRNAs and diseases to predict miRNA-disease associations, but also considers the module properties of miRNAs and diseases in the course of learning vector representation, which can maximally preserve the heterogeneous network structural information and the network properties. Meanwhile, we also extend MDN-NMTF to a new version (called MDN-NMTF2) by using the modular information to improve the prediction ability of MDN-NMTF. Our methods, as well as the other four existing methods [DNRLMF-MDA (Yan et al., 2019), IMCMDA (Chen et al., 2018), UBiRW (Luo and Xiao, 2017), and GRNMF (Xiao et al., 2018)], are applied to predict miRNA-disease associations on four data sets. The prediction results show that compared with the four existing methods, our methods can improve the performance of miRNA-disease association prediction to a high level.

## Materials

Four datasets (see Table 1), namely HMDD2.0-You (Li et al., 2014), HMDD2.0-Lan (Lan et al., 2016), HMDD2.0-Yan, and HMDD3.0^1^, were used to evaluate our methods and the other existing methods. HMDD2.0-You, HMDD2.0-Lan, and HMDD2.0-Yan were from HMDD database version 2.0. The HMDD2.0-You dataset includes 495 miRNAs, 380 diseases and 5,424 miRNA-disease associations. The HMDD2.0-Lan dataset consists of 550 miRNAs, 329 diseases and 6,084 miRNA-disease associations. The HMDD2.0-Yan dataset includes 576 miRNAs, 356 diseases, and 6,391 miRNA-disease associations. The HMDD3.0 dataset came from HMDD database version 3.0, which involves 1,207 miRNAs, 894 diseases, and 18,732 miRNA-disease associations. To calculate the functional similarity of diseases, we extracted the functional similarity scores of gene-gene pairs from the HumanNet database that contains 16,243 genes and 476,399 associations (Lee et al., 2011). The disease-gene associations of HMDD2.0-You, HMDD2.0-Yan, and HMDD3.0 were obtained from the DisGeNET database, where includes 13,000 diseases, over 16,000 genes, and 380,000 disease-gene associations (Piñero et al., 2015). The disease-gene associations of HMDD2.0-Lan were downloaded from the SIDD database, containing 2,603 genes, 2,817 diseases, and 117,190 disease-gene associations (Cheng et al., 2013).

**TABLE 1:** The number of MiRNAs, diseases and miRNA-disease associations in four datasets.

**Dataset**	***n*_*m*_**	***n*_*d*_**	***n*_*md*_**
HMDD2.0-You	495	380	5424
HMDD2.0-Lan	550	329	6084
HMDD2.0-Yan	576	356	6391
HMDD3.0	1207	894	18732

*The table shows the differences among the four datasets, where *n*_*m*_, *n*_*d*_, and *n*_*md*_ indicate the number of miRNAs, diseases and miRNA-disease associations, respectively.*

## Methods

**FIGURE 1:**
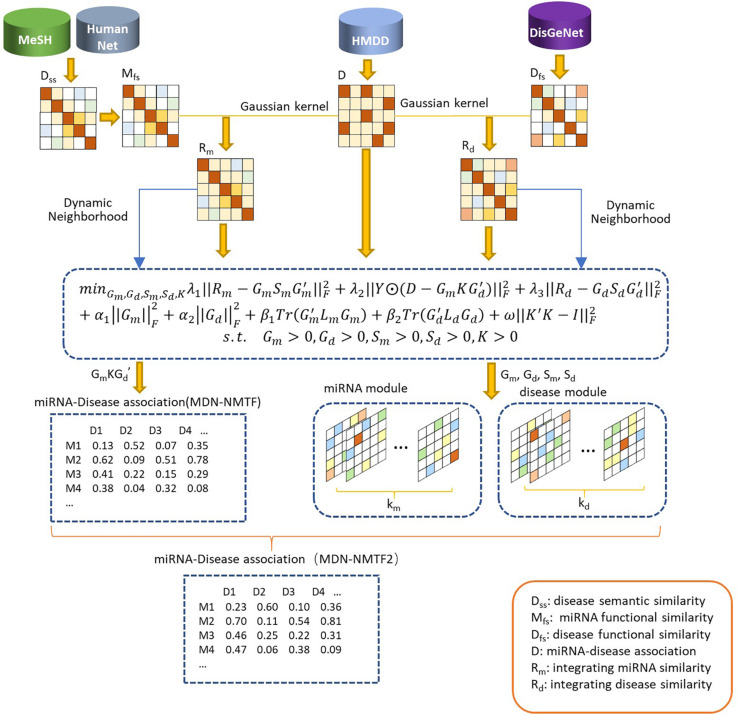
The flowchart of MDN-NMTF and MDN-NMTF2 to predict miRNA-disease association. The MDN-NMTF model takes four steps to predict miRNA-disease associations: building the similarity networks, learning feature representation for miRNA and diseases; reconstructing the miRNA-disease association network, predicting miRNA-disease associations. MDN-NMTF2 is an extended version of MDN-NMTF. It divides the miRNAs and diseases into several modules on the basis of the representation vectors learned by MDN-NMTF. MDN-NMTF2 calculates the similarity of two miRNAs or two diseases based on the module they belong to and infers the novel miRNA-disease associations from similar miRNAs or diseases in the same modules.

The MDN-NMTF model aims to learn the representation vectors for miRNAs and diseases and to achieve better prediction for disease-related miRNAs. It can maximally maintain their features in original spaces, i.e., known miRNA-disease associations, miRNA similarity network structure, and disease similarity network structure. The preparing process of MDN-NMTF is broadly divided into four steps: building the networks, learning feature representation; reconstructing the miRNA-disease association network, predicting miRNA-disease associations (see Figure 1).

### Disease Semantic Similarity

The disease semantic similarity between diseases is calculated using Mesh descriptors of diseases (Nelson et al., 2002). The disease terms can be represented as a direct acyclic graph (DAG), where nodes represent disease terms and edges represent the associations between diseases. The similarity between diseases can be calculated according to their common ancestors in the DAGs.

Let DAG*_*d*_* represent disease *d*, DAG*_*d*_* = (*T_*d*_, E_*d*_*). *T*_*d*_ is the set composed of all parent disease nodes of *d* and itself, and *E*_*d*_ is the set of all edges between disease nodes within *T*_*d*_. The formula to calculate the semantic value DV*_*d*_(t)* of diseases *t* and *d* is as follows:

(1) $$\begin{array}{c} {\text{DV}}_{d}\,\left(t\right)=\left\{ \begin{array}{c} 1,\mathrm{if}\,t=d\\ \max\left\{\Delta \times {\text{DV}}_{d}\,\left(t^{\prime }\right)|t^{\prime }\in \text{children}\,\text{of}\,t\right\},\text{if}\,t\neq d \end{array}\right. \end{array}$$

Where *t* is the set of all common ancestors of diseases *d*. Δ is the semantic contribution factor, whose value is between 0 and 1. We set the value of Δ to 0.5 in this study, similar to the values in (Yan et al., 2019). DS(*d*) is the semantic values of a disease *d* in DAG.

(2) $$\begin{array}{c} \text{DS}\,\left(d\right)=\sum_{t\in T_{d}}{\text{DV}}_{d}\,\left(t\right) \end{array}$$

The semantic similarity between disease *d*_*i*_ and disease *d*_*j*_ is as follows:

(3) $$\begin{array}{c} D_{s\,s}\,\left(d_{i},d_{j}\right)=\frac{\sum_{t\in T_{d_{i}}\,\cap T_{d_{j}}}\left(D\,V_{d_{i}}\,\left(t\right)+D\,V_{d_{j}}\,\left(t\right)\right)}{D\,S\,\left(d_{i}\right)+D\,S\,\left(d_{j}\right)} \end{array}$$

### Disease Functional Similarity

Calculating disease functional similarity is based on the assumption that similar diseases target similar disease genes (Cheng et al., 2014). Therefore, given a pair of diseases *d*_*a*_ and *d*_*b*_, the functional similarity is defined as:

(4) $$\begin{array}{c} D_{\text{fs}}\,\left(d_{a},d_{b}\right)=\frac{\sum_{1\leq i\leq m}{\text{GFS}}_{G_{b}}\,\left(g_{\text{ai}}\right)+\sum_{1\leq j\leq n}{\text{GFS}}_{G_{a}}\,\left(g_{\text{bj}}\right)}{m+n} \end{array}$$

Where *G*_*a*_ = {*g*_*a*1_,*g*_*a*2_,…,*g*_am_} and *G*_*b*_ = {*g*_*b*1_,*g*_*b*2_,…,*g*_bn_} are two gene sets which associate with diseases *d*_*a*_ and *d*_*b*_, respectively, and *m* and *n* are the numbers of genes in *G*_*a*_ and *G*_*b*_, respectively. GFS_*G*_*b*__(*g*_ai_) denotes the functional similarity between gene *g*_*ai*_ and the genes in *G*_*b*_. It can be defined as below:

(5) $$\begin{array}{c} {\text{GFS}}_{G_{b}}\,\left(g_{\text{ai}}\right)=\max_{1\leq j\leq n}\left(\text{FS}\,\left(g_{\text{ai}},g_{\text{bj}}\right)\right) \end{array}$$

Where FS(*g*_*ai*_, *g*_*bj*_) denotes the functional similarity between gene *g*_*ai*_ and gene *g*_*bj*_, which is obtained from HumanNet dataset (Lee et al., 2011) in this work. In the same way, the GFS_*G*_*a*__(*g*_bj_) can be computed.

### MiRNA Functional Similarity

The miRNA functional similarity between two miRNAs *m*_1_ and *m*_2_ is calculated based on the semantic similarity of diseases to which they are related. It can be defined as follows:

(6) $$\begin{array}{c} M_{\text{fs}}\,\left(m_{1},m_{2}\right)=\frac{\sum_{1\leq i\leq n_{1}}{\text{MFS}}_{{\text{DT}}_{2}}\,\left(d\,t_{1\,i}\right)+\sum_{1\leq j\leq n_{2}}{\text{MFS}}_{{\text{DT}}_{1}}\,\left(d\,t_{2\,j}\right)}{n_{1}+n_{2}} \end{array}$$

Where *n*_1_ and *n*_2_ are the number of diseases that are associated with miRNAs *m*_1_ and *m*_2_, respectively. DT_1_ and DT_2_ are the sets of diseases that are associated with miRNAs *m*_1_ and *m*_2_, respectively. MFS_*DT*_*_2_* (*dt*_1i_) is the semantic similarity of disease dt*_1i_* and the diseases in DT*_2_*, which is defined as below:

(7) $$\begin{array}{c} {\text{MFS}}_{{\text{DT}}_{2}}\,\left(d\,t_{1\,i}\right)=\max_{1\leq j\leq n_{2}}\left(D_{\text{ss}}\,\left(d\,t_{1\,i},d\,t_{2\,j}\right)\right) \end{array}$$

(8) $$\begin{array}{c} {\text{MFS}}_{{\text{DT}}_{1}}\,\left(d\,t_{2\,j}\right)=\max_{1\leq i\leq n_{1}}\left(D_{\text{ss}}\,\left(d\,t_{1\,i},d\,t_{2\,j}\right)\right) \end{array}$$

Where *D*_*ss*_ (*dt_1i_, dt_2j_*) is the semantic similarity between diseases *dt*_1i_ and *dt*_2j_.

### GIP Kernel Similarity

It is observed that miRNAs with similar functions are more likely to be associated with similar diseases and vice versa. According to this observation, GIP kernel similarity is constructed to describe the miRNA similarity and disease similarity (Laarhoven et al., 2011). First, we defined a binary vector *IP* (*m*_*i*_) to represent the interaction profile of miRNA *m*_*i*_ by observing whether or not there is a known association between miRNA *m*_*i*_ and every disease. Then, the GIP similarity between miRNA *m*_*i*_ and *m*_*j*_ can be calculated as:

(9) $$\begin{array}{c} {\text{KM}}_{\text{GIP}}\,\left(m_{i},m_{j}\right)=\exp\left(-\gamma_{m}\,{||\text{IP}\,\left(m_{i}\right)-\text{IP}\,\left(m_{j}\right)||}^{2}\right) \end{array}$$

Where, *γ_*m*_* controls the kernel bandwidth, which normalizes another bandwidth parameter *γ_*m*_′* by the average number of related miRNAs per disease. *γ_*m*_* is defined as follows:

(10) $$\begin{array}{c} \gamma_{m}=\gamma_{m}^{{}^{\prime }}/\left(\frac{1}{n_{m}}\,\sum_{i=1}^{n_{m}}{||\text{IP}\,\left(m_{i}\right)||}^{2}\right) \end{array}$$

Here $\gamma_{m}^{{}^{\prime }}$ is set to be 1 based on the previous study (Yan et al., 2019). *n*_*m*_ is the number of miRNAs.

Thus, the GIP kernel similarity between disease *d*_*i*_ and *d*_*j*_ is defined as follows:

(11) $$\begin{array}{c} {\text{KD}}_{\text{GIP}}\,\left(d_{i},d_{j}\right)=\text{exp}\,\left(-\gamma_{d}\,{||\text{IP}\,\left(d_{i}\right)-\text{IP}\,\left(d_{j}\right)||}^{2}\right) \end{array}$$

(12) γd=γd/′(1nd∑i=1nd||IP(di)||2)

Where *γd^′^* is also set to 1 and *n*_*d*_ is the number of diseases.

### Integrating Similarity for miRNAs and Diseases

Because not all miRNA-miRNA pairs have functional similarity, the GIP kernel similarity for miRNA is interpolated to the miRNA functional similarity to obtain the integrated similarity for miRNA. The final miRNA similarity matrix between miRNA *m*_*i*_ and miRNA *m*_*j*_ is defined as follows:

(13) $$\begin{array}{c} R_{m}\,\left(m_{i},m_{j}\right)=\left\{ \begin{array}{c} M_{f\,s}\,\left(m_{i},m_{j}\right),\text{if}\,M_{\text{fs}}\,\left(m_{i},m_{j}\right)>0\\ {\text{KM}}_{\text{GIP}}\,\left(m_{i},m_{j}\right),\text{otherwise} \end{array}\right. \end{array}$$

Similarly, the final disease similarity between disease *d*_*i*_ and disease *d*_*j*_ is defined as follows:

(14) $$\begin{array}{c} R_{d}\,\left(d_{i},d_{j}\right)=\left\{ \begin{array}{c} D_{\text{fs}}\,\left(d_{i},d_{j}\right),\mathrm{if}\,D_{f\,s}\,\left(d_{i},d_{j}\right)>0\\ {\text{KD}}_{\text{GIP}}\,\left(d_{i},d_{j}\right),\text{otherwise} \end{array}\right. \end{array}$$

### Regularized by Dynamic Neighborhood

Similar to previous DNRLMF-MDA method (Yan et al., 2019), we only preserve the relationships between a miRNA or a disease and their closest neighbors, when projecting the miRNA or disease to their latent spaces. Let *N*(*m*_*i*_) and *N*(*d*_*j*_) denote the set of nearest neighbors of miRNA *m*_*i*_ and disease *d*_*j*_, respectively. The numbers of nearest neighbors of the miRNAs are not fixed but are dynamically determined according to Eq. (15). For miRNA *m*_*i*_, *h*(*m*_*i*_) denotes its number of nearest neighbors, which can be as follows:

(15) h⁢(mi)={max⁡(H),if⁢1-rs⁢(mi)ll≤εl,1≤l≤H0,otherwise

Where ε is the control parameter. It is set to 0.56 via cross-validation. The rs(*m*_*i*_) is a ranked vector based on the similarity between miRNA *m*_*i*_ and other miRNAs from high to low, and rs(*m*_*i*_)*_*l*_* is the *l*th most similar value. *H* integer ranges from 1 to the total number of *m*_*i*_’s neighbors and *l* (the exponent of ε) is a dynamic variable integer to satisfy the constraint. Similarly, for disease *d*_*i*_, its number of nearest neighbors (*h*(*d*_*j*_)) also can be formulated as follows:

(16) h⁢(dj)={max⁡(H),if⁢1-rs⁢(dj)ll≤εl,1≤l≤H0,otherwise

Where rs(*d*_*j*_) is a ranked vector based on the similarities between disease *d*_*j*_ and other diseases from high to low, and rs(*d*_*j*_)*_*l*_* is the *l*th most similar value.

Let matrix *A* be the dynamic nearest neighborhood matrix of miRNAs, its element *a_*i*__μ_* is calculated as below:

(17) $$\begin{array}{c} a_{i\,\mu }=\left\{ \begin{array}{c} R_{m}\,\left(m_{i},m_{\mu }\right),\mathrm{if}\,m_{\mu }\in N\,\left(m_{i}\right)\\ 0,\text{otherwise} \end{array}\right. \end{array}$$

Similarity, let matrix *B* be the dynamic nearest neighborhood matrix of diseases, its element *b_*j*__ν_* can be calculated as below:

(18) $$\begin{array}{c} b_{j\,\upsilon }=\left\{ \begin{array}{c} R_{d}\,\left(d_{j},d_{\upsilon }\right),\text{if}\,d_{\upsilon }\in N\,\left(d_{j}\right)\\ 0,\text{otherwise} \end{array}\right. \end{array}$$

In this work, we assume that if the two miRNAs or two diseases are the nearest neighbors in their original similarity networks, they should show similar representations in the corresponding latent spaces. Hence, the following two regularization terms are designed for miRNA and disease, respectively, which will be incorporated into the MDN-NMTF objective function. The regularization term for miRNAs can be defined as the following equation (Liu et al., 2016):

(19) $$\begin{array}{c} \sum_{i=1}^{n_{m}}\sum_{\mu =1}^{n_{m}}a_{\text{iu}}\,{||{\text{gm}}_{i}-{\text{gm}}_{\mu }||}_{F}^{2}=\text{Tr}\,\left(G_{m}^{{}^{\prime }}\,L_{m}\,G_{m}\right) \end{array}$$

Where Tr() is the trace of a matrix, and Lm=(Dm+Dm′)-(A+A)′, in which *D*_*m*_ and $D_{m}^{{}^{\prime }}$ are the diagonal matrices, whose diagonal elements are $D_{\text{mii}}=\sum_{\mu =1}^{n_{m}}a_{i\,\mu }$ and $D_{m\,\mu \,\mu }^{{}^{\prime }}=\sum_{i=1}^{n_{m}}a_{i\,\mu }$, respectively. *G*_*m*_ represents the latent matrices of all miRNAs. Similarity, the regularization term for diseases can be defined as the following equation:

(20) $$\begin{array}{c} \sum_{j=1}^{n_{d}}\sum_{\upsilon =1}^{n_{d}}b_{j\,\upsilon }\,{||{\text{gd}}_{j}-{\text{gd}}_{\upsilon }||}_{F}^{2}=\text{Tr}\,\left(G_{d}^{{}^{\prime }}\,L_{d}\,G_{d}\right) \end{array}$$

Where Ld=(Dd+Dd′)-(B+B)′, in which *D*_*d*_ and $D_{d}^{{}^{\prime }}$ are the diagonal matrices, whose diagonal elements are $D_{\text{djj}}=\sum_{\upsilon =1}^{n_{d}}b_{j\,\upsilon }$ and $D_{d\,\upsilon \,\upsilon }^{{}^{\prime }}=\sum_{j=1}^{n_{d}}b_{j\,\upsilon }$, respectively. And *G*_*d*_ represents the latent matrices of all diseases.

### The MDN-NMTF Model

Let *R*_*m*_ ∈ *ℝ*^*n*_*m*_×*n*_*m*_^ and *R*_*d*_ ∈ *ℝ*^*n*_*d*_×*n*_*d*_^ denote the adjacency matrix of the miRNA similarity network and disease similarity network, respectively. The latent matrices of all miRNAs and diseases are represented as *G*_*m*_ ∈ *ℝ*^*n*_*m*_×*k*_*m*_^ and *G*_*d*_ ∈ *ℝ*^*n*_*d*_×*k*_*d*_^, respectively. *K* ∈ *ℝ*^*k*_*m*_×*k*_*d*_^ denotes the association matrix between miRNA modules and disease modules. Let *D* ∈ *ℝ*^*n*_*m*_×*n*_*d*_^ be the matrix storing the known miRNA-disease associations. The MDN-NMTF learns the representation vectors for miRNAs (*G*_*m*_) and disease (*G*_*d*_) by optimizing the following objective function.

$${\text{min}}_{G_{m},G_{d},S_{m},S_{d},K}\,\lambda_{1}\,{∥R_{m}-G_{m}\,S_{m}\,G_{m}^{\prime }∥}_{F}^{2}$$

$$\;+\lambda_{2}\,{∥Y⊙\left(D-G_{m}\,{\text{KG}}_{d}^{\prime }\right)∥}_{F}^{2}+\lambda_{3}\,{∥R_{d}-G_{d}\,S_{d}\,G_{d}^{\prime }∥}_{F}^{2}$$

$$\;+\alpha_{1}\,{∥G_{m}∥}_{F}^{2}+\alpha_{2}\,{∥G_{d}∥}_{F}^{2}+\beta_{1}\,\text{Tr}\,\left(G_{m}^{\prime }\,L_{m}\,G_{m}\right)$$

$$\;+\beta_{2}\,\text{Tr}\,\left(G_{d}^{\prime }\,L_{d}\,G_{d}\right)+\omega \,{∥K^{\prime }\,\text{K–I}∥}_{F}^{2}$$

(21) $$s.t.\;G_{m}>0,G_{d}>0,S_{m}>0,S_{d}>0,K>0$$

In Eq. (21), the term of ${||R_{m}-G_{m}\,S_{m}\,G_{m}^{\prime }||}_{F}^{2}$ captures the intrinsic module structure within the original miRNA similarity matrix. Because the values in *G*_*m*_ record the modules the miRNAs belong to and *S*_*m*_ records the relationship of these modules. ⊙ is the Hadamard product. The term of ${||Y⊙\left(D-G_{m}\,K\,G_{d}^{\prime }\right)||}_{F}^{2}$ indicates the miRNAs and diseases share similar relationship both in their original space and the latent space at the module level. We only want to use the known miRNA-disease information to learn their representation matrixes. Hence, let *Y* ∈ *ℝ*^*n*_*m*_×*n*_*d*_^ be a label weighted matrix [see Eq. (22)], where the elements of *Y* are set to 1 if the miRNA is known to associate with the disease. The elements of *Y* are set to 0.2 if the miRNA is known to not associate with the disease. Otherwise, the elements of *Y* are set to 0. Here, we set different weight for knowing to have or have no miRNA-disease associations. Because it is hard to prove that the miRNAs do not associate to certain diseases and some associations are temporarily not annotated due to the limitation of techniques.

(22) $$Y\,\left(m_{i},d_{j}\right)=\left\{ \begin{array}{cc} 1, & \text{if}\,D\,\left(m_{i},d_{j}\right)\,\text{is}\,\text{known}\,\text{and}\,D\,\left(m_{i},d_{j}\right)=1\\ 0.2, & \text{if}\,D\,\left(m_{i},d_{j}\right)\,\text{is}\,\text{known}\,\text{and}\,D\,\left(m_{i},d_{j}\right)=0\\ 0, & \text{if}\,D\,\left(m_{i},d_{j}\right)\,\mathrm{is}\,\text{unknown} \end{array}\right.$$

The terms of Tr (*G^′^_m⁢_L_m⁢_G_m_*) [see Eq. (19)] and Tr (*G^′^_d⁢_L_d⁢_G_d_*) [see Eq. (20)] are used to preserve the network structure of the original miRNA similarity network and disease similarity network, respectively. We introduce *L*_*m*_ and *L*_*d*_ to represent the dynamic neighborhood of miRNAs and diseases, respectively, (see section “The MDN-NMTF model”). Two terms of ${||G_{m}||}_{F}^{2}$ and ${||G_{d}||}_{F}^{2}$ are adopted to penalize the magnitudes of the *G*_*m*_ and *G*_*d*_ for avoiding overfitting. ${||K^{\prime }\,\text{K-I}||}_{F}^{2}$ is relaxed the constraint to *K’K* = *I*. *λ_1_*, *λ_2_*, and *λ_3_* are balance parameters of matrix tri-factorization. *α_1_* and *α_2_* are regularization term parameters. β*_1_* and β*_2_* are the dynamic neighborhood regularization parameters. *ω* is the *k*-constraint parameter. In this work, the values of *k*_*m*_, *k*_*d*_, *λ_1_*, *λ_2_*, *λ_3_*, *α_1_*, *α_2_*, β*_1_*, β*_2_*, and *ω* are set to 200, 200, 0.001, 5, 0.1, 0.2, 0.8, 90, 1.5, and 160, respectively (Supplementary Table 1).

### Computation of *S*_*m*_, *S*_*d*_, *G*_*m*_, *G*_*d*_, and *K*

To obtain the optimal solution of *S*_*m*_, *S*_*d*_, *G*_*m*_, *G*_*d*_, and *K* in the objective function of MDN-NMTF model Eq. (21), we take the partial derivative of the objective function with respect to *S*_*m*_, *S*_*d*_, *G*_*m*_, *G*_*d*_, and *K*, respectively. Following the *Karush–Kuhn–Tucker* (*KKT*) condition for the non-negativity of *S*_*m*_, *S*_*d*_, *G*_*m*_, *G*_*d*_, and *K* and setting the partial derivative equal to zero, we can update *S*_*m*_, *S*_*d*_, *G*_*m*_, *G*_*d*_, and *K* as follows.

(23) $$\begin{array}{c} S_{m}\leftarrow S_{m}⊙\frac{G_{m}^{{}^{\prime }}\,R_{m}\,G_{m}^{}}{G_{m}^{{}^{\prime }}\,G_{m}\,S_{m}\,G_{m}^{{}^{\prime }}\,G_{m}}. \end{array}$$

(24) $$\begin{array}{c} S_{d}\leftarrow S_{d}⊙\frac{G_{d}^{{}^{\prime }}\,R_{d}\,G_{d}^{}}{G_{d}^{{}^{\prime }}\,G_{d}\,S_{d}\,G_{d}^{{}^{\prime }}\,G_{d}}. \end{array}$$

(25) $$\begin{array}{c} G_{m}\leftarrow G_{m}⊙\frac{2\,\lambda_{1}\,R_{m}\,G_{m}^{}\,S_{m}+\lambda_{2}\,Y⊙{\text{DG}}_{d}\,K^{{}^{\prime }}+\beta_{1}\,{\text{AG}}_{m}}{2\,\lambda_{1}\,G_{m}\,S_{m}\,G_{m}^{{}^{\prime }}\,G_{m}\,S_{m}+\lambda_{2}\,Y⊙\left(G_{m}\,{\text{KG}}_{d}^{{}^{\prime }}\right)\,G_{d}\,K^{{}^{\prime }}+\alpha_{1}\,G_{m}+\beta_{1}\,D_{m}\,G_{m}}. \end{array}$$

(26) $$\begin{array}{c} G_{d}\leftarrow G_{d}⊙\frac{2\,\lambda_{3}\,R_{d}\,G_{d}\,S_{d}+\lambda_{2}\,Y^{{}^{\prime }}⊙D^{{}^{\prime }}\,G_{m}\,K+\beta_{2}\,{\text{BG}}_{d}}{2\,\lambda_{3}\,G_{d}\,S_{d}\,G_{d}^{\prime }\,G_{d}\,S_{d}+\lambda_{2}\,Y^{{}^{\prime }}⊙\left(G_{d}\,K\prime \,G_{m}^{\prime }\right)\,G_{m}\,K+\alpha_{2}\,G_{d}+\beta_{2}\,D_{d}\,G_{d}}. \end{array}$$

(27) $$\begin{array}{c} K\leftarrow K⊙\sqrt{\frac{\lambda_{2}\,G_{m}^{\prime }\,\left(Y⊙D\right)\,G_{d}+2\,\omega \,K}{\lambda_{2}\,G_{m}^{\prime }\,\left(Y⊙\left(G_{m}\,K\,G_{d}^{\prime }\right)\right)\,G_{d}+2\,\omega \,K\,K^{\prime }\,K}.} \end{array}$$

In this algorithm, ⊙ denotes the Hadamard product, and ÷ is entry-wise division for matrices. As shown in section “The MDN-NMTF model,” *A* and *B* are the dynamic nearest neighborhood matrix of miRNAs and diseases, respectively.

### Predicting miRNA-Disease Associations

After getting the low-rank matrixes *G*_*m*_, *K*, and *G*_*d*_, we rebuild matrix *D1* by the produce of the matrixes *G*_*m*_, *K*, and *G*_*d*_ (*D*1 = *G*_*m*_KG*_*d*_′*) to predict miRNA-disease associations. The elements in *D1* denote the probability between miRNAs and diseases. Following is the pseudocode of MDN-NMTF algorithm.

**Table:** 

Algorithm MDN-NMTF
Input: miRNA similarity *R*_*m*_; disease similarity *R*_*d*_; miRNA-disease association *D*; the parameters *λ_1_*, *λ_2_*, *λ_3_*, *α_1_*, *α_2_*, β*_1_*, β*_2_*, and *ω*.
Output: *G*_*m*_, *G*_*d*_, *S*_*m*_, *S*_*d*_, *K*, and *D1*
1: Initialize matrices *G*_*m*_, *G*_*d*_, *S*_*m*_, *S*_*d*_, and *K* with random non-negative matrices, while *S*_*m*_, *S*_*d*_ are symmetric matrices.
2: Calculate the dynamic neighbor matrices *A* and *B* by Eqs (17, 18), and then calculate the diagonal matrices *D*_*m*_ and *D*_*d*_, and the Laplacian matrix *L*_*m*_ and *L*_*d*_.
3: While objective function value in Eq. (21) not converge do
(1) Fix *G*_*m*_, *G*_*d*_, *S*_*d*_, and K and update *S*_*m*_ with Eq. (23).
(2) Fix *G*_*m*_, *G*_*d*_, *S*_*m*_, and K and update *S*_*d*_ with Eq. (24)
(3) Fix *G*_*d*_, *S*_*m*_, *S*_*d*_, and *K* and update *G*_*m*_ with Eq. (25).
(4) Fix *G*_*m*_, *S*_*m*_, *S*_*d*_, and *K* and update *G*_*d*_ with Eq. (26).
(5) Fix *G*_*m*_, *G*_*d*_, *S*_*m*_, and *S*_*d*_ and update *K* with Eq. (27).
end while
4: Rebuild miRNA-disease association matrix *D1* = *G*_*m*_KG*_*d*_′*.

In the third step of the while loop, each update iteration replaces the zero value in the matrices with 10^–9^ to guarantee the constraint condition in Eq. (21). The convergence condition is that the difference between two objective functions in the iteration is less than 10^–6^ or the number of iterations reaches the maximum number of iterations of 1,000.

### Predicting miRNA-Disease Associations With Modular Information

At the same time, we also extend MDN-NMTF to a new version (called MDN-NMTF2, see Algorithm MDN-NMTF2) by using the modular information to improve the miRNA-disease association prediction ability of MDN-NMTF. Since the factorized matrices *G*_*m*_ and *G*_*d*_ obtained from MDN-NMTF record the modules the miRNAs or diseases belong to. MDN-NMTF2 utilizes the *G*_*m*_ and *G*_*d*_ values to partition the miRNAs and diseases into different models. Given *G*_*m*_ ∈ *ℝ*^*n*_*m*_×*k*_*m*_^ and *G*_*d*_ ∈ *ℝ*^*n*_*d*_×*k*_*d*_^, there are *k*_*m*_ miRNA modules and *k*_*d*_ disease modules. The elements with relatively large values of each column of *G*_*m*_ (*G*_*d*_) is assigned to the members of the corresponding module. We calculate the threshold for each miRNA (i.e., each row *g*_*m*_(*i*,⋅) of *G*_*m*_) with:

(28) $$\begin{array}{c} \text{Th}\,\left(i\right)=\mu \,\left(i\right)+t\,\sigma \,\left(i\right) \end{array}$$

where $\mu \,\left(i\right)=\frac{1}{k_{m}}\,\sum_{k=1}^{k_{m}}g_{m}\,\left(i,k\right)$, $\sigma \,\left(i\right)=\sqrt{\frac{1}{k_{m}-1}\,\sum_{k=1}^{k_{m}}{\left(g_{m}\,\left(i,k\right)-\mu \,\left(i\right)\right)}^{2}}$, *t* is a given threshold. Based on this rule, we determined miRNA *m*_*i*_ as the *kth* module member if the entries of *g*_*m*_(*i*,*k*) are larger than *Th* (*i*). In the same way, the threshold for each disease [each row *g*_*d*_(*i*,⋅) of *G*_*d*_] can be calculated. According to the settings of Ma et al. (2020), we also set *t* = 1.5 to identify miRNA and disease modules with proper resolution.

Then we calculate the similarity of two miRNAs based on the module they belong to. If two miRNAs *m*_*i*_ and *m*_*j*_ belong to the same miRNA module, their similarity in the *kth* miRNA module (*ms*_*k*_) can be constructed as:

(29) $$\begin{array}{c} {\text{ms}}_{k}\,\left(m_{i},m_{j}\right)=\text{Sim}\,\left(m_{i},m_{j}\right),\text{where}\,m_{i}\in {\text{ms}}_{k},m_{j}\in {\text{ms}}_{k} \end{array}$$

The Sim(*u*, *v*) can be calculate as Eq.(30):

(30) $$\begin{array}{c} \text{Sim}\,\left(u,v\right)=\frac{\sum_{k=1}^{k_{m}}u_{k}\,v_{k}}{\sqrt{\sum_{k=1}^{k_{m}}u_{k}^{2}}\,\sqrt{\sum_{k=1}^{k_{m}}v_{k}^{2}}} \end{array}$$

Here, *k*_*m*_ represents the dimension of the vectors *u* and *v*. *u*_*k*_ and *v*_*k*_ represent the *k*th element of the vectors *u* and *v*. Similarly, if two diseases *d*_*i*_ and d*_*j*_* belong to the same disease module, we can construct their similarity in the *kth* disease module (*ds*_*k*_) as

(31) $$\begin{array}{c} {\text{ds}}_{k}\,\left(d_{i},d_{j}\right)=\text{Sim}\,\left(d_{i},d_{j}\right),\text{where}\,d_{i}\in {\text{ds}}_{k},d_{j}\in {\text{ds}}_{k} \end{array}$$

Based on the assumption that the miRNAs in the same modules are highly likely related to the same diseases, vice versa, we use Scorem_*k*_(*m*_*i*_,*d*_*j*_) to represent the correlation score between the disease *d*_*j*_ and the miRNA *m*_*i*_ in the *kth* miRNA module. It can be calculated according to Eq. (32):

(32) $$\begin{array}{c} {\text{Scorem}}_{k}\,\left(m_{i},d_{j}\right)=\frac{\sum_{q=1}^{n_{m}}{\text{ms}}_{k}\,\left(m_{i},m_{q}\right)\,D\,\left(q,j\right)}{\sum_{q=1}^{n_{m}}\text{Sim}\,\left(m_{i},m_{q}\right)} \end{array}$$

Where *D* ∈ *ℝ*^*n*_*m*_×*n*_*d*_^ is the matrix storing the known miRNA-disease associations. Thus, let *D*_*m*_ be the miRNA-disease associations that are predicted based on miRNA modules, which can be defined as:

(33) $$\begin{array}{c} D_{m}=\frac{1}{k_{m}}\,\sum_{i=1}^{k_{m}}{\mathrm{Scorem}}_{i} \end{array}$$

Similarly, we can get the correlation score (Scored_*k*_) in the *kth* disease module and predict miRNA-disease associations (*D*_*m*_) based on disease modules as follows:

(34) $$\begin{array}{c} {\text{Scored}}_{k}\,\left(m_{i},d_{j}\right)=\frac{\sum_{q=1}^{n_{d}}{\text{ds}}_{k}\,\left(d_{j},d_{q}\right)\,D\,\left(i,q\right)}{\sum_{q=1}^{n_{d}}\text{Sim}\,\left(d_{j},d_{q}\right)} \end{array}$$

(35) $$\begin{array}{c} D_{d}=\frac{1}{k_{d}}\,\sum_{j=1}^{k_{d}}{\text{Scored}}_{j} \end{array}$$

Here, *k*_*m*_ and *k*_*d*_, as shown in the previous section, denote the number of miRNA modules and disease modules. The final predicted miRNA-disease associations of MDN-NMTF2 can be calculated by:

(36) $$D\,2=D\,1^{*}+\frac{D_{m}^{*}}{2}+\frac{D_{d}^{*}}{2}$$

Here, ^∗^ denotes the Min-Max Normalization of the matrix. Following is the pseudocode of MDN-NMTF2.

**Table:** 

Algorithm MDN-NMTF2
Input: miRNA similarity matrix *R*_*m*_; disease similarity matrix *R*_*d*_ and miRNA-disease association matrix *D*; the parameters *λ_1_*, *λ_2_*, *λ_3_*, *α_1_*, *α_2_*, β*_1_*, β*_2_*, and *ω*.
Output: *D2*
1: Get *G*_*m*_, *G*_*d*_, *S*_*m*_, *S*_*d*_, *K*, and *D1 by* the Algorithm MDN-NMTF
2: Determine miRNA *m*_*i*_ as the *kth* module member if the entries of *g*_*m*_(*i*,*k*) are larger than *Th* (*i*) [see Eq. (28)]
3. Determine disease *d*_*i*_ as the *kth* module member if the entries of *g*_*d*_(*i*,*k*) are larger than *Th* (*i*) [see Eq. (28)]
4. Calculate similarity of two miRNAs If they belong to the same miRNA module by Eq. (29)
5. Calculate *D*_*m*_ as the miRNA-disease associations that are predicted based on miRNA modules [see Eq. (33)]
6. Calculate similarity of two diseases If they belong to the same disease module by Eq. (31)
7. Calculate *D*_*d*_ as the miRNA-disease associations that are predicted based on disease modules [see Eq. (35)]
8: Calculate the final predicted miRNA-disease associations *D2* = *D1^∗^+D_*m*_^∗^/2+D_*d*_^∗^/2*.

## Results

### Performance Evaluation

To evaluate the performances of MDN-NMTF and MDN-NMTF2, we compared them with four state-of-the-art methods (DNRLMF-MDA, IMCMDA, UBiRW, and GRNMF). The UBiRW uses an unbalanced Bi-Random walk on the heterogeneous network to propagate information and to infer potential miRNA-disease associations. DNRLMF-MDA, IMCMDA, and GRNMF are three latest network embedding-based methods. DNRLMF-MDA adopts a dynamic neighborhood regularized logistic matrix factorization method to predict potential miRNA-disease associations. IMCMDA uses Inductive matrix completion for miRNA-disease association prediction. GRNMF infer possible associations for all disease by a graph regularized non-negative matrix factorization framework. Considering there is no available interaction observed for new diseases or miRNAs, GRNMF develops a preprocessing step to construct the miRNA-disease associations according to the neighbors’ information. We implemented cross-validation under two different settings to evaluate the performance of the proposed methods. The two different settings are 5-fold randomly zeroing and single-column zeroing. For 5-fold randomly zeroing cross-validation, all the known miRNA-disease associations are randomly and equally divided into five non-overlapping parts. In each round, one of the five parts is for testing and the corresponding values in matrix *D* are cleared as 0, and the other four parts are as positive samples for training. Note that the miRNA and disease similarity network should be recalculated in each round. Single-column zeroing is to clear all miRNA-disease associations of a particular column of diseases and take them as testing data, others as training sets, and finally sum all AUCs to get the mean value. We repeat the cross-validation 20 times on four different datasets and show the average values in the following sections. For HMDD2.0-You, HMDD2.0-Lan, HMDD2.0-Yan and HMDD3.0 datasets, we select the way illustrated in section “Materials” to calculate the miRNA similarity network and disease similarity network.

To make the comparison fair, we tuned the parameters for every method to perform them the best in all of our experiments through randomly zeroing 5-fold cross-validation on HMDD2.0-You, HMDD2.0-Lan, and HMDD2.0-Yan. The detailed information, please see the online Supplementary Files (Supplementary Table 2).

### Randomly Zeroing Cross-Validation

As we can see from Table 2, MDN-NMTF and MDN-NMTF2 possess the highest two performance among the four methods on all the four datasets in terms of AUC values. On HMDD2.0-You dataset, compared with other methods (DNRLMF-MDA: 0.9301 ± 0.0036, IMCMDA: 0.8285 ± 0.0068, UBiRW: 0.9196 ± 0.0036, and GRNMF: 0.9031 ± 0.0049), the AUC values of MDN-NMTF and MDN-NMTF2 achieve 0.9335 ± 0.0037 and 0.9354 ± 0.0035, respectively. On HMDD2.0-Yan dataset, the prediction performance of MDN-NMTF and MDN-NMTF2 are the best two because their AUC values are 0.9409 ± 0.0030 and 0.9424 ± 0.0033, compared with other methods (DNRLMF-MDA: 0.9384 ± 0.0031, IMCMDA: 0.8045 ± 0.0062, UBiRW: 0.9191 ± 0.0030, GRNMF: 0.9153 ± 0.0045). On HMDD2.0-Lan dataset, the AUC values of MDN-NMTF and MDN-NMTF2 are 0.9391 ± 0.0033 and 0.9415 ± 0.0033, which are both superior to the other results of DNRLMF-MDA (0.9369 ± 0.0030), IMCMDA (0.7216 ± 0.0072), UBiRW (0.9198 ± 0.0032), and GRNMF (0.9157 ± 0.0044). On HMDD3.0 dataset, the AUC values of the MDN-NMTF and MDN-NMTF2 are 0.9435 ± 0.0021 and 0.9467 ± 0.0020, which is better than that of DNRLMF-MDA method (0.9390 ± 0.0015), that of IMCMDA method (0.6572 ± 0.0052), that of UBiRW method (0.9280 ± 0.0016) and that of GRNMF method (0.9247 ± 0.0023). We observe that DNRLMF-MDA leads to the highest performance among the four existing methods. It may be the DNRLMF-MDA method adopts a dynamic neighborhood regularized logistic matrix factorization method to predict potential miRNA-disease associations. Both our MDN-NMTF and MDN-NMTF2 methods and the DNRLMF-MDA method utilize the dynamic neighborhood regularized restriction to construct the miRNA and disease feature vectors. We observe that our MDN-NMTF and MDN-NMTF2 methods outperform DNRLMF-MDA. It can be partially attributed to the high quality of miRNA and disease features extracted by our MDN-NMTF and MDN-NMTF2 method from the heterogeneous network under the consideration of the networks’ module properties. MDN-NMTF2 employs the miRNA and disease features extracted by MDN-NMTF method to partition miRNA and disease modules. It infers potential miRNA-disease associations by considering the miRNAs’ neighbors and diseases’ neighbors in the same modules, which makes the MDN-NMTF2 method achieves a clear improvement than the MDN-NMTF method when predicting the missing miRNA-disease associations.

**TABLE 2:** The AUC values for the models on different databases by randomly zeroing cross validation.

**Methods**	**HMDD2.0-You**	**HMDD2.0-Yan**	**HMDD2.0-Lan**	**HMDD3.0**
MDN-NMTF	0.9335 ± 0.0037	0.9409 ± 0.0030	0.9391 ± 0.0033	0.9435 ± 0.0021
MDN-NMTF2	**0.9354 ± 0.0035**	**0.9424 ± 0.0033**	**0.9415 ± 0.0033**	**0.9467 ± 0.0020**
DNRLMF-MDA	0.9301 ± 0.0036	0.9384 ± 0.0031	0.9369 ± 0.0030	0.9390 ± 0.0015
IMCMDA	0.8285 ± 0.0068	0.8045 ± 0.0062	0.7216 ± 0.0072	0.6572 ± 0.0052
UBiRW	0.9196 ± 0.0036	0.9191 ± 0.0030	0.9198 ± 0.0032	0.9280 ± 0.0016
GRNMF	0.9031 ± 0.0049	0.9153 ± 0.0045	0.9157 ± 0.0044	0.9247 ± 0.0023

*It can be seen from the picture that the AUC values of our methods MDN-NMTF and MDN-NMTF2 on four datasets are both higher than the other four methods, and our extended method MDN-NMTF2 is a little higher than the original method MDN-NMTF. The highest AUC values of all methods in different datasets are in bold.*

**FIGURE 2:**
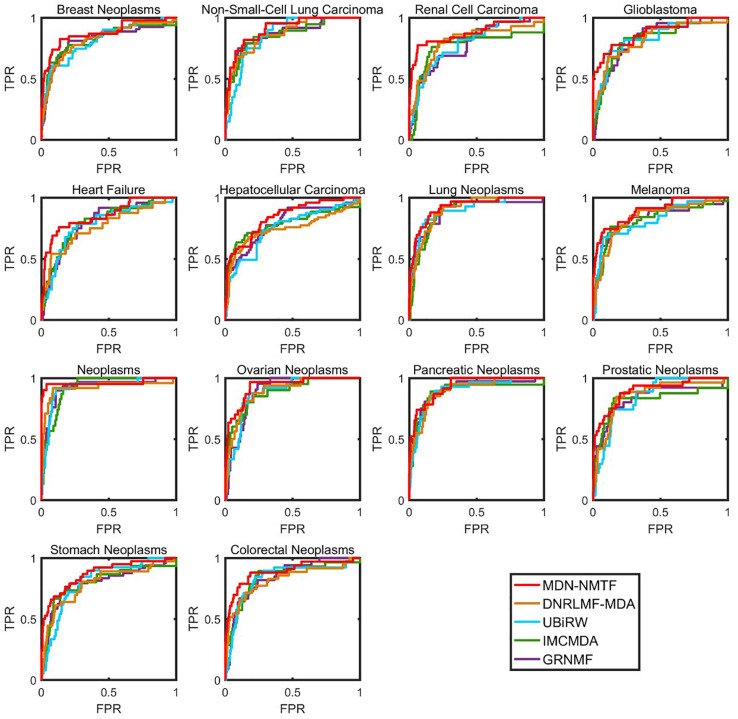
The ROC curves of MDN-NMTF and other four methods for 14 diseases on HMDD2.0-Yan Dataset. The figure shows that the ROC curves of MDN-NMTF in 14 diseases are all higher than that of the other four methods.

Besides, we test the performance of each method on 14 common diseases related to at least 110 miRNAs. Figure 2 illustrates the Receiver operating characteristics curves of each method on the 14 disease. Table 3 GRNMF lists the corresponding area under the curves (AUC). Both results show that MDN-NMTF outperforms the other four methods for all the 14 diseases.

**TABLE 3:** AUC values of MDN-NMTF and other four compared methods for the 14 diseases on HMDD2.0-Yan Dataset.

**Disease name**	**MDN-NMTF**	**DNRLMF-MDA**	**IMCMDA**	**UBiRW**	**GRNMF**
Breast neoplasms	**0.8732**	0.8274	0.8340	0.8194	0.8183
Non-small-cell lung carcinoma	**0.8989**	0.8748	0.8658	0.8614	0.8582
Renal cell carcinoma	**0.8699**	0.8089	0.7936	0.7592	0.7846
Glioblastoma	**0.8740**	0.8280	0.8414	0.8248	0.8336
Heart failure	**0.8528**	0.7797	0.7864	0.7962	0.8120
Hepatocellular carcinoma	**0.8408**	0.7585	0.7588	0.7916	0.7846
Lung neoplasms	**0.9207**	0.9077	0.8963	0.8922	0.8885
Melanoma	**0.8898**	0.8375	0.8211	0.8216	0.8251
Neoplasms	**0.9555**	0.9253	0.9227	0.9223	0.9264
Ovarian neoplasms	**0.9262**	0.8941	0.8863	0.8835	0.8885
Pancreatic neoplasms	**0.9274**	0.9035	0.8943	0.8886	0.9057
Prostatic neoplasms	**0.8919**	0.8623	0.8395	0.8184	0.8261
Stomach neoplasms	**0.8641**	0.8054	0.8164	0.8055	0.8071
Colorectal neoplasms	**0.8899**	0.8292	0.8350	0.8463	0.8425

*The table shows that the AUC values of MDN-NMTF in 14 diseases are all higher than that of the other four methods. The highest AUC values of all methods in different datasets are in bold.*

### Single-Column Zeroing Cross Validation

It still is a challenging task to infer miRNA associations for a new disease. To assess whether the MDN-NMTF and MDN-NMTF2 methods can successfully predict related miRNA for new diseases, we perform single-column zeroing cross-validation. Table 4 lists the AUC values of different methods on four datasets. The AUC values of MDN-NMTF and MDN-NMTF2 still control the highest two in the four datasets. Compared to DNRLMF-MDA that has relatively better performance among the four existing methods, the MDN-NMTF method achieves 1.04% improvement on HMDD2.0-You dataset, 0.37% improvement on HMDD2.0-Yan dataset, 0.72% improvement on HMDD2.0-Lan dataset, and 1.18% improvement on HMDD3.0 dataset. The results prove that our methods considering the intrinsic module structure of miRNA and disease networks can extract the high quality of miRNA and disease features to predict related miRNAs for new diseases successfully. We observe that MDN-NMTF2 has a little lower performance than MDN-NMTF across the four datasets. It may be MDN-NMTF2 fails to infer the associations for new disease from the miRNA in the same modules.

**TABLE 4:** The AUC values of each method on four different datasets by single-column zeroing cross validation.

**Methods**	**HMDD2.0-You**	**HMDD2.0-Yan**	**HMDD2.0-Lan**	**HMDD3.0**
MDN-NMTF	**0.8570 ± 0.1223**	**0.8482 ± 0.1265**	0.8445 ± 0.1339	**0.8917 ± 0.1108**
MDN-NMTF2	0.8561 ± 0.1240	0.8473 ± 0.1292	**0.8447 ± 0.1342**	0.8896 ± 0.1142
DNRLMF-MDA	0.8482 ± 0.1355	0.8451 ± 0.1431	0.8385 ± 0.1487	0.8813 ± 0.1181
IMCMDA	0.8329 ± 0.1297	0.8214 ± 0.1290	0.8158 ± 0.1357	0.8781 ± 0.1308
UBiRW	0.8512 ± 0.1343	0.8403 ± 0.1356	0.8326 ± 0.1499	0.8794 ± 0.1341
GRNMF	0.7833 ± 0.1505	0.7504 ± 0.1618	0.7895 ± 0.1465	0.8245 ± 0.1502

*The table lists the AUC values of different methods on four different datasets. The AUC values of MDN-NMTF and MDN-NMTF2 are still the highest two. The results prove that our method considering the intrinsic module structure of miRNA and disease networks can improve the performance on miRNA-disease association predictions. The bold AUC values represent the highest AUC value of all method in the four different datasets.*

### Case Study

To further illustrate the performance of MDN-NMTF, we evaluate its miRNA prediction ability for some cancer types, such as Stomach Neoplasms (gastric Neoplasms) and Lymphoma. The dbDEMC database and miRCancer database are used as the benchmark datasets.

Among cancer-related deaths worldwide, Stomach Neoplasms ranks the third. Increasing evidence indicates that many miRNAs interact with Stomach Neoplasms by regulating the related genes of Stomach Neoplasms. Table 5 demonstrates the top 50 predicted novel Stomach Neoplasms-related miRNAs predicted by MDN-NMTF on HMDD2.0-Yan dataset and the corresponding evidence. Table 5 shows 35 of the 50 miRNAs are validated by dbDEMC database and miRCancer database. The remaining 15 miRNAs are all found to be related to human diseases in the literature. miR-181b modulates multidrug resistance by targeting BCL2 in human cancer cell lines (Zhu et al., 2010). MicroRNA-125b affects the proliferation of gastric cancer cells (Yang et al., 2013). miR-15b and miR-16 modulate multidrug resistance by targeting BCL2 in human gastric cancer cells (Xia et al., 2008). miR-101-2, miR-125b-2, and miR-451a act as potential tumor suppressors in primary GCs as well as in GC-derived AGS cells (Riquelme et al., 2016). MicroRNA-181b targets cAMP-responsive element-binding protein 1 in gastric adenocarcinomas (Chen L. et al., 2012). Plasma miRNA-199a-3p and miRNA-151-5p are significantly elevated (*p* < 0.05) and are significantly reduced after surgery (*p* < 0.05) in gastric cancer patients (Li et al., 2012). Genomic loss of miR-486 regulates tumor progression and the OLFM4 antiapoptotic factor in gastric cancer (Oh et al., 2011). Lack of microRNA-101 causes E-cadherin functional deregulation through EZH2 upregulation in intestinal gastric cancer (Carvalho et al., 2012). Significant associations are found between hypermethylation of the hsa-miR-124a and tumor size, differentiation, lymphatic metastasis, and invasion depth (Pei et al., 2011). miR-103, miR-21, miR-145, miR-106b, miR-146a, and miR-148a separate node-positive from node-negative gastric cancers (Tchernitsa et al., 2010). miR-7 is a novel mechanism by which the inflammatory response promotes gastric tumorigenesis (Kong et al., 2012).

**TABLE 5:** Top 50 Related miRNAs of Stomach Neoplasms predicted by MDN-NMTF on HMDD2.0-Yan Dataset.

**Top1-25 miRNA**	**Evidence**	**Top26-50 miRNA**	**Evidence**
hsa-mir-21	dbDEMC, miRCancer	hsa-mir-199a-1	PMID:22956063
hsa-mir-214	dbDEMC, miRCancer	hsa-mir-22	dbDEMC, miRCancer
hsa-mir-200b	dbDEMC, miRCancer	hsa-mir-375	dbDEMC, miRCancer
hsa-mir-200c	miRCancer	hsa-mir-486	PMID:21415212
hsa-mir-182	dbDEMC, miRCancer	hsa-mir-106a	dbDEMC, miRCancer
hsa-mir-221	dbDEMC, miRCancer	hsa-mir-16-1	miRCancer
hsa-mir-181b-1	PMID:20162574	hsa-mir-222	dbDEMC, miRCancer
hsa-mir-148a	dbDEMC, miRCancer	hsa-mir-101-1	PMID:22450781
hsa-mir-34c	miRCancer	hsa-mir-10b	dbDEMC, miRCancer
hsa-mir-146b	miRCancer	hsa-mir-195	dbDEMC, miRCancer
hsa-mir-34a	dbDEMC, miRCancer	hsa-mir-141	dbDEMC, miRCancer
hsa-mir-125b-1	PMID:23128435	hsa-mir-101-2	PMID:26458815
hsa-mir-200a	dbDEMC, miRCancer	hsa-mir-146a	miRCancer
hsa-mir-31	dbDEMC, miRCancer	hsa-mir-199a-2	PMID:22956063
hsa-mir-145	dbDEMC, miRCancer	hsa-mir-106b	dbDEMC, miRCancer
hsa-mir-126	dbDEMC, miRCancer	hsa-mir-143	dbDEMC, miRCancer
hsa-mir-34b	miRCancer	hsa-mir-124-1	PMID:21365509
hsa-mir-16-2	PMID:18449891	hsa-mir-124-2	PMID:21365509
hsa-mir-125b-2	PMID:26458815	hsa-mir-103a-2	PMID:20726036
hsa-mir-107	dbDEMC, miRCancer	hsa-mir-130a	dbDEMC, miRCancer
hsa-mir-223	dbDEMC, miRCancer	hsa-mir-27b	dbDEMC, miRCancer
hsa-mir-183	dbDEMC, miRCancer	hsa-mir-155	dbDEMC, miRCancer
hsa-mir-27a	dbDEMC, miRCancer	hsa-mir-335	miRCancer
hsa-mir-25	dbDEMC, miRCancer	hsa-mir-151a	PMID:22956063
hsa-mir-181b-2	PMID:22539488	hsa-mir-7-1	PMID:22139078

*From the table, 35 of the 50 miRNAs are validated by dbDEMC database and miRCancer database. The remaining 15 miRNAs are all found to be disease-related in the literature.*

Lymphoma is a type of cancer that begins in immune system cells. It is one of the top 10 deadly diseases. Table 6 shows the result of top 50 Lymphoma-related miRNAs detected by MDN-NMTF on the HMDD2.0-Yan dataset. It shows that 39 of the 50 miRNAs are validated by dbDEMC database and miRCancer database. The remaining 11 miRNAs are all found to be disease-related in the literature. The plasma miR-92a value could be a novel biomarker not only for diagnosis but also for monitoring lymphoma patients after chemotherapy (Ohyashiki et al., 2011). Compared with healthy canine peripheral blood mononuclear cells and normal lymph nodes, mir-181a shows a decreased expression level (Uhl et al., 2011). miR-26a is repressed by MYC (Sander et al., 2009). The down-regulation of miR-16, miR-26a, miR-101, miR-29c, and miR138 in the *t*(14;18)-negative FL (follicular lymphoma) subset is associated with profound mRNA expression changes of potential target genes involving cell cycle control, apoptosis and B-cell differentiation. miR-16 targets CHEK1 showing increased expression on the protein level in *t*(14;18)-negative FL, while reducing TCL1A expression, in line with a partial loss of the germinal center B-cell phenotype in this FL subset (Leich et al., 2011). mir-499a is deregulated hypermutations (Navarro et al., 2009). miR-24 is overexpressed (Gibcus et al., 2009). A distinct set of five microRNAs (miR-150, miR-550, miR-124a, miR-518b, and miR-539) is shown to be differentially expressed in gastritis as opposed to MALT lymphoma (Thorns et al., 2012). miR-125b-5p not only regulates tumor growth *in vivo* but also increases cellular resistance to proteasome inhibitors via modulation of MAD4 (Manfè et al., 2013).

**TABLE 6:** Top 50 Related miRNAs of Lymphoma predicted by MDN-NMTF on HMDD2.0-Yan Dataset.

**Top1-25 miRNA**	**Evidence**	**Top26-50 miRNA**	**Evidence**
hsa-mir-17	dbDEMC, miRCancer	hsa-mir-363	dbDEMC
hsa-mir-20a	dbDEMC, miRCancer	hsa-mir-150	dbDEMC, miRCancer
hsa-mir-155	dbDEMC, miRCancer	hsa-mir-126	dbDEMC
hsa-mir-18a	dbDEMC, miRCancer	hsa-mir-200b	dbDEMC
hsa-mir-19a	dbDEMC, miRCancer	hsa-mir-184	dbDEMC
hsa-mir-19b-1	miRCancer	hsa-mir-200a	dbDEMC
hsa-mir-92a-1	PMID:21383985	hsa-mir-499a	PMID:19690137
hsa-mir-15a	dbDEMC, miRCancer	hsa-mir-34a	dbDEMC
hsa-mir-146a	dbDEMC	hsa-mir-210	dbDEMC
hsa-mir-19b-2	miRCancer	hsa-mir-200c	dbDEMC
hsa-mir-16-1	miRCancer	hsa-mir-205	dbDEMC
hsa-mir-16-2	miRCancer	hsa-mir-145	dbDEMC
hsa-mir-21	dbDEMC, miRCancer	hsa-mir-24-1	PMID:19177201
hsa-mir-92a-2	PMID:21383985	hsa-mir-125b-1	dbDEMC
hsa-mir-181a-1	dbDEMC	hsa-mir-20b	dbDEMC
hsa-mir-181a-2	PMID:21910161	hsa-mir-125a	dbDEMC
hsa-mir-26a-2	dbDEMC	hsa-mir-124-1	PMID:22395483
hsa-mir-26a-1	PMID:19197161	hsa-mir-141	dbDEMC
hsa-mir-122	dbDEMC	hsa-mir-125b-2	PMID:23527180
hsa-mir-101-1	PMID:21960592	hsa-mir-18b	dbDEMC
hsa-mir-101-2	PMID:21960592	hsa-mir-138-2	dbDEMC
hsa-mir-342	dbDEMC	hsa-mir-29c	dbDEMC
hsa-mir-486	dbDEMC	hsa-mir-138-1	PMID:21960592
hsa-mir-203	dbDEMC	hsa-mir-708	dbDEMC
hsa-mir-223	dbDEMC, miRCancer	hsa-mir-143	dbDEMC

*From the table, 39 of the 50 miRNAs are validated by dbDEMC database and miRCancer database. The remaining 11 miRNAs are all found to be disease-related in the literature.*

### Module Analysis

**FIGURE 3:**
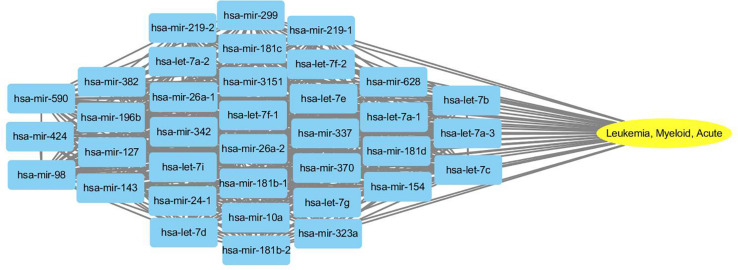
An example of miRNA module detected by MDN-NMTF2 on HMDD2.0-Yan Dataset. The figure shows that all 36 miRNAs in the module are related to Leukemia Myeloid Acute.

**FIGURE 4:**
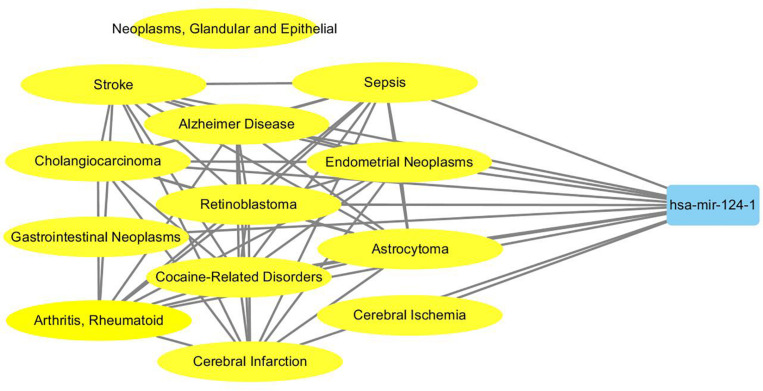
An example of disease module detected by MDN-NMTF2 on HMDD2.0-Yan Dataset. The figure shows that 12 of 13 diseases in the module are related to a miRNA has-mir-124-1.

To probe why the modules help the MDN-NMTF2 to obtain better result, we analyze the miRNA or disease modules detected by MDN-NMTF2 on the dataset HMDD2.0-Yan (Supplementary Texts 3, 4). Table 7 lists the details of these modules. There are 127 miRNA modules with more than one member after removing the modules. The average size of these modules is 40. There are 142 disease modules with more than one member and their average size is 22. The average function similarity of the members in the miRNA modules was 0.4409, which was 113.20% higher than the average value of 0.2068 of the whole miRNA function similarity network (Supplementary Text 5). Similarly, the average function similarity of the disease modules was 0.0939, which was 160.11% higher than the average value of 0.0361 of the whole disease function similarity network (Supplementary Text 6). It suggests that the miRNA modules and disease modules detected by MDN-NMTF2 consist of members with similar functions. We also find that average 82% of miRNAs in the same module are related to the same disease (Supplementary Text 7). Figure 3 shows an example of miRNA module that consists of 36 miRNAs. All of these miRNAs are associated with Leukemia Myeloid Acute. On the other hand, 61% of the disease in the same module relate to the same miRNA (Supplementary Text 8). Figure 4 illustrates an example of disease module with 13 members. 12 of 13 diseases in the module relate to a common miRNA has-mir-124-1 that expresses in human embryonic stem cells. Hence, the MDN-NMTF2 infers miRNA-disease associations from miRNAs or diseases in the same modules, which helps it achieve better prediction results.

**TABLE 7:** miRNA modules and disease modules detected by MDN-NMTF2 on HMDD2.0-Yan Dataset.

**Modules**	**NM**	**AvgSize**	**AvgSim**	**AvgPc**
miRNA	127	40	0.4409	82.20%
disease	142	22	0.0939	61.28%

*This table list the detailed information of the miRNA modules and disease modules detected by MDN-NMTF2 on the HMDD2.0-Yan dataset. Here, NM is the number of modules with more than one member. AvgSize is the average size of the modules. AvgSim is the average function similarity of the members in the modules. AvgPc is the average percentage of miRNAs (diseases) in the same modules share associations with common diseases (miRNA). It can be seen from the table that the average function similarity of the members in the miRNA modules was 0.4409, the values in the disease modules was 0.0939. Meanwhile, average 82% of miRNAs in the same module are related to the same disease and 61% of the disease in the same module is related to the same miRNA.*

## Conclusion

Inferring miRNA-disease associations is a crucial step to manifest principles of disease prevention, disease diagnosis and drug development. In this study, we have presented a novel method named MDN-NMTF to predict miRNA-disease associations. It constructs a heterogeneous network of disease similarity network, miRNA functional similarity network and a known miRNA-disease association network. After that, it learns the vector representation for miRNAs and diseases in the heterogeneous network by a matrix tri-factorization method under the constraint of the module structure and dynamic neighborhood. Finally, MDN-NMTF predicts novel miRNA-disease association probability by the product of the miRNA and disease latent vectors. At the same time, we also extend MDN-NMTF to a new version (called MDN-NMTF2) by using the modular information. Compared with the previous network propagation-based method, like UBiRW, MDN-NMTF, and MDN-NMTF2 project miRNAs and diseases to a latent space. It can successfully integrate diverse biological information of miRNAs and diseases to predict miRNA-disease associations. Compared with the network embedding-based methods, like DNRLMF-MDA, IMCMDA and GRNMF, and MDN-NMTF and MDN-NMTF2 consider the module properties of miRNAs and diseases in the course of learning vector representation, which can maximally preserves the heterogeneous network structural information and the network properties. In particular, MDN-NMTF2 not only considers the modularity in the feature learning process but also uses the miRNA module and disease module information when reconstructing the miRNA-disease association matrix. We test our methods and the other four existing methods on four different datasets by implementing randomly zero cross-validation and single-column zero cross-validation. The results show that our methods outperform the state-of-the-art methods not only on predicting the missing miRNA-disease associations but also on recommending related miRNA for new diseases.

## Data Availability Statement

Publicly available datasets were analyzed in this study. This data can be found here: https://github.com/weiba/MDN-NMTF.

## Author Contributions

WP and JD obtained and analyzed miRNA-related data, disease-related data, and miRNA-disease associations. WP, JD, WD, and WL designed the new method MDN-NMTF and analyzed the results. WP and JD drafted the manuscript together. WP, JD, WD, and WL participated in revising the draft. All authors have read and approved the manuscript.

## Conflict of Interest

The authors declare that the research was conducted in the absence of any commercial or financial relationships that could be construed as a potential conflict of interest.

## Footnotes

1 http://www.cuilab.cn/hmdd
