# Supplementary material for: Predicting miRNA-Disease Association Based on Modularity Preserving Heterogeneous Network Embedding
Source: Front Cell Dev Biol. 2021 Jun 10;9:603758. doi: 10.3389/fcell.2021.603758 (PMC8223753; doi:10.3389/fcell.2021.603758)
Supplement: Supplementary file 1 [file Table_1.DOCX]

Table S1. The parameter adjustment file of MDN-NMTF and other four methods of three datasets on randomly zeroing cross validation.

MDN-NMTF:

| *d_m_* | *d_d_* | *λ_1_* | *λ_2_* | *λ_3_* | *α_1_* | *α_2_* | *β_1_* | *β_2_* | *ω* | *ε* | HMDD2.0-You | HMDD2.0-Yan | HMDD2.0-Lan |
| --- | --- | --- | --- | --- | --- | --- | --- | --- | --- | --- | --- | --- | --- |
| 10 | 200 | 0.001 | 5 | 0.001 | 0.2 | 1 | 105 | 1.2 | 180 | 0.56 | 0.927822 | 0.930911 | 0.930978 |
| 50 | 200 | 0.001 | 5 | 0.001 | 0.2 | 1 | 105 | 1.2 | 180 | 0.56 | 0.932086 | 0.939380 | 0.937431 |
| 100 | 200 | 0.001 | 5 | 0.001 | 0.2 | 1 | 105 | 1.2 | 180 | 0.56 | 0.932677 | 0.939808 | 0.937993 |
| 150 | 200 | 0.001 | 5 | 0.001 | 0.2 | 1 | 105 | 1.2 | 180 | 0.56 | 0.932541 | 0.939803 | 0.938121 |
| 200 | 200 | 0.001 | 5 | 0.001 | 0.2 | 1 | 105 | 1.2 | 180 | 0.56 | 0.932997 | 0.940198 | 0.938990 |
| 220 | 200 | 0.001 | 5 | 0.001 | 0.2 | 1 | 105 | 1.2 | 180 | 0.56 | 0.932771 | 0.940361 | 0.938936 |
| 200 | 50 | 0.001 | 5 | 0.001 | 0.2 | 1 | 105 | 1.2 | 180 | 0.56 | 0.932264 | 0.939068 | 0.937223 |
| 200 | 100 | 0.001 | 5 | 0.001 | 0.2 | 1 | 105 | 1.2 | 180 | 0.56 | 0.932672 | 0.939259 | 0.937699 |
| 200 | 150 | 0.001 | 5 | 0.001 | 0.2 | 1 | 105 | 1.2 | 180 | 0.56 | 0.933090 | 0.939969 | 0.937616 |
| 200 | 190 | 0.001 | 5 | 0.001 | 0.2 | 1 | 105 | 1.2 | 180 | 0.56 | 0.932730 | 0.940433 | 0.938825 |
| 200 | 210 | 0.001 | 5 | 0.001 | 0.2 | 1 | 105 | 1.2 | 180 | 0.56 | 0.932843 | 0.940517 | 0.938810 |
| 200 | 200 | 0.001 | 5 | 0.001 | 0.2 | 1 | 105 | 1.2 | 180 | 0.4 | 0.926074 | 0.937187 | 0.936892 |
| 200 | 200 | 0.001 | 5 | 0.001 | 0.2 | 1 | 105 | 1.2 | 180 | 0.5 | 0.932072 | 0.940066 | 0.939314 |
| 200 | 200 | 0.001 | 5 | 0.001 | 0.2 | 1 | 105 | 1.2 | 180 | 0.6 | 0.933205 | 0.939174 | 0.937540 |
| 200 | 200 | 0.001 | 5 | 0.001 | 0.2 | 1 | 105 | 1.2 | 180 | 0.7 | 0.929238 | 0.934241 | 0.933038 |
| 200 | 200 | 0.001 | 5 | 0.01 | 0.2 | 1 | 105 | 1.2 | 180 | 0.56 | 0.933227 | 0.940407 | 0.938751 |
| 200 | 200 | 0.001 | 5 | 0.1 | 0.2 | 1 | 105 | 1.2 | 180 | 0.56 | 0.933179 | 0.940450 | 0.939056 |
| 200 | 200 | 0.001 | 5 | 1 | 0.2 | 1 | 105 | 1.2 | 180 | 0.56 | 0.932684 | 0.940514 | 0.939173 |
| 200 | 200 | 0.01 | 5 | 0.1 | 0.2 | 1 | 105 | 1.2 | 180 | 0.56 | 0.933064 | 0.940366 | 0.938984 |
| 200 | 200 | 0.1 | 5 | 0.1 | 0.2 | 1 | 105 | 1.2 | 180 | 0.56 | 0.933065 | 0.940557 | 0.938924 |
| 200 | 200 | 0.001 | 0.1 | 0.1 | 0.2 | 1 | 105 | 1.2 | 180 | 0.56 | 0.887170 | 0.879512 | 0.889474 |
| 200 | 200 | 0.001 | 1 | 0.1 | 0.2 | 1 | 105 | 1.2 | 180 | 0.56 | 0.921811 | 0.924543 | 0.923285 |
| 200 | 200 | 0.001 | 10 | 0.1 | 0.2 | 1 | 105 | 1.2 | 180 | 0.56 | 0.929593 | 0.938320 | 0.937583 |
| 200 | 200 | 0.001 | 5 | 0.1 | 0.2 | 1 | 105 | 1.2 | 10 | 0.56 | 0.930135 | 0.936379 | 0.935274 |
| 200 | 200 | 0.001 | 5 | 0.1 | 0.2 | 1 | 105 | 1.2 | 100 | 0.56 | 0.933183 | 0.939922 | 0.938843 |
| 200 | 200 | 0.001 | 5 | 0.1 | 0.2 | 1 | 105 | 1.2 | 160 | 0.56 | 0.933372 | 0.940617 | 0.939197 |
| 200 | 200 | 0.001 | 5 | 0.1 | 0.2 | 1 | 105 | 1.2 | 1000 | 0.56 | 0.933653 | 0.940598 | 0.939260 |
| 200 | 200 | 0.001 | 5 | 0.1 | 0.2 | 1 | 105 | 0.1 | 160 | 0.56 | 0.927760 | 0.938231 | 0.938403 |
| 200 | 200 | 0.001 | 5 | 0.1 | 0.2 | 1 | 105 | 1 | 160 | 0.56 | 0.932577 | 0.940464 | 0.939316 |
| 200 | 200 | 0.001 | 5 | 0.1 | 0.2 | 1 | 105 | 1.5 | 160 | 0.56 | 0.933317 | 0.940417 | 0.939007 |
| 200 | 200 | 0.001 | 5 | 0.1 | 0.2 | 1 | 105 | 5 | 160 | 0.56 | 0.932551 | 0.938104 | 0.935683 |
| 200 | 200 | 0.001 | 5 | 0.1 | 0.2 | 1 | 10 | 1.5 | 160 | 0.56 | 0.925827 | 0.935086 | 0.934317 |
| 200 | 200 | 0.001 | 5 | 0.1 | 0.2 | 1 | 90 | 1.5 | 160 | 0.56 | 0.933319 | 0.940563 | 0.939179 |
| 200 | 200 | 0.001 | 5 | 0.1 | 0.2 | 1 | 200 | 1.5 | 160 | 0.56 | 0.932264 | 0.938246 | 0.936267 |
| 200 | 200 | 0.001 | 5 | 0.1 | 0.2 | 1 | 1000 | 1.5 | 160 | 0.56 | 0.926026 | 0.930209 | 0.929107 |
| 200 | 200 | 0.001 | 5 | 0.1 | 0.2 | 0.1 | 90 | 1.5 | 160 | 0.56 | 0.932980 | 0.937414 | 0.934931 |
| 200 | 200 | 0.001 | 5 | 0.1 | 0.2 | 0.7 | 90 | 1.5 | 160 | 0.56 | 0.933478 | 0.940817 | 0.939273 |
| **200** | **200** | **0.001** | **5** | **0.1** | **0.2** | **0.8** | **90** | **1.5** | **160** | **0.56** | 0.933732 | **0.940893** | **0.939337** |
| 200 | 200 | 0.001 | 5 | 0.1 | 0.2 | 10 | 90 | 1.5 | 160 | 0.56 | 0.924520 | 0.930752 | 0.929645 |
| 200 | 200 | 0.001 | 5 | 0.1 | 0.1 | 0.8 | 90 | 1.5 | 160 | 0.56 | **0.933957** | 0.940472 | 0.939017 |
| 200 | 200 | 0.001 | 5 | 0.1 | 0.5 | 0.8 | 90 | 1.5 | 160 | 0.56 | 0.933373 | 0.940463 | 0.939325 |
| 200 | 200 | 0.001 | 5 | 0.1 | 1 | 0.8 | 90 | 1.5 | 160 | 0.56 | 0.933438 | 0.940573 | 0.939167 |

DNRLMF-MDA:

| *c* | *r* | *λ_m_* | *λ_d_* | *α* | *β* | *ε_1_* | HMDD2.0-You | HMDD2.0-Yan | HMDD2.0-Lan |
| --- | --- | --- | --- | --- | --- | --- | --- | --- | --- |
| 5 | 50 | 8 | 8 | 160 | 20 | 0.3 | 0.915846 | 0.930790 | 0.934183 |
| 5 | 50 | 8 | 8 | 160 | 20 | 0.4 | 0.925987 | 0.936140 | 0.936936 |
| 5 | 50 | 8 | 8 | 160 | 20 | 0.5 | 0.929584 | 0.934989 | 0.932589 |
| 5 | 50 | 8 | 8 | 160 | 20 | 0.6 | 0.926729 | 0.928831 | 0.926915 |
| 5 | 50 | 8 | 8 | 160 | 20 | 0.7 | 0.923498 | 0.924693 | 0.923448 |
| 5 | 50 | 8 | 8 | 160 | 20 | 0.9 | 0.910017 | 0.903306 | 0.897341 |
| 5 | 50 | 8 | 8 | 160 | 10 | 0.4 | 0.925415 | 0.937114 | 0.938065 |
| 5 | 50 | 8 | 8 | 160 | 30 | 0.4 | 0.925928 | 0.935301 | 0.935927 |
| 5 | 50 | 8 | 8 | 160 | 40 | 0.4 | 0.925806 | 0.934726 | 0.935274 |
| 5 | 50 | 8 | 8 | 160 | 100 | 0.4 | 0.923826 | 0.932881 | 0.931660 |
| 5 | 50 | 8 | 8 | 70 | 100 | 0.4 | 0.926139 | 0.937141 | 0.939319 |
| 5 | 50 | 8 | 8 | 110 | 10 | 0.4 | 0.926015 | 0.937384 | 0.938862 |
| 5 | 50 | 8 | 8 | 120 | 10 | 0.4 | 0.926190 | 0.937339 | 0.938759 |
| 5 | 50 | 8 | 8 | 140 | 10 | 0.4 | 0.925783 | 0.937050 | 0.938599 |
| 5 | 50 | 8 | 8 | 170 | 10 | 0.4 | 0.925292 | 0.936416 | 0.938171 |
| 5 | 50 | 8 | 8 | 200 | 10 | 0.4 | 0.925799 | 0.935887 | 0.937468 |
| 5 | 50 | 1 | 8 | 120 | 10 | 0.4 | 0.922906 | 0.922742 | 0.923901 |
| 5 | 50 | 7 | 8 | 120 | 10 | 0.4 | 0.925372 | 0.936013 | 0.936454 |
| 5 | 50 | 9 | 8 | 120 | 10 | 0.4 | 0.926085 | 0.936641 | 0.937012 |
| 5 | 50 | 20 | 8 | 120 | 10 | 0.4 | 0.925931 | 0.936018 | 0.936873 |
| 5 | 50 | 8 | 7 | 120 | 10 | 0.4 | 0.926134 | 0.935749 | 0.937923 |
| 5 | 50 | 8 | 9 | 120 | 10 | 0.4 | 0.925887 | 0.936273 | 0.936896 |
| 5 | 50 | 8 | 10 | 120 | 10 | 0.4 | 0.926061 | 0.936017 | 0.973528 |
| 5 | 70 | 8 | 8 | 120 | 10 | 0.4 | 0.926589 | 0.937609 | 0.938896 |
| 5 | 80 | 8 | 8 | 120 | 10 | 0.4 | 0.926542 | 0.937925 | 0.938673 |
| 5 | 90 | 8 | 8 | 120 | 10 | 0.4 | 0.926814 | 0.937770 | 0.938660 |
| 5 | 100 | 8 | 8 | 120 | 10 | 0.4 | 0.926580 | 0.937334 | 0.938699 |
| 5 | 150 | 8 | 8 | 120 | 10 | 0.4 | 0.926278 | 0.937809 | 0.938350 |
| 5 | 200 | 8 | 8 | 120 | 10 | 0.4 | 0.926028 | 0.937161 | 0.938315 |
| 1 | 80 | 8 | 8 | 120 | 10 | 0.4 | 0.926344 | 0.927945 | 0.924708 |
| 2 | 80 | 8 | 8 | 120 | 10 | 0.4 | 0.930036 | 0.936852 | 0.933843 |
| **3** | **80** | **8** | **8** | **120** | **10** | **0.4** | **0.930467** | **0.938365** | 0.936942 |
| 4 | 80 | 8 | 8 | 120 | 10 | 0.4 | 0.929429 | 0.938294 | 0.936785 |
| 6 | 80 | 8 | 8 | 120 | 10 | 0.4 | 0.924371 | 0.936193 | **0.937135** |
| 10 | 80 | 8 | 8 | 120 | 10 | 0.4 | 0.913990 | 0.929818 | 0.933815 |
| 20 | 80 | 8 | 8 | 120 | 10 | 0.4 | 0.898875 | 0.921386 | 0.926546 |
| 30 | 80 | 8 | 8 | 120 | 10 | 0.4 | 0.891777 | 0.916081 | 0.922867 |

UBiRW:

| r | l | HMDD2.0-You | HMDD2.0-Yan | HMDD2.0-Lan |
| --- | --- | --- | --- | --- |
| **1** | **1** | **0.919810** | **0.919108** | **0.919819** |
| 1 | 2 | 0.892940 | 0.899639 | 0.900559 |
| 1 | 4 | 0.837974 | 0.848705 | 0.870907 |
| 1 | 6 | 0.817866 | 0.828880 | 0.864296 |
| 2 | 1 | 0.825634 | 0.802424 | 0.733414 |
| 2 | 2 | 0.919587 | 0.919007 | 0.919511 |
| 2 | 4 | 0.838997 | 0.852886 | 0.877137 |
| 2 | 6 | 0.788270 | 0.820968 | 0.865616 |
| 4 | 1 | 0.778044 | 0.622210 | 0.582718 |
| 4 | 4 | 0.867542 | 0.860209 | 0.877720 |
| 6 | 6 | 0.705548 | 0.719381 | 0.740694 |
| 10 | 1 | 0.771557 | 0.605517 | 0.573517 |
| 10 | 10 | 0.353148 | 0.316627 | 0.303223 |

IMCMDA

| r | HMDD2.0-You | HMDD2.0-Yan | HMDD2.0-Lan |
| --- | --- | --- | --- |
| 1 | 0.827520 | 0.804595 | 0.721545 |
| 2 | 0.827769 | 0.804295 | 0.721655 |
| 3 | 0.828143 | 0.804574 | 0.721746 |
| 4 | 0.827972 | 0.804496 | 0.721737 |
| 5 | 0.828146 | 0.804477 | 0.721710 |
| **6** | **0.828388** | 0.804544 | **0.721822** |
| 7 | 0.828279 | 0.804349 | 0.721643 |
| 8 | 0.828029 | 0.804487 | 0.721551 |
| 9 | 0.828170 | 0.804535 | 0.721776 |
| 10 | 0.828004 | 0.804573 | 0.721692 |
| 20 | 0.827964 | 0.804572 | 0.721602 |
| 50 | 0.827502 | **0.804597** | 0.721713 |
| 100 | 0.827907 | 0.804286 | 0.721533 |

GRNMF

| K | r | p | k | *λ* | HMDD2.0-You | HMDD2.0-Yan | HMDD2.0-Lan |
| --- | --- | --- | --- | --- | --- | --- | --- |
| 2 | 0.5 | 5 | 60 | 0.5 | 0.886177 | 0.905231 | 0.899473 |
| 3 | 0.5 | 5 | 60 | 0.5 | 0.888279 | 0.905678 | 0.900074 |
| 4 | 0.5 | 5 | 60 | 0.5 | 0.887825 | 0.905023 | 0.898788 |
| 5 | 0.5 | 5 | 60 | 0.5 | 0.886517 | 0.903625 | 0.897654 |
| 10 | 0.5 | 5 | 60 | 0.5 | 0.878838 | 0.896388 | 0.890550 |
| 3 | 0.3 | 5 | 60 | 0.5 | 0.883525 | 0.902418 | 0.896281 |
| 3 | 0.7 | 5 | 60 | 0.5 | 0.891516 | 0.908292 | 0.903045 |
| 3 | 0.9 | 5 | 60 | 0.5 | 0.893333 | 0.909589 | 0.904628 |
| 3 | 0.9 | 5 | 70 | 0.5 | 0.893838 | 0.910162 | 0.905653 |
| 3 | 0.9 | 5 | 80 | 0.5 | 0.894729 | 0.910321 | 0.905901 |
| 3 | 0.9 | 5 | 90 | 0.5 | 0.894770 | 0.911033 | 0.906434 |
| 3 | 0.9 | 5 | 100 | 0.5 | 0.895173 | 0.911476 | 0.907356 |
| 3 | 0.9 | 5 | 100 | 0.25 | 0.890998 | 0.908492 | 0.903097 |
| 3 | 0.9 | 5 | 100 | 1 | 0.899720 | 0.914557 | 0.911751 |
| **3** | **0.9** | **5** | **100** | **2** | **0.903855** | **0.915257** | **0.915725** |
