# Supplementary material for: Predicting miRNA-Disease Association Based on Modularity Preserving Heterogeneous Network Embedding
Source: Front Cell Dev Biol. 2021 Jun 10;9:603758. doi: 10.3389/fcell.2021.603758 (PMC8223753; doi:10.3389/fcell.2021.603758)
Supplement: Supplementary file 2 [file Table_2.DOCX]

Table S2. Parameters of each method for four datasets

| MDN-NMTF | | | | | | | | | | | | | | | | | | | |
| --- | --- | --- | --- | --- | --- | --- | --- | --- | --- | --- | --- | --- | --- | --- | --- | --- | --- | --- | --- |
| *d_m_* | *d_d_* | | *λ_1_* | | *λ_2_* | | *λ_3_* | | *α_1_* | | | *α_2_* | | *β_1_* | *β_2_* | | *ω* | | *ε* |
| 200 | 200 | | 0.001 | | 5 | | 0.1 | | 0.2 | | | 0.8 | | 90 | 1.5 | | 160 | | 0.56 |
|  |  | |  | |  | |  | |  | | |  | |  |  | |  | |  |
| DNRLMF-MDA | | | | | | | | | | | | | | | | | | | |
| *c* | | *r* | | | | *λ_m_* | | | | *λ_d_* | | | *α* | | | *β* | | | *ε_1_* |
| 3 | | 80 | | | | 8 | | | | 8 | | | 120 | | | 10 | | | 0.4 |
|  | |  | | | |  | | | |  | | |  | | |  | | |  |
| IMCMDA | | | | | | | | | | | | | | | | | | | |
| *r* | | | | | | | | | | | | | | | | | | | |
| 6 | | | | | | | | | | | | | | | | | | | |
|  | | | | | | | | | | | | | | | | | | | |
| UBiRW | | | | | | | | | | | | | | | | | | | |
| *r* | | | | | | | | | | | *l* | | | | | | | | |
| 1 | | | | | | | | | | | 1 | | | | | | | | |
|  | | | | | | | | | | |  | | | | | | | | |
| GRNMF | | | | | | | | | | | | | | | | | | | |
| *K* | | | | *r* | | | | *p* | | | | | | *k* | | | | *λ* | |
| 3 | | | | 0.9 | | | | 5 | | | | | | 100 | | | | 2 | |
