# Supplementary material for: Predicting miRNA-Disease Association Based on Modularity Preserving Heterogeneous Network Embedding
Source: Front Cell Dev Biol. 2021 Jun 10;9:603758. doi: 10.3389/fcell.2021.603758 (PMC8223753; doi:10.3389/fcell.2021.603758)
Supplement: Supplementary file 3 [file Table_3.DOCX]

module 1

module 2 hsa-mir-103a-1;hsa-mir-103a-2;hsa-mir-103b-1;hsa-mir-103b-2;hsa-mir-107;hsa-mir-132;hsa-mir-186;hsa-mir-212;hsa-mir-216a;hsa-mir-216b;hsa-mir-217;hsa-mir-24-1;hsa-mir-24-2;hsa-mir-32;hsa-mir-325;hsa-mir-518c;hsa-mir-625;hsa-mir-630;

module 3

module 4

module 5

module 6 hsa-mir-122;hsa-mir-15a;hsa-mir-15b;hsa-mir-16-1;hsa-mir-16-2;hsa-mir-203;hsa-mir-210;hsa-mir-223;hsa-mir-24-1;hsa-mir-24-2;hsa-mir-297;hsa-mir-320a;hsa-mir-483;hsa-mir-486;hsa-mir-629;hsa-mir-99a;

module 7

module 8

module 9 hsa-mir-1249;hsa-mir-23b;hsa-mir-342;hsa-mir-381;hsa-mir-432;hsa-mir-493;hsa-mir-516a-1;hsa-mir-516a-2;hsa-mir-516b-1;hsa-mir-516b-2;hsa-mir-539;hsa-mir-550a-1;hsa-mir-550a-2;hsa-mir-664;hsa-mir-671;

module 10 hsa-mir-100;hsa-mir-10a;hsa-mir-1258;hsa-mir-125a;hsa-mir-135a-1;hsa-mir-135a-2;hsa-mir-135b;hsa-mir-151b;hsa-mir-193a;hsa-mir-194-1;hsa-mir-194-2;hsa-mir-196b;hsa-mir-199a-1;hsa-mir-199a-2;hsa-mir-202;hsa-mir-203;hsa-mir-23a;hsa-mir-23b;hsa-mir-330;hsa-mir-34c;hsa-mir-365a;hsa-mir-365b;hsa-mir-374a;hsa-mir-424;hsa-mir-449a;hsa-mir-449b;hsa-mir-451b;hsa-mir-485;hsa-mir-504;hsa-mir-509-3;hsa-mir-520a;hsa-mir-520b;hsa-mir-520c;hsa-mir-520d;hsa-mir-520e;hsa-mir-520f;hsa-mir-520g;hsa-mir-520h;hsa-mir-574;hsa-mir-708;hsa-mir-720;hsa-mir-873;hsa-mir-99a;hsa-mir-99b;

module 11

module 12

module 13 hsa-mir-106a;hsa-mir-106b;hsa-mir-10a;hsa-mir-10b;hsa-mir-124-1;hsa-mir-124-2;hsa-mir-124-3;hsa-mir-127;hsa-mir-128;hsa-mir-128-1;hsa-mir-128-2;hsa-mir-135a-1;hsa-mir-135a-2;hsa-mir-137;hsa-mir-138-1;hsa-mir-138-2;hsa-mir-139;hsa-mir-145;hsa-mir-146b;hsa-mir-149;hsa-mir-152;hsa-mir-153-2;hsa-mir-181d;hsa-mir-185;hsa-mir-18b;hsa-mir-190a;hsa-mir-190b;hsa-mir-195;hsa-mir-196b;hsa-mir-204;hsa-mir-20b;hsa-mir-210;hsa-mir-211;hsa-mir-215;hsa-mir-218-1;hsa-mir-218-2;hsa-mir-221;hsa-mir-222;hsa-mir-224;hsa-mir-26b;hsa-mir-27b;hsa-mir-302a;hsa-mir-302b;hsa-mir-302c;hsa-mir-302d;hsa-mir-30e;hsa-mir-31;hsa-mir-326;hsa-mir-335;hsa-mir-339;hsa-mir-340;hsa-mir-34a;hsa-mir-367;hsa-mir-370;hsa-mir-371a;hsa-mir-373;hsa-mir-383;hsa-mir-410;hsa-mir-451a;hsa-mir-483;hsa-mir-487b;hsa-mir-497;hsa-mir-542;hsa-mir-544a;hsa-mir-544b;hsa-mir-628;hsa-mir-708;hsa-mir-885;hsa-mir-9-1;hsa-mir-9-2;hsa-mir-9-3;hsa-mir-92b;hsa-mir-93;hsa-mir-99a;

module 14 hsa-let-7a;hsa-let-7a-1;hsa-let-7a-2;hsa-let-7a-3;hsa-let-7b;hsa-let-7c;hsa-let-7d;hsa-let-7e;hsa-let-7f-1;hsa-let-7f-2;hsa-let-7g;hsa-let-7i;hsa-mir-100;hsa-mir-101-1;hsa-mir-101-2;hsa-mir-103a-1;hsa-mir-103a-2;hsa-mir-103b-1;hsa-mir-103b-2;hsa-mir-105-1;hsa-mir-105-2;hsa-mir-106a;hsa-mir-106b;hsa-mir-107;hsa-mir-10a;hsa-mir-10b;hsa-mir-1179;hsa-mir-1180;hsa-mir-1181;hsa-mir-1183;hsa-mir-1184-1;hsa-mir-1202;hsa-mir-1207;hsa-mir-122;hsa-mir-1224;hsa-mir-1227;hsa-mir-1229;hsa-mir-1231;hsa-mir-1233-1;hsa-mir-1233-2;hsa-mir-1234;hsa-mir-1236;hsa-mir-124-1;hsa-mir-124-2;hsa-mir-124-3;hsa-mir-1246;hsa-mir-1247;hsa-mir-1249;hsa-mir-124a-2;hsa-mir-124a-3;hsa-mir-1254-1;hsa-mir-1256;hsa-mir-1258;hsa-mir-125a;hsa-mir-125b-1;hsa-mir-125b-2;hsa-mir-126;hsa-mir-1260a;hsa-mir-1266;hsa-mir-127;hsa-mir-1271;hsa-mir-1273a;hsa-mir-1273c;hsa-mir-1275;hsa-mir-128;hsa-mir-128-1;hsa-mir-128-2;hsa-mir-1280;hsa-mir-1285-1;hsa-mir-1285-2;hsa-mir-1286;hsa-mir-129-1;hsa-mir-129-2;hsa-mir-1290;hsa-mir-1293;hsa-mir-1296;hsa-mir-1297;hsa-mir-1299;hsa-mir-1301;hsa-mir-1303;hsa-mir-1305;hsa-mir-130a;hsa-mir-130b;hsa-mir-132;hsa-mir-1322;hsa-mir-133b;hsa-mir-134;hsa-mir-135a-1;hsa-mir-135a-2;hsa-mir-135b;hsa-mir-136;hsa-mir-137;hsa-mir-138-1;hsa-mir-138-2;hsa-mir-139;hsa-mir-140;hsa-mir-141;hsa-mir-142;hsa-mir-143;hsa-mir-144;hsa-mir-145;hsa-mir-146b;hsa-mir-1471;hsa-mir-147a;hsa-mir-147b;hsa-mir-148a;hsa-mir-148b;hsa-mir-149;hsa-mir-150;hsa-mir-151;hsa-mir-151a;hsa-mir-151b;hsa-mir-152;hsa-mir-153-1;hsa-mir-153-2;hsa-mir-154;hsa-mir-15a;hsa-mir-15b;hsa-mir-16-1;hsa-mir-16-2;hsa-mir-17;hsa-mir-181a-1;hsa-mir-181a-2;hsa-mir-181b-1;hsa-mir-181b-2;hsa-mir-181c;hsa-mir-181d;hsa-mir-182;hsa-mir-1827;hsa-mir-183;hsa-mir-184;hsa-mir-185;hsa-mir-186;hsa-mir-187;hsa-mir-188;hsa-mir-189;hsa-mir-18a;hsa-mir-18b;hsa-mir-1909;hsa-mir-190a;hsa-mir-190b;hsa-mir-191;hsa-mir-1915;hsa-mir-192;hsa-mir-192-2;hsa-mir-193a;hsa-mir-193b;hsa-mir-194-1;hsa-mir-194-2;hsa-mir-195;hsa-mir-196a-1;hsa-mir-196a-2;hsa-mir-196b;hsa-mir-197;hsa-mir-1972-1;hsa-mir-198;hsa-mir-199a-1;hsa-mir-199a-2;hsa-mir-199b;hsa-mir-19a;hsa-mir-19b-1;hsa-mir-19b-2;hsa-mir-200;hsa-mir-200a;hsa-mir-200b;hsa-mir-200c;hsa-mir-202;hsa-mir-203;hsa-mir-204;hsa-mir-205;hsa-mir-206;hsa-mir-208a;hsa-mir-208b;hsa-mir-20a;hsa-mir-20b;hsa-mir-210;hsa-mir-211;hsa-mir-2110;hsa-mir-212;hsa-mir-214;hsa-mir-215;hsa-mir-216a;hsa-mir-216b;hsa-mir-217;hsa-mir-218-1;hsa-mir-218-2;hsa-mir-219-1;hsa-mir-219-2;hsa-mir-22;hsa-mir-221;hsa-mir-222;hsa-mir-223;hsa-mir-224;hsa-mir-23a;hsa-mir-23b;hsa-mir-24-1;hsa-mir-24-2;hsa-mir-25;hsa-mir-26;hsa-mir-26a-1;hsa-mir-26a-2;hsa-mir-26b;hsa-mir-27a;hsa-mir-27b;hsa-mir-28;hsa-mir-2861;hsa-mir-296;hsa-mir-297;hsa-mir-298;hsa-mir-299;hsa-mir-29b;hsa-mir-300;hsa-mir-301a;hsa-mir-301b;hsa-mir-302a;hsa-mir-302b;hsa-mir-302c;hsa-mir-302d;hsa-mir-302e;hsa-mir-302f;hsa-mir-30a;hsa-mir-30b;hsa-mir-30c-1;hsa-mir-30c-2;hsa-mir-30d;hsa-mir-30e;hsa-mir-31;hsa-mir-3148;hsa-mir-3151;hsa-mir-3163;hsa-mir-3179-1;hsa-mir-3179-2;hsa-mir-3179-3;hsa-mir-3196;hsa-mir-32;hsa-mir-320a;hsa-mir-320b-1;hsa-mir-320b-2;hsa-mir-320c-1;hsa-mir-320c-2;hsa-mir-320d-1;hsa-mir-320d-2;hsa-mir-320e;hsa-mir-323a;hsa-mir-323b;hsa-mir-324;hsa-mir-325;hsa-mir-326;hsa-mir-328;hsa-mir-329-1;hsa-mir-329-2;hsa-mir-330;hsa-mir-331;hsa-mir-335;hsa-mir-337;hsa-mir-338;hsa-mir-339;hsa-mir-33a;hsa-mir-33b;hsa-mir-340;hsa-mir-342;hsa-mir-345;hsa-mir-346;hsa-mir-34a;hsa-mir-34b;hsa-mir-34c;hsa-mir-361;hsa-mir-362;hsa-mir-363;hsa-mir-365a;hsa-mir-365b;hsa-mir-367;hsa-mir-369;hsa-mir-370;hsa-mir-371;hsa-mir-371a;hsa-mir-372;hsa-mir-373;hsa-mir-374a;hsa-mir-375;hsa-mir-376a-1;hsa-mir-376a-2;hsa-mir-376b;hsa-mir-376c;hsa-mir-377;hsa-mir-378a;hsa-mir-379;hsa-mir-381;hsa-mir-382;hsa-mir-383;hsa-mir-384;hsa-mir-3940;hsa-mir-409;hsa-mir-410;hsa-mir-411;hsa-mir-421;hsa-mir-422a;hsa-mir-423;hsa-mir-424;hsa-mir-425;hsa-mir-429;hsa-mir-431;hsa-mir-432;hsa-mir-433;hsa-mir-448;hsa-mir-449a;hsa-mir-449b;hsa-mir-450a-1;hsa-mir-450a-2;hsa-mir-450b;hsa-mir-451;hsa-mir-451a;hsa-mir-451b;hsa-mir-452;hsa-mir-454;hsa-mir-455;hsa-mir-4792;hsa-mir-483;hsa-mir-484;hsa-mir-485;hsa-mir-486;hsa-mir-487a;hsa-mir-487b;hsa-mir-488;hsa-mir-489;hsa-mir-490;hsa-mir-491;hsa-mir-492;hsa-mir-493;hsa-mir-494;hsa-mir-495;hsa-mir-497;hsa-mir-498;hsa-mir-499a;hsa-mir-499b;hsa-mir-500a;hsa-mir-500b;hsa-mir-501;hsa-mir-502;hsa-mir-503;hsa-mir-504;hsa-mir-505;hsa-mir-506;hsa-mir-507;hsa-mir-508;hsa-mir-509;hsa-mir-509-1;hsa-mir-509-2;hsa-mir-509-3;hsa-mir-510;hsa-mir-511-1;hsa-mir-511-2;hsa-mir-512-1;hsa-mir-512-2;hsa-mir-513a-1;hsa-mir-513a-2;hsa-mir-513b;hsa-mir-513c;hsa-mir-514a-1;hsa-mir-514a-2;hsa-mir-514a-3;hsa-mir-515-1;hsa-mir-515-2;hsa-mir-516a-1;hsa-mir-516a-2;hsa-mir-516b-1;hsa-mir-516b-2;hsa-mir-517a;hsa-mir-517c;hsa-mir-518a-1;hsa-mir-518a-2;hsa-mir-518b;hsa-mir-518c;hsa-mir-518e;hsa-mir-519a-1;hsa-mir-519a-2;hsa-mir-519b;hsa-mir-519c;hsa-mir-519d;hsa-mir-519e;hsa-mir-520a;hsa-mir-520b;hsa-mir-520c;hsa-mir-520d;hsa-mir-520e;hsa-mir-520f;hsa-mir-520g;hsa-mir-520h;hsa-mir-521-1;hsa-mir-521-2;hsa-mir-522;hsa-mir-523;hsa-mir-525;hsa-mir-526a-1;hsa-mir-526a-2;hsa-mir-526b;hsa-mir-527;hsa-mir-532;hsa-mir-539;hsa-mir-542;hsa-mir-544a;hsa-mir-544b;hsa-mir-545;hsa-mir-548a-1;hsa-mir-548a-2;hsa-mir-548a-3;hsa-mir-548b;hsa-mir-548c;hsa-mir-548d-1;hsa-mir-548d-2;hsa-mir-550a-1;hsa-mir-550a-2;hsa-mir-550a-3;hsa-mir-550b-1;hsa-mir-550b-2;hsa-mir-552;hsa-mir-557;hsa-mir-561;hsa-mir-564;hsa-mir-566;hsa-mir-567;hsa-mir-569;hsa-mir-571;hsa-mir-572;hsa-mir-574;hsa-mir-575;hsa-mir-582;hsa-mir-583;hsa-mir-584;hsa-mir-589;hsa-mir-590;hsa-mir-592;hsa-mir-593;hsa-mir-596;hsa-mir-598;hsa-mir-599;hsa-mir-600;hsa-mir-601;hsa-mir-602;hsa-mir-603;hsa-mir-608;hsa-mir-611;hsa-mir-612;hsa-mir-614;hsa-mir-615;hsa-mir-616;hsa-mir-617;hsa-mir-619;hsa-mir-621;hsa-mir-622;hsa-mir-624;hsa-mir-625;hsa-mir-627;hsa-mir-628;hsa-mir-629;hsa-mir-630;hsa-mir-632;hsa-mir-633;hsa-mir-635;hsa-mir-636;hsa-mir-637;hsa-mir-638;hsa-mir-640;hsa-mir-642a;hsa-mir-642b;hsa-mir-646;hsa-mir-647;hsa-mir-648;hsa-mir-650;hsa-mir-652;hsa-mir-654;hsa-mir-655;hsa-mir-657;hsa-mir-658;hsa-mir-659;hsa-mir-660;hsa-mir-661;hsa-mir-662;hsa-mir-663;hsa-mir-663a;hsa-mir-663b;hsa-mir-664;hsa-mir-668;hsa-mir-671;hsa-mir-675;hsa-mir-7-1;hsa-mir-7-2;hsa-mir-7-3;hsa-mir-708;hsa-mir-711;hsa-mir-720;hsa-mir-744;hsa-mir-758;hsa-mir-760;hsa-mir-765;hsa-mir-766;hsa-mir-767;hsa-mir-769;hsa-mir-873;hsa-mir-874;hsa-mir-885;hsa-mir-9-1;hsa-mir-9-2;hsa-mir-9-3;hsa-mir-92;hsa-mir-922;hsa-mir-92a-1;hsa-mir-92a-2;hsa-mir-92b;hsa-mir-93;hsa-mir-933;hsa-mir-935;hsa-mir-941-1;hsa-mir-941-3;hsa-mir-941-4;hsa-mir-942;hsa-mir-944;hsa-mir-95;hsa-mir-96;hsa-mir-98;hsa-mir-99a;hsa-mir-99b;

module 15

module 16 hsa-mir-122;hsa-mir-1302-1;hsa-mir-1302-2;hsa-mir-1302-3;hsa-mir-1302-4;hsa-mir-1302-5;hsa-mir-1302-6;hsa-mir-1302-7;hsa-mir-1302-8;hsa-mir-374b;hsa-mir-433;hsa-mir-508;hsa-mir-509-1;hsa-mir-509-2;hsa-mir-509-3;hsa-mir-513a-1;hsa-mir-513a-2;hsa-mir-513b;hsa-mir-513c;hsa-mir-514a-1;hsa-mir-514a-2;hsa-mir-514a-3;hsa-mir-516a-1;hsa-mir-516a-2;hsa-mir-516b-1;hsa-mir-516b-2;

module 17 hsa-mir-101-1;hsa-mir-101-2;hsa-mir-129-1;hsa-mir-129-2;hsa-mir-138-2;hsa-mir-139;hsa-mir-146a;hsa-mir-148b;hsa-mir-184;hsa-mir-18b;hsa-mir-20b;hsa-mir-223;hsa-mir-345;hsa-mir-363;hsa-mir-486;hsa-mir-499a;hsa-mir-518b;hsa-mir-539;hsa-mir-550a-1;hsa-mir-550a-2;hsa-mir-629;hsa-mir-708;hsa-mir-92b;

module 18

module 19

module 20 hsa-let-7a;hsa-mir-1254-1;hsa-mir-1258;hsa-mir-135a-1;hsa-mir-135a-2;hsa-mir-136;hsa-mir-1471;hsa-mir-151b;hsa-mir-187;hsa-mir-202;hsa-mir-320e;hsa-mir-325;hsa-mir-340;hsa-mir-361;hsa-mir-365a;hsa-mir-365b;hsa-mir-374a;hsa-mir-377;hsa-mir-3940;hsa-mir-421;hsa-mir-424;hsa-mir-450a-1;hsa-mir-450a-2;hsa-mir-450b;hsa-mir-485;hsa-mir-504;hsa-mir-507;hsa-mir-512-1;hsa-mir-515-1;hsa-mir-515-2;hsa-mir-519a-1;hsa-mir-519a-2;hsa-mir-519b;hsa-mir-519c;hsa-mir-519d;hsa-mir-519e;hsa-mir-520a;hsa-mir-520b;hsa-mir-520c;hsa-mir-520d;hsa-mir-520e;hsa-mir-520f;hsa-mir-520g;hsa-mir-520h;hsa-mir-523;hsa-mir-525;hsa-mir-526a-1;hsa-mir-526a-2;hsa-mir-526b;hsa-mir-548a-1;hsa-mir-548a-2;hsa-mir-548a-3;hsa-mir-574;hsa-mir-650;hsa-mir-661;hsa-mir-708;hsa-mir-720;hsa-mir-873;hsa-mir-874;

module 21 hsa-let-7a-1;hsa-mir-122;hsa-mir-125b-1;hsa-mir-125b-2;hsa-mir-143;hsa-mir-145;hsa-mir-150;hsa-mir-15a;hsa-mir-15b;hsa-mir-16-1;hsa-mir-16-2;hsa-mir-17;hsa-mir-181a-1;hsa-mir-181a-2;hsa-mir-18a;hsa-mir-20a;hsa-mir-21;hsa-mir-221;hsa-mir-222;hsa-mir-297;hsa-mir-34a;hsa-mir-561;hsa-mir-636;hsa-mir-671;hsa-mir-720;hsa-mir-92a-1;

module 22 hsa-mir-124-1;hsa-mir-124-2;hsa-mir-124-3;hsa-mir-135b;hsa-mir-181b-1;hsa-mir-181b-2;hsa-mir-181c;hsa-mir-192;hsa-mir-214;hsa-mir-24-1;hsa-mir-24-2;hsa-mir-34c;hsa-mir-361;hsa-mir-376a-1;hsa-mir-376a-2;hsa-mir-491;hsa-mir-497;hsa-mir-513a-1;hsa-mir-513a-2;hsa-mir-513b;hsa-mir-513c;hsa-mir-571;hsa-mir-652;

module 23 hsa-mir-1224;hsa-mir-1227;hsa-mir-1229;hsa-mir-1233-1;hsa-mir-1233-2;hsa-mir-124a-2;hsa-mir-124a-3;hsa-mir-1256;hsa-mir-1280;hsa-mir-1285-1;hsa-mir-1285-2;hsa-mir-1296;hsa-mir-2861;hsa-mir-300;hsa-mir-3196;hsa-mir-378a;hsa-mir-378b;hsa-mir-378c;hsa-mir-378d-1;hsa-mir-378d-2;hsa-mir-378e;hsa-mir-378f;hsa-mir-378g;hsa-mir-378h;hsa-mir-378i;hsa-mir-452;hsa-mir-455;hsa-mir-517a;hsa-mir-521-1;hsa-mir-521-2;hsa-mir-572;hsa-mir-574;hsa-mir-616;hsa-mir-642a;hsa-mir-642b;hsa-mir-647;hsa-mir-93;hsa-mir-944;

module 24 hsa-mir-101-1;hsa-mir-101-2;hsa-mir-106a;hsa-mir-106b;hsa-mir-125a;hsa-mir-125b-1;hsa-mir-125b-2;hsa-mir-142;hsa-mir-155;hsa-mir-15a;hsa-mir-16-1;hsa-mir-16-2;hsa-mir-17;hsa-mir-181a-1;hsa-mir-181a-2;hsa-mir-18a;hsa-mir-18b;hsa-mir-19a;hsa-mir-19b-1;hsa-mir-19b-2;hsa-mir-20a;hsa-mir-20b;hsa-mir-31;hsa-mir-34a;hsa-mir-711;hsa-mir-92a-1;hsa-mir-92a-2;hsa-mir-93;

module 25

module 26 hsa-mir-10a;hsa-mir-135a-1;hsa-mir-135a-2;hsa-mir-153-2;hsa-mir-224;hsa-mir-323a;hsa-mir-338;hsa-mir-33b;hsa-mir-361;hsa-mir-376a-1;hsa-mir-376a-2;hsa-mir-376c;hsa-mir-379;hsa-mir-383;hsa-mir-452;hsa-mir-483;hsa-mir-494;hsa-mir-495;hsa-mir-504;hsa-mir-512-2;hsa-mir-539;hsa-mir-548d-1;hsa-mir-548d-2;hsa-mir-92b;hsa-mir-935;

module 27 hsa-mir-370;

module 28

module 29 hsa-mir-10a;hsa-mir-127;hsa-mir-140;hsa-mir-154;hsa-mir-181c;hsa-mir-181d;hsa-mir-219-1;hsa-mir-219-2;hsa-mir-224;hsa-mir-299;hsa-mir-3151;hsa-mir-323a;hsa-mir-337;hsa-mir-338;hsa-mir-33b;hsa-mir-370;hsa-mir-382;hsa-mir-590;hsa-mir-628;

module 30 hsa-mir-1260a;hsa-mir-128-1;hsa-mir-128-2;hsa-mir-1305;hsa-mir-137;hsa-mir-146b;hsa-mir-149;hsa-mir-153-1;hsa-mir-153-2;hsa-mir-200;hsa-mir-20b;hsa-mir-27b;hsa-mir-302d;hsa-mir-3163;hsa-mir-326;hsa-mir-329-1;hsa-mir-329-2;hsa-mir-338;hsa-mir-376a-1;hsa-mir-376a-2;hsa-mir-383;hsa-mir-422a;hsa-mir-491;hsa-mir-572;hsa-mir-599;hsa-mir-614;hsa-mir-648;hsa-mir-873;hsa-mir-885;hsa-mir-96;

module 31

module 32 hsa-let-7a-1;hsa-let-7a-2;hsa-let-7b;hsa-let-7c;hsa-let-7d;hsa-let-7f-1;hsa-let-7f-2;hsa-let-7g;hsa-let-7i;hsa-mir-101-1;hsa-mir-101-2;hsa-mir-151;hsa-mir-195;hsa-mir-216b;hsa-mir-26a-1;hsa-mir-26a-2;hsa-mir-26b;hsa-mir-30b;hsa-mir-342;hsa-mir-98;

module 33 hsa-mir-130a;hsa-mir-147a;hsa-mir-151a;hsa-mir-187;hsa-mir-206;hsa-mir-22;hsa-mir-23b;hsa-mir-335;hsa-mir-342;hsa-mir-374a;hsa-mir-376c;hsa-mir-432;hsa-mir-433;hsa-mir-493;hsa-mir-516a-1;hsa-mir-516a-2;hsa-mir-516b-1;hsa-mir-516b-2;hsa-mir-550a-1;hsa-mir-550a-2;hsa-mir-601;hsa-mir-640;hsa-mir-658;hsa-mir-664;hsa-mir-671;hsa-mir-922;

module 34

module 35 hsa-mir-203;hsa-mir-99a;

module 36 hsa-mir-10a;hsa-mir-10b;hsa-mir-128;hsa-mir-128-1;hsa-mir-128-2;hsa-mir-130b;hsa-mir-138-1;hsa-mir-138-2;hsa-mir-149;hsa-mir-153-2;hsa-mir-182;hsa-mir-18b;hsa-mir-204;hsa-mir-20b;hsa-mir-211;hsa-mir-215;hsa-mir-218-1;hsa-mir-26b;hsa-mir-28;hsa-mir-302a;hsa-mir-302b;hsa-mir-302c;hsa-mir-302d;hsa-mir-302e;hsa-mir-302f;hsa-mir-30e;hsa-mir-325;hsa-mir-339;hsa-mir-367;hsa-mir-371a;hsa-mir-372;hsa-mir-373;hsa-mir-383;hsa-mir-423;hsa-mir-455;hsa-mir-488;hsa-mir-544a;hsa-mir-544b;hsa-mir-92b;hsa-mir-96;

module 37

module 38

module 39 hsa-mir-129-1;hsa-mir-129-2;hsa-mir-146b;hsa-mir-148b;hsa-mir-149;hsa-mir-197;hsa-mir-210;hsa-mir-212;hsa-mir-214;hsa-mir-221;hsa-mir-222;hsa-mir-224;hsa-mir-25;hsa-mir-338;hsa-mir-340;hsa-mir-361;hsa-mir-363;hsa-mir-370;hsa-mir-378a;hsa-mir-423;hsa-mir-489;hsa-mir-492;hsa-mir-499b;hsa-mir-515-1;hsa-mir-515-2;hsa-mir-526a-1;hsa-mir-526a-2;hsa-mir-661;hsa-mir-874;hsa-mir-92a-2;hsa-mir-92b;hsa-mir-95;

module 40 hsa-mir-103b-1;hsa-mir-103b-2;hsa-mir-186;hsa-mir-194-1;hsa-mir-194-2;hsa-mir-216a;hsa-mir-216b;hsa-mir-217;hsa-mir-32;hsa-mir-625;

module 41

module 42 hsa-mir-130a;hsa-mir-130b;hsa-mir-148a;hsa-mir-151a;hsa-mir-15a;hsa-mir-15b;hsa-mir-16-1;hsa-mir-16-2;hsa-mir-181a-1;hsa-mir-181a-2;hsa-mir-187;hsa-mir-192;hsa-mir-194-1;hsa-mir-194-2;hsa-mir-195;hsa-mir-203;hsa-mir-210;hsa-mir-215;hsa-mir-22;hsa-mir-221;hsa-mir-222;hsa-mir-223;hsa-mir-23b;hsa-mir-30b;hsa-mir-335;hsa-mir-342;hsa-mir-376b;hsa-mir-483;hsa-mir-494;hsa-mir-561;hsa-mir-625;hsa-mir-636;hsa-mir-640;hsa-mir-650;hsa-mir-99b;

module 43 hsa-mir-100;hsa-mir-103a-1;hsa-mir-103a-2;hsa-mir-107;hsa-mir-125a;hsa-mir-126;hsa-mir-130a;hsa-mir-132;hsa-mir-136;hsa-mir-140;hsa-mir-143;hsa-mir-145;hsa-mir-146b;hsa-mir-148a;hsa-mir-151a;hsa-mir-154;hsa-mir-195;hsa-mir-199a-1;hsa-mir-199a-2;hsa-mir-199b;hsa-mir-206;hsa-mir-210;hsa-mir-214;hsa-mir-22;hsa-mir-221;hsa-mir-222;hsa-mir-223;hsa-mir-28;hsa-mir-299;hsa-mir-30a;hsa-mir-320a;hsa-mir-335;hsa-mir-34b;hsa-mir-34c;hsa-mir-362;hsa-mir-376c;hsa-mir-379;hsa-mir-381;hsa-mir-432;hsa-mir-452;hsa-mir-487b;hsa-mir-495;hsa-mir-501;hsa-mir-650;hsa-mir-99b;

module 44 hsa-mir-106a;hsa-mir-10a;hsa-mir-1260a;hsa-mir-128-1;hsa-mir-128-2;hsa-mir-1305;hsa-mir-130a;hsa-mir-134;hsa-mir-137;hsa-mir-138-1;hsa-mir-138-2;hsa-mir-139;hsa-mir-149;hsa-mir-153-1;hsa-mir-153-2;hsa-mir-181d;hsa-mir-184;hsa-mir-197;hsa-mir-200;hsa-mir-208a;hsa-mir-208b;hsa-mir-211;hsa-mir-218-2;hsa-mir-302a;hsa-mir-302b;hsa-mir-302c;hsa-mir-302d;hsa-mir-302f;hsa-mir-3163;hsa-mir-32;hsa-mir-323a;hsa-mir-323b;hsa-mir-326;hsa-mir-328;hsa-mir-329-1;hsa-mir-329-2;hsa-mir-363;hsa-mir-367;hsa-mir-376a-1;hsa-mir-376a-2;hsa-mir-381;hsa-mir-383;hsa-mir-425;hsa-mir-452;hsa-mir-455;hsa-mir-491;hsa-mir-504;hsa-mir-539;hsa-mir-548b;hsa-mir-873;hsa-mir-885;hsa-mir-95;

module 45

module 46 hsa-mir-106b;hsa-mir-143;hsa-mir-144;hsa-mir-145;hsa-mir-16-2;hsa-mir-188;hsa-mir-205;hsa-mir-20b;hsa-mir-21;hsa-mir-24-2;hsa-mir-31;hsa-mir-34a;hsa-mir-384;hsa-mir-422a;hsa-mir-486;hsa-mir-487a;hsa-mir-490;hsa-mir-598;

module 47 hsa-mir-1179;hsa-mir-1293;hsa-mir-130b;hsa-mir-144;hsa-mir-147a;hsa-mir-147b;hsa-mir-181b-1;hsa-mir-181b-2;hsa-mir-19b-2;hsa-mir-223;hsa-mir-24-1;hsa-mir-302a;hsa-mir-3179-1;hsa-mir-3179-2;hsa-mir-3179-3;hsa-mir-329-1;hsa-mir-329-2;hsa-mir-342;hsa-mir-421;hsa-mir-424;hsa-mir-431;hsa-mir-432;hsa-mir-451a;hsa-mir-486;hsa-mir-499a;hsa-mir-505;hsa-mir-519a-1;hsa-mir-520d;hsa-mir-539;hsa-mir-548a-1;hsa-mir-548a-2;hsa-mir-548a-3;hsa-mir-548b;hsa-mir-550a-1;hsa-mir-550a-2;hsa-mir-550a-3;hsa-mir-550b-1;hsa-mir-550b-2;hsa-mir-640;hsa-mir-664;hsa-mir-744;hsa-mir-942;

module 48

module 49

module 50 hsa-mir-186;hsa-mir-211;hsa-mir-215;hsa-mir-216a;hsa-mir-216b;hsa-mir-297;hsa-mir-330;hsa-mir-339;hsa-mir-365a;hsa-mir-365b;hsa-mir-584;hsa-mir-646;hsa-mir-95;

module 51 hsa-mir-720;

module 52 hsa-mir-100;hsa-mir-106b;hsa-mir-132;hsa-mir-137;hsa-mir-140;hsa-mir-146b;hsa-mir-149;hsa-mir-15b;hsa-mir-193a;hsa-mir-193b;hsa-mir-196a-1;hsa-mir-196a-2;hsa-mir-203;hsa-mir-210;hsa-mir-211;hsa-mir-212;hsa-mir-218-2;hsa-mir-27b;hsa-mir-31;hsa-mir-339;hsa-mir-375;hsa-mir-376b;hsa-mir-410;hsa-mir-484;hsa-mir-491;hsa-mir-592;hsa-mir-596;hsa-mir-675;hsa-mir-7-1;hsa-mir-7-2;hsa-mir-7-3;hsa-mir-767;hsa-mir-874;hsa-mir-885;hsa-mir-9-3;hsa-mir-93;hsa-mir-99a;

module 53 hsa-mir-508;hsa-mir-514a-1;hsa-mir-514a-2;hsa-mir-514a-3;

module 54 hsa-mir-100;hsa-mir-203;hsa-mir-99a;

module 55 hsa-mir-106a;hsa-mir-106b;hsa-mir-132;hsa-mir-15b;hsa-mir-181b-1;hsa-mir-181b-2;hsa-mir-181c;hsa-mir-181d;hsa-mir-196b;hsa-mir-212;hsa-mir-219-1;hsa-mir-219-2;hsa-mir-23a;hsa-mir-23b;hsa-mir-24-1;hsa-mir-24-2;hsa-mir-25;hsa-mir-27a;hsa-mir-27b;hsa-mir-338;hsa-mir-489;hsa-mir-874;

module 56

module 57 hsa-mir-106a;hsa-mir-106b;hsa-mir-132;hsa-mir-15b;hsa-mir-181b-1;hsa-mir-181b-2;hsa-mir-181c;hsa-mir-181d;hsa-mir-196a-1;hsa-mir-196b;hsa-mir-197;hsa-mir-212;hsa-mir-219-2;hsa-mir-23a;hsa-mir-23b;hsa-mir-24-1;hsa-mir-24-2;hsa-mir-25;hsa-mir-27a;hsa-mir-30a;hsa-mir-431;hsa-mir-548a-1;hsa-mir-548a-2;hsa-mir-548a-3;

module 58 hsa-let-7a;hsa-mir-103b-1;hsa-mir-103b-2;hsa-mir-10a;hsa-mir-1224;hsa-mir-1245a;hsa-mir-1245b;hsa-mir-1258;hsa-mir-125b;hsa-mir-128-1;hsa-mir-128-2;hsa-mir-129-1;hsa-mir-129-2;hsa-mir-1290;hsa-mir-1323;hsa-mir-135a-1;hsa-mir-135a-2;hsa-mir-135b;hsa-mir-139;hsa-mir-1469;hsa-mir-1471;hsa-mir-147a;hsa-mir-148b;hsa-mir-149;hsa-mir-151b;hsa-mir-152;hsa-mir-153-2;hsa-mir-181;hsa-mir-187;hsa-mir-189;hsa-mir-18b;hsa-mir-191;hsa-mir-1915;hsa-mir-194-1;hsa-mir-194-2;hsa-mir-197;hsa-mir-200;hsa-mir-202;hsa-mir-204;hsa-mir-20b;hsa-mir-215;hsa-mir-218-1;hsa-mir-218-2;hsa-mir-2355;hsa-mir-26b;hsa-mir-298;hsa-mir-299;hsa-mir-301b;hsa-mir-302a;hsa-mir-302b;hsa-mir-302c;hsa-mir-302d;hsa-mir-3130-1;hsa-mir-3130-2;hsa-mir-3179-1;hsa-mir-3179-2;hsa-mir-3186;hsa-mir-320b-1;hsa-mir-320b-2;hsa-mir-320c-1;hsa-mir-320c-2;hsa-mir-320d-1;hsa-mir-320d-2;hsa-mir-320e;hsa-mir-323b;hsa-mir-324;hsa-mir-326;hsa-mir-328;hsa-mir-338;hsa-mir-339;hsa-mir-340;hsa-mir-345;hsa-mir-365a;hsa-mir-367;hsa-mir-374a;hsa-mir-376c;hsa-mir-383;hsa-mir-409;hsa-mir-411;hsa-mir-423;hsa-mir-425;hsa-mir-4257;hsa-mir-4306;hsa-mir-450a-1;hsa-mir-450a-2;hsa-mir-450b;hsa-mir-452;hsa-mir-488;hsa-mir-493;hsa-mir-495;hsa-mir-498;hsa-mir-505;hsa-mir-506;hsa-mir-510;hsa-mir-513a-1;hsa-mir-513a-2;hsa-mir-513b;hsa-mir-513c;hsa-mir-515-1;hsa-mir-515-2;hsa-mir-516a-1;hsa-mir-516a-2;hsa-mir-516b-1;hsa-mir-516b-2;hsa-mir-519a-2;hsa-mir-519c;hsa-mir-519d;hsa-mir-519e;hsa-mir-520a;hsa-mir-520b;hsa-mir-520c;hsa-mir-520d;hsa-mir-520h;hsa-mir-526a-1;hsa-mir-526a-2;hsa-mir-584;hsa-mir-601;hsa-mir-608;hsa-mir-615;hsa-mir-625;hsa-mir-629;hsa-mir-632;hsa-mir-638;hsa-mir-646;hsa-mir-658;hsa-mir-661;hsa-mir-663a;hsa-mir-708;hsa-mir-718;hsa-mir-874;hsa-mir-922;

module 59

module 60

module 61 hsa-mir-151b;hsa-mir-409;hsa-mir-520c;

module 62

module 63

module 64 hsa-let-7a-1;hsa-let-7a-2;hsa-let-7a-3;hsa-let-7b;hsa-let-7c;hsa-let-7d;hsa-let-7e;hsa-let-7f-1;hsa-let-7f-2;hsa-let-7g;hsa-let-7i;hsa-mir-125a;hsa-mir-1293;hsa-mir-132;hsa-mir-142;hsa-mir-143;hsa-mir-145;hsa-mir-150;hsa-mir-16-2;hsa-mir-210;hsa-mir-221;hsa-mir-222;hsa-mir-23b;hsa-mir-26a-1;hsa-mir-26a-2;hsa-mir-498;hsa-mir-98;

module 65 hsa-mir-10a;hsa-mir-135a-1;hsa-mir-135a-2;hsa-mir-153-2;hsa-mir-182;hsa-mir-20b;hsa-mir-215;hsa-mir-28;hsa-mir-302a;hsa-mir-302b;hsa-mir-302c;hsa-mir-302d;hsa-mir-338;hsa-mir-339;hsa-mir-345;hsa-mir-367;hsa-mir-371a;hsa-mir-372;hsa-mir-373;hsa-mir-376c;hsa-mir-383;hsa-mir-452;hsa-mir-483;hsa-mir-494;hsa-mir-512-1;hsa-mir-512-2;hsa-mir-518a-1;hsa-mir-518a-2;hsa-mir-539;hsa-mir-92b;hsa-mir-935;

module 66

module 67 hsa-mir-134;hsa-mir-326;hsa-mir-361;hsa-mir-376a-1;hsa-mir-376a-2;hsa-mir-381;hsa-mir-491;hsa-mir-539;

module 68

module 69 hsa-mir-103a-1;hsa-mir-103a-2;hsa-mir-103b-1;hsa-mir-103b-2;hsa-mir-107;hsa-mir-10b;hsa-mir-1179;hsa-mir-1180;hsa-mir-1181;hsa-mir-1183;hsa-mir-1184-1;hsa-mir-1207;hsa-mir-1236;hsa-mir-1246;hsa-mir-1247;hsa-mir-1258;hsa-mir-1266;hsa-mir-127;hsa-mir-1273a;hsa-mir-1273c;hsa-mir-1275;hsa-mir-1280;hsa-mir-129-1;hsa-mir-129-2;hsa-mir-1293;hsa-mir-1297;hsa-mir-1299;hsa-mir-1303;hsa-mir-130a;hsa-mir-135a-1;hsa-mir-135a-2;hsa-mir-135b;hsa-mir-136;hsa-mir-137;hsa-mir-139;hsa-mir-140;hsa-mir-144;hsa-mir-146b;hsa-mir-1471;hsa-mir-147b;hsa-mir-148a;hsa-mir-148b;hsa-mir-149;hsa-mir-151;hsa-mir-152;hsa-mir-1827;hsa-mir-185;hsa-mir-186;hsa-mir-189;hsa-mir-18b;hsa-mir-1909;hsa-mir-190a;hsa-mir-190b;hsa-mir-191;hsa-mir-1915;hsa-mir-193a;hsa-mir-194-1;hsa-mir-194-2;hsa-mir-197;hsa-mir-1972-1;hsa-mir-199b;hsa-mir-200;hsa-mir-211;hsa-mir-2110;hsa-mir-215;hsa-mir-216b;hsa-mir-218-1;hsa-mir-218-2;hsa-mir-219-1;hsa-mir-22;hsa-mir-224;hsa-mir-26b;hsa-mir-27b;hsa-mir-28;hsa-mir-2861;hsa-mir-297;hsa-mir-298;hsa-mir-29b;hsa-mir-301a;hsa-mir-3148;hsa-mir-3179-1;hsa-mir-3179-2;hsa-mir-3179-3;hsa-mir-3196;hsa-mir-320a;hsa-mir-324;hsa-mir-326;hsa-mir-328;hsa-mir-330;hsa-mir-335;hsa-mir-337;hsa-mir-338;hsa-mir-339;hsa-mir-33a;hsa-mir-33b;hsa-mir-340;hsa-mir-342;hsa-mir-345;hsa-mir-362;hsa-mir-365a;hsa-mir-365b;hsa-mir-367;hsa-mir-369;hsa-mir-372;hsa-mir-373;hsa-mir-375;hsa-mir-377;hsa-mir-411;hsa-mir-422a;hsa-mir-423;hsa-mir-451a;hsa-mir-454;hsa-mir-4792;hsa-mir-483;hsa-mir-491;hsa-mir-492;hsa-mir-505;hsa-mir-506;hsa-mir-509;hsa-mir-510;hsa-mir-511-1;hsa-mir-511-2;hsa-mir-520a;hsa-mir-523;hsa-mir-525;hsa-mir-542;hsa-mir-545;hsa-mir-548a-1;hsa-mir-548a-2;hsa-mir-548a-3;hsa-mir-548c;hsa-mir-550b-1;hsa-mir-550b-2;hsa-mir-552;hsa-mir-564;hsa-mir-566;hsa-mir-567;hsa-mir-569;hsa-mir-572;hsa-mir-574;hsa-mir-582;hsa-mir-584;hsa-mir-589;hsa-mir-599;hsa-mir-600;hsa-mir-603;hsa-mir-608;hsa-mir-611;hsa-mir-614;hsa-mir-615;hsa-mir-619;hsa-mir-624;hsa-mir-625;hsa-mir-627;hsa-mir-628;hsa-mir-629;hsa-mir-630;hsa-mir-632;hsa-mir-633;hsa-mir-635;hsa-mir-638;hsa-mir-646;hsa-mir-648;hsa-mir-652;hsa-mir-654;hsa-mir-655;hsa-mir-659;hsa-mir-662;hsa-mir-668;hsa-mir-708;hsa-mir-711;hsa-mir-760;hsa-mir-765;hsa-mir-769;hsa-mir-922;hsa-mir-92b;hsa-mir-93;hsa-mir-95;hsa-mir-96;hsa-mir-99a;

module 70 hsa-let-7a-1;hsa-let-7a-2;hsa-let-7a-3;hsa-let-7b;hsa-let-7c;hsa-let-7d;hsa-let-7e;hsa-let-7f-1;hsa-let-7f-2;hsa-let-7g;hsa-let-7i;hsa-mir-125a;hsa-mir-98;

module 71 hsa-mir-133b;hsa-mir-147a;hsa-mir-193a;hsa-mir-193b;hsa-mir-198;hsa-mir-199b;hsa-mir-25;hsa-mir-27a;hsa-mir-301a;hsa-mir-30a;hsa-mir-30b;hsa-mir-30c-1;hsa-mir-30c-2;hsa-mir-30d;hsa-mir-30e;hsa-mir-32;hsa-mir-34b;hsa-mir-34c;hsa-mir-374a;hsa-mir-433;hsa-mir-519b;

module 72

module 73

module 74 hsa-mir-106a;hsa-mir-106b;hsa-mir-125a;hsa-mir-127;hsa-mir-135b;hsa-mir-138-2;hsa-mir-140;hsa-mir-181b-2;hsa-mir-181c;hsa-mir-181d;hsa-mir-182;hsa-mir-183;hsa-mir-185;hsa-mir-18b;hsa-mir-224;hsa-mir-2861;hsa-mir-3196;hsa-mir-378a;hsa-mir-411;hsa-mir-431;hsa-mir-432;hsa-mir-455;hsa-mir-542;hsa-mir-572;hsa-mir-638;hsa-mir-652;hsa-mir-708;hsa-mir-92b;hsa-mir-93;hsa-mir-96;

module 75 hsa-mir-1246;hsa-mir-1286;hsa-mir-1322;hsa-mir-192-2;hsa-mir-193a;hsa-mir-196a-1;hsa-mir-196a-2;hsa-mir-28;hsa-mir-382;hsa-mir-454;hsa-mir-499a;hsa-mir-548d-1;hsa-mir-548d-2;hsa-mir-593;hsa-mir-617;hsa-mir-720;hsa-mir-758;hsa-mir-766;

module 76 hsa-mir-100;hsa-mir-127;hsa-mir-1299;hsa-mir-130a;hsa-mir-130b;hsa-mir-148a;hsa-mir-148b;hsa-mir-151a;hsa-mir-152;hsa-mir-153-2;hsa-mir-187;hsa-mir-191;hsa-mir-192;hsa-mir-194-1;hsa-mir-194-2;hsa-mir-203;hsa-mir-215;hsa-mir-22;hsa-mir-300;hsa-mir-320a;hsa-mir-335;hsa-mir-339;hsa-mir-367;hsa-mir-376c;hsa-mir-383;hsa-mir-449a;hsa-mir-449b;hsa-mir-488;hsa-mir-502;hsa-mir-561;hsa-mir-625;hsa-mir-629;hsa-mir-636;hsa-mir-642a;hsa-mir-99a;hsa-mir-99b;

module 77 hsa-mir-30e;hsa-mir-488;

module 78 hsa-mir-106a;hsa-mir-10a;hsa-mir-10b;hsa-mir-1260a;hsa-mir-127;hsa-mir-1305;hsa-mir-135a-1;hsa-mir-135b;hsa-mir-144;hsa-mir-153-1;hsa-mir-153-2;hsa-mir-181b-1;hsa-mir-181b-2;hsa-mir-186;hsa-mir-193a;hsa-mir-193b;hsa-mir-199b;hsa-mir-219-1;hsa-mir-219-2;hsa-mir-224;hsa-mir-3163;hsa-mir-323a;hsa-mir-326;hsa-mir-338;hsa-mir-33a;hsa-mir-33b;hsa-mir-361;hsa-mir-376a-1;hsa-mir-376a-2;hsa-mir-376c;hsa-mir-379;hsa-mir-383;hsa-mir-409;hsa-mir-452;hsa-mir-494;hsa-mir-495;hsa-mir-504;hsa-mir-512-2;hsa-mir-539;hsa-mir-548d-1;hsa-mir-548d-2;hsa-mir-873;hsa-mir-92b;hsa-mir-935;

module 79 hsa-mir-101-1;hsa-mir-101-2;hsa-mir-106a;hsa-mir-106b;hsa-mir-107;hsa-mir-124-1;hsa-mir-124-2;hsa-mir-124-3;hsa-mir-1256;hsa-mir-127;hsa-mir-1296;hsa-mir-130a;hsa-mir-130b;hsa-mir-132;hsa-mir-135b;hsa-mir-148b;hsa-mir-151a;hsa-mir-151b;hsa-mir-152;hsa-mir-153-1;hsa-mir-153-2;hsa-mir-154;hsa-mir-185;hsa-mir-191;hsa-mir-193a;hsa-mir-193b;hsa-mir-194-1;hsa-mir-194-2;hsa-mir-196b;hsa-mir-198;hsa-mir-202;hsa-mir-204;hsa-mir-218-1;hsa-mir-22;hsa-mir-224;hsa-mir-23b;hsa-mir-25;hsa-mir-27a;hsa-mir-27b;hsa-mir-296;hsa-mir-299;hsa-mir-301a;hsa-mir-301b;hsa-mir-30c-1;hsa-mir-30c-2;hsa-mir-32;hsa-mir-320a;hsa-mir-320e;hsa-mir-330;hsa-mir-331;hsa-mir-335;hsa-mir-34b;hsa-mir-373;hsa-mir-374a;hsa-mir-376c;hsa-mir-378a;hsa-mir-383;hsa-mir-409;hsa-mir-449a;hsa-mir-450a-1;hsa-mir-450a-2;hsa-mir-450b;hsa-mir-452;hsa-mir-486;hsa-mir-488;hsa-mir-495;hsa-mir-515-1;hsa-mir-515-2;hsa-mir-519c;hsa-mir-519d;hsa-mir-519e;hsa-mir-520c;hsa-mir-521-1;hsa-mir-521-2;hsa-mir-526a-1;hsa-mir-526a-2;hsa-mir-574;hsa-mir-616;hsa-mir-629;hsa-mir-642a;hsa-mir-642b;hsa-mir-647;hsa-mir-661;hsa-mir-708;hsa-mir-93;hsa-mir-98;

module 80 hsa-mir-103a-1;hsa-mir-103a-2;hsa-mir-103b-1;hsa-mir-103b-2;hsa-mir-106b;hsa-mir-107;hsa-mir-132;hsa-mir-134;hsa-mir-137;hsa-mir-15b;hsa-mir-181b-1;hsa-mir-195;hsa-mir-198;hsa-mir-206;hsa-mir-20b;hsa-mir-212;hsa-mir-219-1;hsa-mir-219-2;hsa-mir-24-2;hsa-mir-26b;hsa-mir-29a;hsa-mir-29b-1;hsa-mir-29b-2;hsa-mir-29c;hsa-mir-30a;hsa-mir-30b;hsa-mir-30c-1;hsa-mir-30c-2;hsa-mir-30d;hsa-mir-30e;hsa-mir-33b;hsa-mir-346;hsa-mir-34b;hsa-mir-34c;hsa-mir-7-1;hsa-mir-7-2;hsa-mir-7-3;hsa-mir-9-1;hsa-mir-9-2;hsa-mir-9-3;hsa-mir-92b;

module 81 hsa-mir-101-1;hsa-mir-101-2;hsa-mir-106a;hsa-mir-10a;hsa-mir-10b;hsa-mir-1271;hsa-mir-129-1;hsa-mir-129-2;hsa-mir-130a;hsa-mir-130b;hsa-mir-137;hsa-mir-138-1;hsa-mir-138-2;hsa-mir-139;hsa-mir-142;hsa-mir-146b;hsa-mir-149;hsa-mir-181d;hsa-mir-184;hsa-mir-18b;hsa-mir-197;hsa-mir-204;hsa-mir-205;hsa-mir-211;hsa-mir-218-1;hsa-mir-218-2;hsa-mir-26b;hsa-mir-27b;hsa-mir-302a;hsa-mir-302b;hsa-mir-302c;hsa-mir-302d;hsa-mir-302e;hsa-mir-302f;hsa-mir-31;hsa-mir-32;hsa-mir-323b;hsa-mir-328;hsa-mir-363;hsa-mir-370;hsa-mir-371a;hsa-mir-375;hsa-mir-421;hsa-mir-423;hsa-mir-455;hsa-mir-489;hsa-mir-491;hsa-mir-499b;hsa-mir-504;hsa-mir-519a-1;hsa-mir-519a-2;hsa-mir-542;hsa-mir-7-1;hsa-mir-744;hsa-mir-874;hsa-mir-885;hsa-mir-9-1;hsa-mir-9-2;hsa-mir-9-3;hsa-mir-95;

module 82

module 83 hsa-mir-504;

module 84 hsa-let-7a-1;hsa-let-7a-2;hsa-let-7a-3;hsa-let-7b;hsa-let-7c;hsa-let-7d;hsa-let-7e;hsa-let-7f-1;hsa-let-7f-2;hsa-let-7g;hsa-let-7i;hsa-mir-98;

module 85 hsa-mir-193a;hsa-mir-193b;

module 86 hsa-mir-1-1;hsa-mir-1-2;hsa-mir-103a-1;hsa-mir-103a-2;hsa-mir-133a-1;hsa-mir-133a-2;hsa-mir-133b;hsa-mir-195;hsa-mir-199a-1;hsa-mir-199a-2;hsa-mir-206;hsa-mir-208a;hsa-mir-208b;hsa-mir-210;hsa-mir-296;hsa-mir-328;hsa-mir-335;hsa-mir-34c;hsa-mir-378a;hsa-mir-422a;hsa-mir-431;hsa-mir-499a;hsa-mir-637;hsa-mir-652;

module 87 hsa-mir-103a-1;hsa-mir-103a-2;hsa-mir-103b-1;hsa-mir-103b-2;hsa-mir-152;hsa-mir-153-1;hsa-mir-182;hsa-mir-183;hsa-mir-186;hsa-mir-193a;hsa-mir-193b;hsa-mir-194-1;hsa-mir-194-2;hsa-mir-204;hsa-mir-208a;hsa-mir-216a;hsa-mir-216b;hsa-mir-25;hsa-mir-30c-1;hsa-mir-32;hsa-mir-34b;hsa-mir-378a;hsa-mir-625;hsa-mir-633;hsa-mir-96;hsa-mir-99a;

module 88

module 89 hsa-mir-203;hsa-mir-99a;

module 90

module 91

module 92 hsa-mir-107;hsa-mir-10b;hsa-mir-125a;hsa-mir-129-2;hsa-mir-130a;hsa-mir-130b;hsa-mir-132;hsa-mir-134;hsa-mir-135a-1;hsa-mir-135a-2;hsa-mir-136;hsa-mir-139;hsa-mir-150;hsa-mir-15b;hsa-mir-186;hsa-mir-187;hsa-mir-18b;hsa-mir-192;hsa-mir-195;hsa-mir-196a-1;hsa-mir-196a-2;hsa-mir-197;hsa-mir-199b;hsa-mir-204;hsa-mir-208a;hsa-mir-208b;hsa-mir-210;hsa-mir-211;hsa-mir-212;hsa-mir-215;hsa-mir-216a;hsa-mir-218-1;hsa-mir-218-2;hsa-mir-22;hsa-mir-23a;hsa-mir-23b;hsa-mir-24-2;hsa-mir-26b;hsa-mir-27a;hsa-mir-28;hsa-mir-296;hsa-mir-297;hsa-mir-299;hsa-mir-300;hsa-mir-302a;hsa-mir-302b;hsa-mir-302c;hsa-mir-30a;hsa-mir-30b;hsa-mir-30c-1;hsa-mir-30c-2;hsa-mir-30e;hsa-mir-32;hsa-mir-320a;hsa-mir-325;hsa-mir-328;hsa-mir-330;hsa-mir-339;hsa-mir-340;hsa-mir-34b;hsa-mir-365a;hsa-mir-365b;hsa-mir-367;hsa-mir-372;hsa-mir-373;hsa-mir-377;hsa-mir-381;hsa-mir-382;hsa-mir-423;hsa-mir-424;hsa-mir-432;hsa-mir-452;hsa-mir-494;hsa-mir-497;hsa-mir-499a;hsa-mir-500a;hsa-mir-503;hsa-mir-507;hsa-mir-508;hsa-mir-512-1;hsa-mir-512-2;hsa-mir-515-1;hsa-mir-515-2;hsa-mir-520a;hsa-mir-520b;hsa-mir-520c;hsa-mir-520d;hsa-mir-520e;hsa-mir-520f;hsa-mir-520g;hsa-mir-520h;hsa-mir-523;hsa-mir-525;hsa-mir-526a-1;hsa-mir-526a-2;hsa-mir-526b;hsa-mir-637;hsa-mir-650;hsa-mir-661;hsa-mir-92a-2;hsa-mir-92b;hsa-mir-98;

module 93 hsa-mir-103a-1;hsa-mir-103a-2;hsa-mir-103b-1;hsa-mir-103b-2;hsa-mir-106a;hsa-mir-106b;hsa-mir-107;hsa-mir-128-1;hsa-mir-128-2;hsa-mir-132;hsa-mir-137;hsa-mir-144;hsa-mir-181b-1;hsa-mir-181b-2;hsa-mir-181c;hsa-mir-196b;hsa-mir-206;hsa-mir-20b;hsa-mir-24-1;hsa-mir-24-2;hsa-mir-30e;hsa-mir-325;hsa-mir-34c;hsa-mir-411;hsa-mir-518c;hsa-mir-590;

module 94 hsa-mir-103b-1;hsa-mir-103b-2;hsa-mir-1273c;hsa-mir-1275;hsa-mir-1915;hsa-mir-216b;hsa-mir-330;hsa-mir-567;hsa-mir-584;hsa-mir-646;hsa-mir-95;

module 95 hsa-mir-126;hsa-mir-143;hsa-mir-145;hsa-mir-192;hsa-mir-21;hsa-mir-210;hsa-mir-221;hsa-mir-29a;hsa-mir-29b-1;hsa-mir-29b-2;hsa-mir-29c;hsa-mir-34a;hsa-mir-34c;hsa-mir-377;

module 96 hsa-mir-103a-1;hsa-mir-103a-2;hsa-mir-103b-1;hsa-mir-103b-2;hsa-mir-107;hsa-mir-125b-1;hsa-mir-125b-2;hsa-mir-130a;hsa-mir-146b;hsa-mir-148a;hsa-mir-151a;hsa-mir-193a;hsa-mir-193b;hsa-mir-206;hsa-mir-208a;hsa-mir-22;hsa-mir-27b;hsa-mir-32;hsa-mir-335;hsa-mir-33a;hsa-mir-33b;hsa-mir-34b;hsa-mir-34c;hsa-mir-378a;hsa-mir-381;hsa-mir-633;

module 97 hsa-mir-216b;hsa-mir-32;

module 98 hsa-mir-1260a;hsa-mir-1305;hsa-mir-134;hsa-mir-184;hsa-mir-200;hsa-mir-208a;hsa-mir-208b;hsa-mir-3163;hsa-mir-32;hsa-mir-323a;hsa-mir-323b;hsa-mir-328;hsa-mir-329-1;hsa-mir-329-2;hsa-mir-381;hsa-mir-425;hsa-mir-452;hsa-mir-455;hsa-mir-504;hsa-mir-539;hsa-mir-548b;hsa-mir-873;hsa-mir-885;hsa-mir-95;

module 99 hsa-mir-1179;hsa-mir-1224;hsa-mir-130b;hsa-mir-134;hsa-mir-142;hsa-mir-146a;hsa-mir-150;hsa-mir-184;hsa-mir-185;hsa-mir-195;hsa-mir-197;hsa-mir-198;hsa-mir-208b;hsa-mir-210;hsa-mir-223;hsa-mir-23a;hsa-mir-296;hsa-mir-30a;hsa-mir-30d;hsa-mir-324;hsa-mir-330;hsa-mir-346;hsa-mir-365a;hsa-mir-365b;hsa-mir-423;hsa-mir-433;hsa-mir-484;hsa-mir-486;hsa-mir-494;hsa-mir-498;hsa-mir-500a;hsa-mir-516a-1;hsa-mir-516a-2;hsa-mir-516b-1;hsa-mir-516b-2;hsa-mir-518b;hsa-mir-518c;hsa-mir-557;hsa-mir-575;hsa-mir-583;hsa-mir-596;hsa-mir-600;hsa-mir-601;hsa-mir-602;hsa-mir-608;hsa-mir-611;hsa-mir-612;hsa-mir-615;hsa-mir-622;hsa-mir-629;hsa-mir-630;hsa-mir-637;hsa-mir-638;hsa-mir-642a;hsa-mir-654;hsa-mir-657;hsa-mir-658;hsa-mir-662;hsa-mir-663a;hsa-mir-769;hsa-mir-922;hsa-mir-99a;

module 100 hsa-mir-101-1;hsa-mir-127;hsa-mir-128-1;hsa-mir-128-2;hsa-mir-1299;hsa-mir-130b;hsa-mir-140;hsa-mir-148b;hsa-mir-151a;hsa-mir-152;hsa-mir-153-1;hsa-mir-153-2;hsa-mir-181d;hsa-mir-185;hsa-mir-187;hsa-mir-191;hsa-mir-194-1;hsa-mir-194-2;hsa-mir-20b;hsa-mir-215;hsa-mir-218-1;hsa-mir-219-1;hsa-mir-22;hsa-mir-224;hsa-mir-25;hsa-mir-296;hsa-mir-299;hsa-mir-300;hsa-mir-301a;hsa-mir-301b;hsa-mir-302a;hsa-mir-302b;hsa-mir-302c;hsa-mir-302d;hsa-mir-30e;hsa-mir-320a;hsa-mir-335;hsa-mir-338;hsa-mir-339;hsa-mir-340;hsa-mir-367;hsa-mir-370;hsa-mir-374a;hsa-mir-376c;hsa-mir-383;hsa-mir-409;hsa-mir-433;hsa-mir-449a;hsa-mir-449b;hsa-mir-486;hsa-mir-488;hsa-mir-495;hsa-mir-499a;hsa-mir-502;hsa-mir-506;hsa-mir-515-1;hsa-mir-515-2;hsa-mir-516a-1;hsa-mir-516a-2;hsa-mir-516b-1;hsa-mir-516b-2;hsa-mir-518c;hsa-mir-519a-2;hsa-mir-519e;hsa-mir-526a-1;hsa-mir-526a-2;hsa-mir-532;hsa-mir-542;hsa-mir-601;hsa-mir-622;hsa-mir-625;hsa-mir-629;hsa-mir-642a;hsa-mir-658;hsa-mir-661;hsa-mir-922;hsa-mir-92b;hsa-mir-93;hsa-mir-99b;

module 101 hsa-mir-122;hsa-mir-146a;hsa-mir-146b;hsa-mir-148a;hsa-mir-150;hsa-mir-155;hsa-mir-15a;hsa-mir-16-1;hsa-mir-16-2;hsa-mir-181a-1;hsa-mir-181a-2;hsa-mir-22;hsa-mir-221;hsa-mir-222;hsa-mir-27b;hsa-mir-326;hsa-mir-34a;hsa-mir-422a;hsa-mir-572;hsa-mir-599;hsa-mir-614;hsa-mir-648;

module 102 hsa-mir-885;

module 103 hsa-mir-106a;hsa-mir-106b;hsa-mir-129-1;hsa-mir-129-2;hsa-mir-132;hsa-mir-134;hsa-mir-139;hsa-mir-140;hsa-mir-148b;hsa-mir-15b;hsa-mir-181b-1;hsa-mir-181d;hsa-mir-184;hsa-mir-193b;hsa-mir-198;hsa-mir-212;hsa-mir-23a;hsa-mir-23b;hsa-mir-345;hsa-mir-346;hsa-mir-363;hsa-mir-381;hsa-mir-411;hsa-mir-431;hsa-mir-432;hsa-mir-484;hsa-mir-486;hsa-mir-490;hsa-mir-539;hsa-mir-542;hsa-mir-550a-1;hsa-mir-550a-2;hsa-mir-598;hsa-mir-652;hsa-mir-663a;hsa-mir-664;hsa-mir-7-1;hsa-mir-7-2;hsa-mir-7-3;hsa-mir-92a-2;hsa-mir-92b;hsa-mir-93;hsa-mir-95;

module 104 hsa-mir-9-1;hsa-mir-9-2;

module 105

module 106 hsa-mir-124-1;hsa-mir-124-2;hsa-mir-124-3;hsa-mir-152;hsa-mir-153-1;hsa-mir-153-2;hsa-mir-494;hsa-mir-497;

module 107 hsa-mir-103a-1;hsa-mir-103a-2;hsa-mir-103b-1;hsa-mir-103b-2;hsa-mir-128-1;hsa-mir-128-2;hsa-mir-130b;hsa-mir-152;hsa-mir-153-1;hsa-mir-153-2;hsa-mir-182;hsa-mir-183;hsa-mir-204;hsa-mir-20b;hsa-mir-302a;hsa-mir-302c;hsa-mir-302d;hsa-mir-302e;hsa-mir-302f;hsa-mir-325;hsa-mir-383;hsa-mir-411;hsa-mir-518c;hsa-mir-625;hsa-mir-96;

module 108

module 109 hsa-mir-103b-1;hsa-mir-103b-2;hsa-mir-190a;hsa-mir-216b;hsa-mir-584;hsa-mir-630;hsa-mir-95;

module 110

module 111 hsa-let-7a;hsa-mir-106a;hsa-mir-107;hsa-mir-10b;hsa-mir-1228;hsa-mir-127;hsa-mir-128-1;hsa-mir-128-2;hsa-mir-129-1;hsa-mir-129-2;hsa-mir-130b;hsa-mir-135a-1;hsa-mir-135a-2;hsa-mir-135b;hsa-mir-137;hsa-mir-139;hsa-mir-140;hsa-mir-1471;hsa-mir-148b;hsa-mir-149;hsa-mir-151a;hsa-mir-151b;hsa-mir-181c;hsa-mir-184;hsa-mir-185;hsa-mir-186;hsa-mir-187;hsa-mir-18b;hsa-mir-191;hsa-mir-193b;hsa-mir-194-1;hsa-mir-194-2;hsa-mir-196b;hsa-mir-204;hsa-mir-20b;hsa-mir-218-1;hsa-mir-218-2;hsa-mir-224;hsa-mir-26b;hsa-mir-27a;hsa-mir-296;hsa-mir-301a;hsa-mir-302a;hsa-mir-302b;hsa-mir-302c;hsa-mir-302d;hsa-mir-302e;hsa-mir-302f;hsa-mir-32;hsa-mir-320a;hsa-mir-328;hsa-mir-331;hsa-mir-335;hsa-mir-340;hsa-mir-363;hsa-mir-370;hsa-mir-371a;hsa-mir-372;hsa-mir-373;hsa-mir-374a;hsa-mir-374b;hsa-mir-376c;hsa-mir-409;hsa-mir-421;hsa-mir-433;hsa-mir-449a;hsa-mir-449b;hsa-mir-449c;hsa-mir-451a;hsa-mir-487b;hsa-mir-494;hsa-mir-495;hsa-mir-503;hsa-mir-513a-1;hsa-mir-513a-2;hsa-mir-513b;hsa-mir-513c;hsa-mir-515-1;hsa-mir-515-2;hsa-mir-516a-1;hsa-mir-516a-2;hsa-mir-516b-1;hsa-mir-516b-2;hsa-mir-517a;hsa-mir-518c;hsa-mir-518f;hsa-mir-519a-1;hsa-mir-519a-2;hsa-mir-519c;hsa-mir-519e;hsa-mir-520a;hsa-mir-520c;hsa-mir-520d;hsa-mir-524;hsa-mir-526a-1;hsa-mir-526a-2;hsa-mir-544a;hsa-mir-544b;hsa-mir-545;hsa-mir-551a;hsa-mir-570;hsa-mir-574;hsa-mir-577;hsa-mir-591;hsa-mir-595;hsa-mir-601;hsa-mir-605;hsa-mir-610;hsa-mir-622;hsa-mir-625;hsa-mir-629;hsa-mir-640;hsa-mir-650;hsa-mir-658;hsa-mir-661;hsa-mir-744;hsa-mir-874;hsa-mir-922;hsa-mir-93;hsa-mir-938;hsa-mir-95;

module 112 hsa-mir-100;hsa-mir-101-1;hsa-mir-101-2;hsa-mir-135b;hsa-mir-141;hsa-mir-142;hsa-mir-182;hsa-mir-183;hsa-mir-192;hsa-mir-196a-1;hsa-mir-196a-2;hsa-mir-200a;hsa-mir-200b;hsa-mir-200c;hsa-mir-203;hsa-mir-205;hsa-mir-23b;hsa-mir-31;hsa-mir-375;hsa-mir-429;hsa-mir-569;hsa-mir-96;hsa-mir-99a;

module 113 hsa-let-7a;hsa-let-7a-1;hsa-let-7a-2;hsa-let-7a-3;hsa-let-7b;hsa-let-7c;hsa-let-7d;hsa-let-7e;hsa-let-7f-1;hsa-let-7f-2;hsa-let-7g;hsa-let-7i;hsa-mir-100;hsa-mir-101-1;hsa-mir-101-2;hsa-mir-103a-1;hsa-mir-103a-2;hsa-mir-103b-1;hsa-mir-103b-2;hsa-mir-105-1;hsa-mir-105-2;hsa-mir-106a;hsa-mir-106b;hsa-mir-107;hsa-mir-10a;hsa-mir-10b;hsa-mir-1179;hsa-mir-1180;hsa-mir-1181;hsa-mir-1183;hsa-mir-1184-1;hsa-mir-1202;hsa-mir-1207;hsa-mir-122;hsa-mir-1224;hsa-mir-1227;hsa-mir-1228;hsa-mir-1229;hsa-mir-1231;hsa-mir-1233-1;hsa-mir-1233-2;hsa-mir-1234;hsa-mir-1236;hsa-mir-124-1;hsa-mir-124-2;hsa-mir-124-3;hsa-mir-1247;hsa-mir-1249;hsa-mir-124a-2;hsa-mir-124a-3;hsa-mir-1254-1;hsa-mir-1258;hsa-mir-125a;hsa-mir-126;hsa-mir-1260a;hsa-mir-1266;hsa-mir-127;hsa-mir-1271;hsa-mir-1273a;hsa-mir-1273c;hsa-mir-1275;hsa-mir-128;hsa-mir-128-1;hsa-mir-128-2;hsa-mir-1280;hsa-mir-1285-1;hsa-mir-1285-2;hsa-mir-1286;hsa-mir-129-1;hsa-mir-129-2;hsa-mir-1290;hsa-mir-1293;hsa-mir-1297;hsa-mir-1299;hsa-mir-1301;hsa-mir-1303;hsa-mir-1305;hsa-mir-130a;hsa-mir-130b;hsa-mir-132;hsa-mir-1322;hsa-mir-134;hsa-mir-135a-1;hsa-mir-135a-2;hsa-mir-135b;hsa-mir-136;hsa-mir-137;hsa-mir-138-1;hsa-mir-138-2;hsa-mir-139;hsa-mir-140;hsa-mir-141;hsa-mir-142;hsa-mir-143;hsa-mir-144;hsa-mir-145;hsa-mir-146b;hsa-mir-1471;hsa-mir-147a;hsa-mir-147b;hsa-mir-148a;hsa-mir-148b;hsa-mir-149;hsa-mir-150;hsa-mir-151;hsa-mir-151a;hsa-mir-151b;hsa-mir-152;hsa-mir-153-1;hsa-mir-153-2;hsa-mir-154;hsa-mir-15b;hsa-mir-181a-1;hsa-mir-181a-2;hsa-mir-181b-1;hsa-mir-181b-2;hsa-mir-181c;hsa-mir-181d;hsa-mir-182;hsa-mir-1827;hsa-mir-183;hsa-mir-184;hsa-mir-185;hsa-mir-186;hsa-mir-187;hsa-mir-188;hsa-mir-189;hsa-mir-18a;hsa-mir-18b;hsa-mir-1909;hsa-mir-190a;hsa-mir-190b;hsa-mir-191;hsa-mir-1915;hsa-mir-192;hsa-mir-192-2;hsa-mir-193a;hsa-mir-193b;hsa-mir-194-1;hsa-mir-194-2;hsa-mir-195;hsa-mir-196a-1;hsa-mir-196a-2;hsa-mir-196b;hsa-mir-197;hsa-mir-1972-1;hsa-mir-198;hsa-mir-199a-1;hsa-mir-199b;hsa-mir-200;hsa-mir-200a;hsa-mir-200b;hsa-mir-200c;hsa-mir-202;hsa-mir-203;hsa-mir-204;hsa-mir-205;hsa-mir-206;hsa-mir-208a;hsa-mir-208b;hsa-mir-20b;hsa-mir-210;hsa-mir-211;hsa-mir-2110;hsa-mir-212;hsa-mir-214;hsa-mir-215;hsa-mir-216a;hsa-mir-216b;hsa-mir-217;hsa-mir-218-1;hsa-mir-218-2;hsa-mir-219-1;hsa-mir-219-2;hsa-mir-22;hsa-mir-221;hsa-mir-222;hsa-mir-223;hsa-mir-224;hsa-mir-23a;hsa-mir-23b;hsa-mir-24-1;hsa-mir-24-2;hsa-mir-25;hsa-mir-26;hsa-mir-26a-1;hsa-mir-26a-2;hsa-mir-26b;hsa-mir-27a;hsa-mir-27b;hsa-mir-28;hsa-mir-2861;hsa-mir-296;hsa-mir-297;hsa-mir-298;hsa-mir-299;hsa-mir-29b;hsa-mir-300;hsa-mir-301a;hsa-mir-301b;hsa-mir-302a;hsa-mir-302b;hsa-mir-302c;hsa-mir-302d;hsa-mir-302e;hsa-mir-302f;hsa-mir-30a;hsa-mir-30b;hsa-mir-30c-1;hsa-mir-30c-2;hsa-mir-30d;hsa-mir-30e;hsa-mir-31;hsa-mir-3148;hsa-mir-3151;hsa-mir-3163;hsa-mir-3179-1;hsa-mir-3179-2;hsa-mir-3179-3;hsa-mir-3196;hsa-mir-32;hsa-mir-320a;hsa-mir-320b-1;hsa-mir-320b-2;hsa-mir-320c-1;hsa-mir-320c-2;hsa-mir-320d-1;hsa-mir-320d-2;hsa-mir-320e;hsa-mir-323a;hsa-mir-323b;hsa-mir-324;hsa-mir-325;hsa-mir-326;hsa-mir-328;hsa-mir-329-1;hsa-mir-329-2;hsa-mir-330;hsa-mir-331;hsa-mir-335;hsa-mir-337;hsa-mir-338;hsa-mir-339;hsa-mir-33a;hsa-mir-33b;hsa-mir-340;hsa-mir-342;hsa-mir-345;hsa-mir-346;hsa-mir-34a;hsa-mir-34b;hsa-mir-34c;hsa-mir-361;hsa-mir-362;hsa-mir-363;hsa-mir-365a;hsa-mir-365b;hsa-mir-367;hsa-mir-369;hsa-mir-370;hsa-mir-371;hsa-mir-371a;hsa-mir-372;hsa-mir-373;hsa-mir-374a;hsa-mir-375;hsa-mir-376a-1;hsa-mir-376a-2;hsa-mir-376b;hsa-mir-376c;hsa-mir-377;hsa-mir-378a;hsa-mir-379;hsa-mir-381;hsa-mir-382;hsa-mir-383;hsa-mir-384;hsa-mir-3940;hsa-mir-409;hsa-mir-410;hsa-mir-411;hsa-mir-421;hsa-mir-422a;hsa-mir-423;hsa-mir-424;hsa-mir-425;hsa-mir-429;hsa-mir-431;hsa-mir-432;hsa-mir-433;hsa-mir-448;hsa-mir-449a;hsa-mir-449b;hsa-mir-449c;hsa-mir-450a-1;hsa-mir-450a-2;hsa-mir-450b;hsa-mir-451;hsa-mir-451a;hsa-mir-451b;hsa-mir-452;hsa-mir-455;hsa-mir-4792;hsa-mir-483;hsa-mir-484;hsa-mir-485;hsa-mir-486;hsa-mir-487a;hsa-mir-487b;hsa-mir-488;hsa-mir-489;hsa-mir-490;hsa-mir-491;hsa-mir-492;hsa-mir-493;hsa-mir-494;hsa-mir-495;hsa-mir-497;hsa-mir-498;hsa-mir-499a;hsa-mir-499b;hsa-mir-500a;hsa-mir-500b;hsa-mir-501;hsa-mir-502;hsa-mir-503;hsa-mir-504;hsa-mir-505;hsa-mir-506;hsa-mir-507;hsa-mir-508;hsa-mir-509;hsa-mir-510;hsa-mir-511-1;hsa-mir-511-2;hsa-mir-512-1;hsa-mir-512-2;hsa-mir-513a-1;hsa-mir-513a-2;hsa-mir-513b;hsa-mir-513c;hsa-mir-515-1;hsa-mir-515-2;hsa-mir-516a-1;hsa-mir-516a-2;hsa-mir-516b-1;hsa-mir-516b-2;hsa-mir-517a;hsa-mir-517c;hsa-mir-518a-1;hsa-mir-518a-2;hsa-mir-518b;hsa-mir-518c;hsa-mir-518e;hsa-mir-518f;hsa-mir-519a-1;hsa-mir-519a-2;hsa-mir-519b;hsa-mir-519c;hsa-mir-519d;hsa-mir-519e;hsa-mir-520a;hsa-mir-520b;hsa-mir-520c;hsa-mir-520d;hsa-mir-520e;hsa-mir-520f;hsa-mir-520g;hsa-mir-520h;hsa-mir-522;hsa-mir-523;hsa-mir-524;hsa-mir-525;hsa-mir-526a-1;hsa-mir-526a-2;hsa-mir-526b;hsa-mir-527;hsa-mir-532;hsa-mir-539;hsa-mir-542;hsa-mir-544a;hsa-mir-544b;hsa-mir-545;hsa-mir-548a-1;hsa-mir-548a-2;hsa-mir-548a-3;hsa-mir-548b;hsa-mir-548c;hsa-mir-548d-1;hsa-mir-548d-2;hsa-mir-550a-1;hsa-mir-550a-2;hsa-mir-550a-3;hsa-mir-550b-1;hsa-mir-550b-2;hsa-mir-551a;hsa-mir-552;hsa-mir-557;hsa-mir-561;hsa-mir-564;hsa-mir-566;hsa-mir-567;hsa-mir-569;hsa-mir-570;hsa-mir-571;hsa-mir-572;hsa-mir-574;hsa-mir-575;hsa-mir-577;hsa-mir-582;hsa-mir-583;hsa-mir-584;hsa-mir-589;hsa-mir-590;hsa-mir-591;hsa-mir-592;hsa-mir-593;hsa-mir-595;hsa-mir-596;hsa-mir-598;hsa-mir-599;hsa-mir-600;hsa-mir-601;hsa-mir-602;hsa-mir-603;hsa-mir-605;hsa-mir-608;hsa-mir-610;hsa-mir-611;hsa-mir-612;hsa-mir-614;hsa-mir-615;hsa-mir-617;hsa-mir-619;hsa-mir-621;hsa-mir-622;hsa-mir-624;hsa-mir-625;hsa-mir-627;hsa-mir-628;hsa-mir-629;hsa-mir-630;hsa-mir-632;hsa-mir-633;hsa-mir-635;hsa-mir-636;hsa-mir-637;hsa-mir-638;hsa-mir-640;hsa-mir-642b;hsa-mir-646;hsa-mir-648;hsa-mir-650;hsa-mir-652;hsa-mir-654;hsa-mir-655;hsa-mir-657;hsa-mir-658;hsa-mir-659;hsa-mir-660;hsa-mir-661;hsa-mir-662;hsa-mir-663;hsa-mir-663a;hsa-mir-663b;hsa-mir-664;hsa-mir-668;hsa-mir-671;hsa-mir-675;hsa-mir-7-1;hsa-mir-7-2;hsa-mir-7-3;hsa-mir-708;hsa-mir-711;hsa-mir-720;hsa-mir-744;hsa-mir-758;hsa-mir-760;hsa-mir-765;hsa-mir-766;hsa-mir-767;hsa-mir-769;hsa-mir-873;hsa-mir-874;hsa-mir-885;hsa-mir-9-1;hsa-mir-9-2;hsa-mir-9-3;hsa-mir-92;hsa-mir-922;hsa-mir-92b;hsa-mir-93;hsa-mir-933;hsa-mir-935;hsa-mir-938;hsa-mir-941-1;hsa-mir-941-3;hsa-mir-941-4;hsa-mir-942;hsa-mir-944;hsa-mir-95;hsa-mir-96;hsa-mir-98;hsa-mir-99a;hsa-mir-99b;

module 114 hsa-mir-151a;hsa-mir-199a-1;hsa-mir-199a-2;hsa-mir-199b;hsa-mir-214;hsa-mir-22;hsa-mir-28;hsa-mir-301a;hsa-mir-34b;hsa-mir-34c;hsa-mir-374a;hsa-mir-433;

module 115

module 116 hsa-let-7a-1;hsa-let-7a-2;hsa-let-7a-3;hsa-let-7b;hsa-let-7c;hsa-let-7d;hsa-let-7e;hsa-let-7f-1;hsa-let-7f-2;hsa-let-7g;hsa-let-7i;hsa-mir-125a;hsa-mir-142;hsa-mir-143;hsa-mir-145;hsa-mir-150;hsa-mir-16-2;hsa-mir-210;hsa-mir-221;hsa-mir-222;hsa-mir-26a-1;hsa-mir-26a-2;hsa-mir-98;

module 117 hsa-mir-101-1;hsa-mir-101-2;hsa-mir-1180;hsa-mir-1184-1;hsa-mir-1236;hsa-mir-1246;hsa-mir-1247;hsa-mir-1266;hsa-mir-1271;hsa-mir-1273a;hsa-mir-1293;hsa-mir-1299;hsa-mir-1302-1;hsa-mir-1302-2;hsa-mir-1302-3;hsa-mir-1302-4;hsa-mir-1302-5;hsa-mir-1302-6;hsa-mir-1302-7;hsa-mir-1302-8;hsa-mir-146b;hsa-mir-147b;hsa-mir-148a;hsa-mir-148b;hsa-mir-150;hsa-mir-151;hsa-mir-181b-1;hsa-mir-181b-2;hsa-mir-1827;hsa-mir-189;hsa-mir-18b;hsa-mir-191;hsa-mir-196b;hsa-mir-1972-1;hsa-mir-210;hsa-mir-219-1;hsa-mir-25;hsa-mir-26b;hsa-mir-27a;hsa-mir-27b;hsa-mir-297;hsa-mir-30a;hsa-mir-30b;hsa-mir-30c-1;hsa-mir-30c-2;hsa-mir-3148;hsa-mir-3179-1;hsa-mir-320c-2;hsa-mir-324;hsa-mir-338;hsa-mir-33a;hsa-mir-33b;hsa-mir-342;hsa-mir-345;hsa-mir-362;hsa-mir-374b;hsa-mir-378a;hsa-mir-421;hsa-mir-433;hsa-mir-452;hsa-mir-454;hsa-mir-4792;hsa-mir-487a;hsa-mir-501;hsa-mir-505;hsa-mir-519a-1;hsa-mir-519a-2;hsa-mir-566;hsa-mir-569;hsa-mir-582;hsa-mir-589;hsa-mir-603;hsa-mir-619;hsa-mir-627;hsa-mir-628;hsa-mir-633;hsa-mir-635;hsa-mir-650;hsa-mir-655;hsa-mir-660;hsa-mir-7-1;hsa-mir-7-2;hsa-mir-7-3;hsa-mir-711;hsa-mir-744;hsa-mir-760;hsa-mir-942;

module 118 hsa-mir-135b;hsa-mir-182;hsa-mir-183;hsa-mir-483;hsa-mir-548d-1;hsa-mir-548d-2;hsa-mir-935;hsa-mir-96;

module 119 hsa-let-7a;hsa-mir-105-1;hsa-mir-105-2;hsa-mir-10b;hsa-mir-1179;hsa-mir-1180;hsa-mir-1181;hsa-mir-1183;hsa-mir-1184-1;hsa-mir-1202;hsa-mir-1207;hsa-mir-1231;hsa-mir-1234;hsa-mir-1236;hsa-mir-1247;hsa-mir-1249;hsa-mir-1254-1;hsa-mir-1258;hsa-mir-1266;hsa-mir-1271;hsa-mir-1273c;hsa-mir-1275;hsa-mir-1293;hsa-mir-1297;hsa-mir-1301;hsa-mir-1303;hsa-mir-134;hsa-mir-135a-1;hsa-mir-135a-2;hsa-mir-136;hsa-mir-139;hsa-mir-1471;hsa-mir-147a;hsa-mir-148b;hsa-mir-151;hsa-mir-1827;hsa-mir-185;hsa-mir-186;hsa-mir-189;hsa-mir-18b;hsa-mir-1909;hsa-mir-190a;hsa-mir-191;hsa-mir-1915;hsa-mir-197;hsa-mir-198;hsa-mir-202;hsa-mir-2110;hsa-mir-216a;hsa-mir-216b;hsa-mir-217;hsa-mir-218-1;hsa-mir-218-2;hsa-mir-26;hsa-mir-26b;hsa-mir-27a;hsa-mir-297;hsa-mir-298;hsa-mir-29b;hsa-mir-301a;hsa-mir-3148;hsa-mir-320e;hsa-mir-323b;hsa-mir-324;hsa-mir-326;hsa-mir-330;hsa-mir-33a;hsa-mir-340;hsa-mir-345;hsa-mir-346;hsa-mir-361;hsa-mir-365a;hsa-mir-365b;hsa-mir-369;hsa-mir-371;hsa-mir-376a-1;hsa-mir-376a-2;hsa-mir-376b;hsa-mir-377;hsa-mir-379;hsa-mir-381;hsa-mir-3940;hsa-mir-409;hsa-mir-423;hsa-mir-432;hsa-mir-450a-1;hsa-mir-450a-2;hsa-mir-450b;hsa-mir-451;hsa-mir-4792;hsa-mir-483;hsa-mir-485;hsa-mir-487a;hsa-mir-487b;hsa-mir-489;hsa-mir-490;hsa-mir-492;hsa-mir-493;hsa-mir-499b;hsa-mir-500a;hsa-mir-500b;hsa-mir-502;hsa-mir-509;hsa-mir-511-1;hsa-mir-511-2;hsa-mir-512-1;hsa-mir-512-2;hsa-mir-515-1;hsa-mir-515-2;hsa-mir-517c;hsa-mir-519d;hsa-mir-519e;hsa-mir-522;hsa-mir-523;hsa-mir-525;hsa-mir-526a-1;hsa-mir-526a-2;hsa-mir-526b;hsa-mir-527;hsa-mir-542;hsa-mir-545;hsa-mir-548a-1;hsa-mir-548a-2;hsa-mir-548a-3;hsa-mir-548c;hsa-mir-550a-3;hsa-mir-552;hsa-mir-564;hsa-mir-567;hsa-mir-569;hsa-mir-602;hsa-mir-608;hsa-mir-612;hsa-mir-615;hsa-mir-624;hsa-mir-629;hsa-mir-632;hsa-mir-636;hsa-mir-637;hsa-mir-638;hsa-mir-650;hsa-mir-657;hsa-mir-659;hsa-mir-660;hsa-mir-668;hsa-mir-711;hsa-mir-765;hsa-mir-92;hsa-mir-92b;hsa-mir-941-1;hsa-mir-941-3;hsa-mir-941-4;hsa-mir-95;

module 120 hsa-mir-1224;hsa-mir-138-1;hsa-mir-138-2;hsa-mir-149;hsa-mir-185;hsa-mir-196b;hsa-mir-199b;hsa-mir-224;hsa-mir-331;hsa-mir-339;hsa-mir-370;hsa-mir-371a;hsa-mir-378a;hsa-mir-451a;hsa-mir-454;hsa-mir-485;hsa-mir-498;hsa-mir-519c;hsa-mir-532;hsa-mir-542;hsa-mir-575;hsa-mir-638;hsa-mir-660;hsa-mir-663a;hsa-mir-663b;hsa-mir-708;hsa-mir-93;

module 121 hsa-mir-106a;hsa-mir-107;hsa-mir-122;hsa-mir-132;hsa-mir-15a;hsa-mir-15b;hsa-mir-16-1;hsa-mir-16-2;hsa-mir-181b-1;hsa-mir-191;hsa-mir-196a-1;hsa-mir-196a-2;hsa-mir-196b;hsa-mir-212;hsa-mir-23a;hsa-mir-24-2;hsa-mir-27a;hsa-mir-32;hsa-mir-362;hsa-mir-431;hsa-mir-532;

module 122 hsa-mir-153-2;hsa-mir-20b;hsa-mir-215;hsa-mir-30e;hsa-mir-339;hsa-mir-367;hsa-mir-383;hsa-mir-488;hsa-mir-506;hsa-mir-518e;hsa-mir-519b;hsa-mir-532;hsa-mir-621;hsa-mir-933;

module 123 hsa-let-7a-1;hsa-let-7a-2;hsa-let-7a-3;hsa-mir-154;hsa-mir-188;hsa-mir-23b;hsa-mir-299;hsa-mir-301a;hsa-mir-301b;hsa-mir-326;hsa-mir-337;hsa-mir-361;hsa-mir-376a-1;hsa-mir-376a-2;hsa-mir-376b;hsa-mir-376c;hsa-mir-409;hsa-mir-424;hsa-mir-449a;hsa-mir-449b;hsa-mir-491;hsa-mir-495;hsa-mir-675;hsa-mir-99b;

module 124

module 125 hsa-mir-29a;hsa-mir-29b-1;hsa-mir-29b-2;hsa-mir-29c;hsa-mir-30a;hsa-mir-30b;hsa-mir-30c-1;hsa-mir-30c-2;hsa-mir-30d;hsa-mir-431;hsa-mir-448;

module 126

module 127 hsa-mir-1256;hsa-mir-1296;hsa-mir-151b;hsa-mir-301b;hsa-mir-331;hsa-mir-488;hsa-mir-521-1;hsa-mir-521-2;hsa-mir-616;hsa-mir-647;

module 128

module 129 hsa-mir-103a-1;hsa-mir-103a-2;hsa-mir-103b-1;hsa-mir-103b-2;hsa-mir-128-1;hsa-mir-128-2;hsa-mir-130b;hsa-mir-152;hsa-mir-181a-1;hsa-mir-181a-2;hsa-mir-20b;hsa-mir-210;hsa-mir-26a-1;hsa-mir-26a-2;hsa-mir-298;hsa-mir-302a;hsa-mir-302b;hsa-mir-302c;hsa-mir-302d;hsa-mir-302e;hsa-mir-302f;hsa-mir-325;hsa-mir-342;hsa-mir-411;hsa-mir-455;hsa-mir-518c;hsa-mir-574;

module 130

module 131 hsa-mir-125b-1;hsa-mir-126;hsa-mir-143;hsa-mir-145;hsa-mir-181a-1;hsa-mir-181a-2;hsa-mir-181b-1;hsa-mir-181b-2;hsa-mir-181c;hsa-mir-20a;hsa-mir-210;hsa-mir-221;hsa-mir-222;hsa-mir-223;hsa-mir-24-1;hsa-mir-33a;hsa-mir-33b;hsa-mir-342;hsa-mir-34a;hsa-mir-411;hsa-mir-636;

module 132 hsa-mir-100;hsa-mir-101-1;hsa-mir-101-2;hsa-mir-1246;hsa-mir-1286;hsa-mir-129-2;hsa-mir-130a;hsa-mir-130b;hsa-mir-1322;hsa-mir-148a;hsa-mir-150;hsa-mir-192-2;hsa-mir-193a;hsa-mir-196a-1;hsa-mir-196a-2;hsa-mir-196b;hsa-mir-200c;hsa-mir-203;hsa-mir-205;hsa-mir-210;hsa-mir-22;hsa-mir-25;hsa-mir-27a;hsa-mir-28;hsa-mir-296;hsa-mir-302a;hsa-mir-302e;hsa-mir-302f;hsa-mir-30b;hsa-mir-31;hsa-mir-342;hsa-mir-34b;hsa-mir-34c;hsa-mir-375;hsa-mir-425;hsa-mir-451a;hsa-mir-452;hsa-mir-454;hsa-mir-455;hsa-mir-486;hsa-mir-499a;hsa-mir-518b;hsa-mir-519a-1;hsa-mir-519a-2;hsa-mir-519c;hsa-mir-548d-1;hsa-mir-548d-2;hsa-mir-574;hsa-mir-593;hsa-mir-596;hsa-mir-617;hsa-mir-655;hsa-mir-720;hsa-mir-744;hsa-mir-758;hsa-mir-766;hsa-mir-885;hsa-mir-98;hsa-mir-99a;hsa-mir-99b;

module 133 hsa-mir-100;hsa-mir-127;hsa-mir-130a;hsa-mir-140;hsa-mir-144;hsa-mir-148a;hsa-mir-154;hsa-mir-188;hsa-mir-203;hsa-mir-20b;hsa-mir-23b;hsa-mir-299;hsa-mir-301a;hsa-mir-301b;hsa-mir-320a;hsa-mir-323a;hsa-mir-335;hsa-mir-337;hsa-mir-361;hsa-mir-375;hsa-mir-376a-1;hsa-mir-376a-2;hsa-mir-376b;hsa-mir-376c;hsa-mir-379;hsa-mir-409;hsa-mir-449a;hsa-mir-449b;hsa-mir-452;hsa-mir-483;hsa-mir-484;hsa-mir-487b;hsa-mir-491;hsa-mir-495;hsa-mir-501;hsa-mir-675;hsa-mir-935;hsa-mir-99b;

module 134 hsa-mir-1256;hsa-mir-1296;hsa-mir-130a;hsa-mir-130b;hsa-mir-146b;hsa-mir-148a;hsa-mir-148b;hsa-mir-152;hsa-mir-153-1;hsa-mir-153-2;hsa-mir-191;hsa-mir-194-1;hsa-mir-194-2;hsa-mir-215;hsa-mir-383;hsa-mir-488;hsa-mir-502;hsa-mir-521-1;hsa-mir-521-2;hsa-mir-616;hsa-mir-625;hsa-mir-629;hsa-mir-642a;hsa-mir-647;hsa-mir-99b;

module 135 hsa-mir-132;hsa-mir-136;hsa-mir-139;hsa-mir-142;hsa-mir-150;hsa-mir-186;hsa-mir-197;hsa-mir-204;hsa-mir-208a;hsa-mir-208b;hsa-mir-211;hsa-mir-212;hsa-mir-216a;hsa-mir-26b;hsa-mir-296;hsa-mir-297;hsa-mir-300;hsa-mir-32;hsa-mir-325;hsa-mir-328;hsa-mir-330;hsa-mir-339;hsa-mir-365a;hsa-mir-365b;hsa-mir-377;hsa-mir-382;hsa-mir-423;hsa-mir-424;hsa-mir-451a;hsa-mir-494;hsa-mir-499a;hsa-mir-500a;hsa-mir-503;hsa-mir-507;hsa-mir-508;hsa-mir-512-1;hsa-mir-512-2;hsa-mir-523;hsa-mir-525;hsa-mir-526b;hsa-mir-637;hsa-mir-98;

module 136 hsa-mir-216b;

module 137 hsa-mir-105-1;hsa-mir-105-2;hsa-mir-106a;hsa-mir-107;hsa-mir-1180;hsa-mir-1181;hsa-mir-1184-1;hsa-mir-1207;hsa-mir-1227;hsa-mir-1229;hsa-mir-1236;hsa-mir-1247;hsa-mir-1249;hsa-mir-125a;hsa-mir-1266;hsa-mir-1280;hsa-mir-1297;hsa-mir-1299;hsa-mir-135b;hsa-mir-136;hsa-mir-137;hsa-mir-138-2;hsa-mir-149;hsa-mir-151;hsa-mir-153-2;hsa-mir-181a-1;hsa-mir-181a-2;hsa-mir-1827;hsa-mir-186;hsa-mir-18b;hsa-mir-191;hsa-mir-1915;hsa-mir-196a-1;hsa-mir-204;hsa-mir-20b;hsa-mir-211;hsa-mir-215;hsa-mir-216a;hsa-mir-217;hsa-mir-218-1;hsa-mir-219-1;hsa-mir-23b;hsa-mir-296;hsa-mir-298;hsa-mir-29b;hsa-mir-300;hsa-mir-302a;hsa-mir-302b;hsa-mir-302c;hsa-mir-302d;hsa-mir-30e;hsa-mir-3148;hsa-mir-320a;hsa-mir-328;hsa-mir-330;hsa-mir-338;hsa-mir-339;hsa-mir-342;hsa-mir-361;hsa-mir-362;hsa-mir-365a;hsa-mir-365b;hsa-mir-367;hsa-mir-369;hsa-mir-378a;hsa-mir-383;hsa-mir-422a;hsa-mir-432;hsa-mir-452;hsa-mir-4792;hsa-mir-487a;hsa-mir-488;hsa-mir-490;hsa-mir-501;hsa-mir-506;hsa-mir-507;hsa-mir-508;hsa-mir-509-1;hsa-mir-509-2;hsa-mir-509-3;hsa-mir-510;hsa-mir-511-1;hsa-mir-511-2;hsa-mir-513a-1;hsa-mir-513a-2;hsa-mir-513b;hsa-mir-513c;hsa-mir-514a-1;hsa-mir-514a-2;hsa-mir-514a-3;hsa-mir-517a;hsa-mir-518a-1;hsa-mir-518a-2;hsa-mir-518e;hsa-mir-519b;hsa-mir-526b;hsa-mir-532;hsa-mir-550a-1;hsa-mir-550a-2;hsa-mir-550a-3;hsa-mir-564;hsa-mir-571;hsa-mir-584;hsa-mir-592;hsa-mir-593;hsa-mir-621;hsa-mir-625;hsa-mir-646;hsa-mir-650;hsa-mir-660;hsa-mir-663;hsa-mir-664;hsa-mir-711;hsa-mir-767;hsa-mir-933;hsa-mir-941-1;hsa-mir-941-3;hsa-mir-941-4;hsa-mir-944;

module 138 hsa-mir-124-1;hsa-mir-124-2;hsa-mir-124-3;hsa-mir-128-2;hsa-mir-152;hsa-mir-153-1;hsa-mir-153-2;hsa-mir-298;hsa-mir-362;hsa-mir-487b;hsa-mir-494;hsa-mir-497;hsa-mir-632;hsa-mir-636;

module 139 hsa-mir-138-2;

module 140

module 141 hsa-mir-100;hsa-mir-1286;hsa-mir-1322;hsa-mir-192-2;hsa-mir-196a-1;hsa-mir-196a-2;hsa-mir-196b;hsa-mir-203;hsa-mir-548d-1;hsa-mir-548d-2;hsa-mir-617;hsa-mir-720;hsa-mir-758;hsa-mir-766;hsa-mir-99a;hsa-mir-99b;

module 142 hsa-mir-99b;

module 143 hsa-mir-100;hsa-mir-101-1;hsa-mir-101-2;hsa-mir-106b;hsa-mir-10b;hsa-mir-122;hsa-mir-1233-1;hsa-mir-1233-2;hsa-mir-124-1;hsa-mir-1246;hsa-mir-124a-2;hsa-mir-124a-3;hsa-mir-127;hsa-mir-1285-1;hsa-mir-1285-2;hsa-mir-129-1;hsa-mir-129-2;hsa-mir-1290;hsa-mir-130b;hsa-mir-134;hsa-mir-135a-1;hsa-mir-135a-2;hsa-mir-138-1;hsa-mir-138-2;hsa-mir-139;hsa-mir-141;hsa-mir-142;hsa-mir-144;hsa-mir-150;hsa-mir-151a;hsa-mir-151b;hsa-mir-184;hsa-mir-196a-1;hsa-mir-199b;hsa-mir-200a;hsa-mir-200c;hsa-mir-203;hsa-mir-204;hsa-mir-205;hsa-mir-206;hsa-mir-215;hsa-mir-218-1;hsa-mir-218-2;hsa-mir-219-1;hsa-mir-219-2;hsa-mir-224;hsa-mir-23b;hsa-mir-26b;hsa-mir-27a;hsa-mir-302b;hsa-mir-302c;hsa-mir-30c-1;hsa-mir-30c-2;hsa-mir-30d;hsa-mir-320a;hsa-mir-320b-1;hsa-mir-320b-2;hsa-mir-320c-1;hsa-mir-320d-1;hsa-mir-330;hsa-mir-335;hsa-mir-339;hsa-mir-34b;hsa-mir-34c;hsa-mir-363;hsa-mir-365a;hsa-mir-365b;hsa-mir-367;hsa-mir-378a;hsa-mir-378b;hsa-mir-378c;hsa-mir-378d-1;hsa-mir-378d-2;hsa-mir-378e;hsa-mir-378f;hsa-mir-378g;hsa-mir-378h;hsa-mir-378i;hsa-mir-381;hsa-mir-425;hsa-mir-429;hsa-mir-448;hsa-mir-451a;hsa-mir-452;hsa-mir-454;hsa-mir-508;hsa-mir-509-1;hsa-mir-509-2;hsa-mir-509-3;hsa-mir-514a-1;hsa-mir-514a-2;hsa-mir-514a-3;hsa-mir-571;hsa-mir-592;hsa-mir-625;hsa-mir-629;hsa-mir-655;hsa-mir-663;hsa-mir-708;hsa-mir-760;hsa-mir-93;hsa-mir-99a;hsa-mir-99b;

module 144 hsa-mir-1224;hsa-mir-137;hsa-mir-147a;hsa-mir-151a;hsa-mir-151b;hsa-mir-199b;hsa-mir-205;hsa-mir-22;hsa-mir-28;hsa-mir-301a;hsa-mir-335;hsa-mir-34b;hsa-mir-34c;hsa-mir-370;hsa-mir-371a;hsa-mir-372;hsa-mir-373;hsa-mir-374a;hsa-mir-375;hsa-mir-498;hsa-mir-663b;hsa-mir-7-1;hsa-mir-7-2;hsa-mir-7-3;

module 145 hsa-mir-191;hsa-mir-532;hsa-mir-663b;

module 146

module 147

module 148

module 149 hsa-mir-10a;hsa-mir-138-1;hsa-mir-144;hsa-mir-154;hsa-mir-193a;hsa-mir-193b;hsa-mir-196b;hsa-mir-199b;hsa-mir-224;hsa-mir-299;hsa-mir-30c-1;hsa-mir-3151;hsa-mir-323a;hsa-mir-323b;hsa-mir-328;hsa-mir-337;hsa-mir-369;hsa-mir-370;hsa-mir-382;hsa-mir-410;hsa-mir-424;hsa-mir-451a;hsa-mir-451b;hsa-mir-564;hsa-mir-575;hsa-mir-590;hsa-mir-628;hsa-mir-663a;

module 150 hsa-mir-101-1;hsa-mir-101-2;hsa-mir-10b;hsa-mir-138-1;hsa-mir-138-2;hsa-mir-144;hsa-mir-146a;hsa-mir-149;hsa-mir-151a;hsa-mir-151b;hsa-mir-205;hsa-mir-214;hsa-mir-216b;hsa-mir-218-2;hsa-mir-328;hsa-mir-34b;hsa-mir-663a;hsa-mir-9-1;hsa-mir-9-2;hsa-mir-9-3;

module 151

module 152

module 153 hsa-mir-132;

module 154

module 155

module 156

module 157 hsa-let-7a;hsa-mir-105-1;hsa-mir-105-2;hsa-mir-1179;hsa-mir-1180;hsa-mir-1181;hsa-mir-1183;hsa-mir-1184-1;hsa-mir-1202;hsa-mir-1207;hsa-mir-1224;hsa-mir-1227;hsa-mir-1229;hsa-mir-1233-1;hsa-mir-1233-2;hsa-mir-1236;hsa-mir-1246;hsa-mir-1247;hsa-mir-1249;hsa-mir-124a-2;hsa-mir-124a-3;hsa-mir-1254-1;hsa-mir-1256;hsa-mir-1258;hsa-mir-1260a;hsa-mir-1266;hsa-mir-1271;hsa-mir-1273a;hsa-mir-1273c;hsa-mir-1275;hsa-mir-128;hsa-mir-1280;hsa-mir-1285-1;hsa-mir-1285-2;hsa-mir-1286;hsa-mir-1293;hsa-mir-1296;hsa-mir-1297;hsa-mir-1299;hsa-mir-1303;hsa-mir-1305;hsa-mir-1322;hsa-mir-136;hsa-mir-1471;hsa-mir-147b;hsa-mir-151;hsa-mir-154;hsa-mir-1827;hsa-mir-188;hsa-mir-189;hsa-mir-1909;hsa-mir-190a;hsa-mir-190b;hsa-mir-1915;hsa-mir-192-2;hsa-mir-1972-1;hsa-mir-200;hsa-mir-202;hsa-mir-2110;hsa-mir-216b;hsa-mir-217;hsa-mir-2861;hsa-mir-297;hsa-mir-298;hsa-mir-29b;hsa-mir-300;hsa-mir-3148;hsa-mir-3151;hsa-mir-3163;hsa-mir-3179-1;hsa-mir-3179-2;hsa-mir-3179-3;hsa-mir-3196;hsa-mir-320d-2;hsa-mir-320e;hsa-mir-323a;hsa-mir-323b;hsa-mir-325;hsa-mir-329-1;hsa-mir-329-2;hsa-mir-331;hsa-mir-337;hsa-mir-33a;hsa-mir-33b;hsa-mir-369;hsa-mir-374a;hsa-mir-376b;hsa-mir-377;hsa-mir-379;hsa-mir-382;hsa-mir-384;hsa-mir-3940;hsa-mir-410;hsa-mir-411;hsa-mir-421;hsa-mir-422a;hsa-mir-431;hsa-mir-448;hsa-mir-450a-1;hsa-mir-450a-2;hsa-mir-450b;hsa-mir-451;hsa-mir-451b;hsa-mir-454;hsa-mir-455;hsa-mir-4792;hsa-mir-484;hsa-mir-487a;hsa-mir-487b;hsa-mir-489;hsa-mir-490;hsa-mir-492;hsa-mir-493;hsa-mir-498;hsa-mir-499b;hsa-mir-500a;hsa-mir-501;hsa-mir-502;hsa-mir-504;hsa-mir-505;hsa-mir-506;hsa-mir-507;hsa-mir-509;hsa-mir-510;hsa-mir-511-1;hsa-mir-511-2;hsa-mir-512-1;hsa-mir-512-2;hsa-mir-516a-1;hsa-mir-516a-2;hsa-mir-516b-1;hsa-mir-516b-2;hsa-mir-517a;hsa-mir-518a-1;hsa-mir-518a-2;hsa-mir-518b;hsa-mir-518c;hsa-mir-518e;hsa-mir-519a-1;hsa-mir-519a-2;hsa-mir-519b;hsa-mir-519c;hsa-mir-519d;hsa-mir-519e;hsa-mir-520f;hsa-mir-520g;hsa-mir-521-1;hsa-mir-521-2;hsa-mir-523;hsa-mir-525;hsa-mir-526b;hsa-mir-539;hsa-mir-544a;hsa-mir-544b;hsa-mir-545;hsa-mir-548a-1;hsa-mir-548a-2;hsa-mir-548a-3;hsa-mir-548b;hsa-mir-548c;hsa-mir-548d-1;hsa-mir-548d-2;hsa-mir-550a-3;hsa-mir-550b-1;hsa-mir-550b-2;hsa-mir-552;hsa-mir-557;hsa-mir-561;hsa-mir-564;hsa-mir-566;hsa-mir-567;hsa-mir-569;hsa-mir-571;hsa-mir-572;hsa-mir-575;hsa-mir-582;hsa-mir-583;hsa-mir-584;hsa-mir-589;hsa-mir-590;hsa-mir-592;hsa-mir-593;hsa-mir-596;hsa-mir-598;hsa-mir-599;hsa-mir-600;hsa-mir-601;hsa-mir-602;hsa-mir-603;hsa-mir-611;hsa-mir-612;hsa-mir-614;hsa-mir-615;hsa-mir-616;hsa-mir-617;hsa-mir-619;hsa-mir-621;hsa-mir-622;hsa-mir-624;hsa-mir-627;hsa-mir-628;hsa-mir-630;hsa-mir-632;hsa-mir-633;hsa-mir-635;hsa-mir-636;hsa-mir-637;hsa-mir-640;hsa-mir-642a;hsa-mir-642b;hsa-mir-646;hsa-mir-647;hsa-mir-648;hsa-mir-652;hsa-mir-654;hsa-mir-655;hsa-mir-657;hsa-mir-658;hsa-mir-659;hsa-mir-660;hsa-mir-661;hsa-mir-662;hsa-mir-663;hsa-mir-663b;hsa-mir-664;hsa-mir-668;hsa-mir-671;hsa-mir-675;hsa-mir-711;hsa-mir-720;hsa-mir-744;hsa-mir-758;hsa-mir-760;hsa-mir-765;hsa-mir-766;hsa-mir-767;hsa-mir-769;hsa-mir-873;hsa-mir-874;hsa-mir-885;hsa-mir-922;hsa-mir-933;hsa-mir-935;hsa-mir-941-1;hsa-mir-941-3;hsa-mir-941-4;hsa-mir-942;hsa-mir-944;

module 158 hsa-mir-190a;hsa-mir-216b;

module 159 hsa-mir-125b-1;hsa-mir-126;hsa-mir-129-1;hsa-mir-143;hsa-mir-145;hsa-mir-146a;hsa-mir-146b;hsa-mir-148b;hsa-mir-155;hsa-mir-21;hsa-mir-221;hsa-mir-222;hsa-mir-223;hsa-mir-27b;hsa-mir-34a;hsa-mir-486;hsa-mir-494;hsa-mir-503;hsa-mir-941-1;hsa-mir-941-3;hsa-mir-941-4;

module 160

module 161

module 162 hsa-mir-1-1;hsa-mir-1-2;hsa-mir-122;hsa-mir-130a;hsa-mir-133b;hsa-mir-134;hsa-mir-181a-1;hsa-mir-181a-2;hsa-mir-196a-2;hsa-mir-198;hsa-mir-222;hsa-mir-340;hsa-mir-346;hsa-mir-370;hsa-mir-381;hsa-mir-499a;hsa-mir-624;

module 163

module 164 hsa-mir-302e;hsa-mir-302f;hsa-mir-455;hsa-mir-744;hsa-mir-885;

module 165 hsa-mir-103b-1;hsa-mir-103b-2;hsa-mir-186;hsa-mir-190a;hsa-mir-216a;hsa-mir-216b;hsa-mir-32;hsa-mir-330;hsa-mir-625;hsa-mir-630;hsa-mir-95;

module 166

module 167 hsa-let-7a-1;hsa-let-7a-2;hsa-let-7a-3;hsa-let-7b;hsa-let-7c;hsa-let-7d;hsa-let-7e;hsa-let-7f-1;hsa-let-7f-2;hsa-let-7g;hsa-let-7i;hsa-mir-10a;hsa-mir-127;hsa-mir-143;hsa-mir-154;hsa-mir-181b-1;hsa-mir-181b-2;hsa-mir-181c;hsa-mir-181d;hsa-mir-196b;hsa-mir-219-1;hsa-mir-219-2;hsa-mir-24-1;hsa-mir-26a-1;hsa-mir-26a-2;hsa-mir-299;hsa-mir-3151;hsa-mir-323a;hsa-mir-337;hsa-mir-342;hsa-mir-370;hsa-mir-382;hsa-mir-424;hsa-mir-590;hsa-mir-628;hsa-mir-98;

module 168

module 169 hsa-mir-1256;hsa-mir-1296;hsa-mir-151b;hsa-mir-488;hsa-mir-521-1;hsa-mir-521-2;hsa-mir-616;hsa-mir-647;

module 170 hsa-mir-101-1;hsa-mir-105-1;hsa-mir-105-2;hsa-mir-128-1;hsa-mir-128-2;hsa-mir-137;hsa-mir-138-1;hsa-mir-138-2;hsa-mir-144;hsa-mir-147b;hsa-mir-195;hsa-mir-218-2;hsa-mir-26b;hsa-mir-27b;hsa-mir-421;hsa-mir-9-1;hsa-mir-9-2;hsa-mir-9-3;hsa-mir-942;

module 171 hsa-let-7a;hsa-mir-1254-1;hsa-mir-1258;hsa-mir-134;hsa-mir-149;hsa-mir-151b;hsa-mir-198;hsa-mir-212;hsa-mir-328;hsa-mir-337;hsa-mir-361;hsa-mir-374a;hsa-mir-3940;hsa-mir-451a;hsa-mir-451b;hsa-mir-574;

module 172 hsa-mir-106b;hsa-mir-126;hsa-mir-132;hsa-mir-142;hsa-mir-144;hsa-mir-146a;hsa-mir-146b;hsa-mir-155;hsa-mir-193a;hsa-mir-193b;hsa-mir-197;hsa-mir-203;hsa-mir-210;hsa-mir-211;hsa-mir-212;hsa-mir-222;hsa-mir-223;hsa-mir-30a;hsa-mir-31;hsa-mir-328;hsa-mir-339;hsa-mir-365a;hsa-mir-369;hsa-mir-375;hsa-mir-410;hsa-mir-423;hsa-mir-451a;hsa-mir-451b;hsa-mir-486;hsa-mir-499a;hsa-mir-519b;hsa-mir-564;hsa-mir-592;hsa-mir-615;hsa-mir-663a;hsa-mir-675;

module 173 hsa-mir-122;hsa-mir-141;hsa-mir-149;hsa-mir-183;hsa-mir-18b;hsa-mir-192;hsa-mir-200a;hsa-mir-200b;hsa-mir-200c;hsa-mir-205;hsa-mir-28;hsa-mir-302a;hsa-mir-302b;hsa-mir-370;hsa-mir-371a;hsa-mir-372;hsa-mir-373;hsa-mir-429;hsa-mir-491;hsa-mir-501;hsa-mir-583;hsa-mir-602;hsa-mir-663;

module 174 hsa-mir-100;hsa-mir-137;hsa-mir-15b;hsa-mir-194-1;hsa-mir-194-2;hsa-mir-196a-1;hsa-mir-196a-2;hsa-mir-200a;hsa-mir-200c;hsa-mir-203;hsa-mir-205;hsa-mir-214;hsa-mir-28;hsa-mir-301a;hsa-mir-338;hsa-mir-345;hsa-mir-34b;hsa-mir-34c;hsa-mir-375;hsa-mir-376b;hsa-mir-424;hsa-mir-451a;hsa-mir-491;hsa-mir-494;hsa-mir-497;hsa-mir-499a;hsa-mir-512-1;hsa-mir-512-2;hsa-mir-518a-1;hsa-mir-518a-2;hsa-mir-7-1;hsa-mir-7-2;hsa-mir-7-3;

module 175 hsa-mir-150;hsa-mir-331;hsa-mir-339;hsa-mir-378a;hsa-mir-454;hsa-mir-485;hsa-mir-575;hsa-mir-660;

module 176 hsa-mir-1-1;hsa-mir-1-2;hsa-mir-431;hsa-mir-448;hsa-mir-491;hsa-mir-501;hsa-mir-583;hsa-mir-602;hsa-mir-663;

module 177 hsa-mir-100;hsa-mir-10a;hsa-mir-1224;hsa-mir-1227;hsa-mir-1229;hsa-mir-127;hsa-mir-1280;hsa-mir-135b;hsa-mir-196b;hsa-mir-212;hsa-mir-224;hsa-mir-296;hsa-mir-300;hsa-mir-328;hsa-mir-449a;hsa-mir-449b;hsa-mir-452;hsa-mir-493;hsa-mir-503;hsa-mir-517a;hsa-mir-574;hsa-mir-642a;hsa-mir-642b;hsa-mir-708;hsa-mir-944;hsa-mir-99a;hsa-mir-99b;

module 178 hsa-mir-125a;hsa-mir-181a-1;hsa-mir-181a-2;hsa-mir-181b-1;hsa-mir-181b-2;hsa-mir-181c;hsa-mir-181d;hsa-mir-18b;hsa-mir-196b;hsa-mir-219-2;hsa-mir-24-1;hsa-mir-2861;hsa-mir-3196;hsa-mir-378a;hsa-mir-489;hsa-mir-542;hsa-mir-632;hsa-mir-636;hsa-mir-671;hsa-mir-720;hsa-mir-874;

module 179 hsa-mir-105-1;hsa-mir-105-2;hsa-mir-126;hsa-mir-143;hsa-mir-145;hsa-mir-147b;hsa-mir-150;hsa-mir-188;hsa-mir-195;hsa-mir-199a-1;hsa-mir-199a-2;hsa-mir-199b;hsa-mir-21;hsa-mir-214;hsa-mir-221;hsa-mir-222;hsa-mir-28;hsa-mir-320c-1;hsa-mir-320c-2;hsa-mir-331;hsa-mir-34a;hsa-mir-34c;hsa-mir-378a;hsa-mir-454;hsa-mir-571;hsa-mir-575;hsa-mir-652;hsa-mir-660;hsa-mir-9-1;hsa-mir-9-2;hsa-mir-9-3;hsa-mir-942;

module 180 hsa-mir-106a;hsa-mir-106b;hsa-mir-128-1;hsa-mir-128-2;hsa-mir-17;hsa-mir-18a;hsa-mir-19a;hsa-mir-19b-1;hsa-mir-19b-2;hsa-mir-20a;hsa-mir-20b;hsa-mir-30e;hsa-mir-92a-1;hsa-mir-92a-2;hsa-mir-93;

module 181

module 182 hsa-mir-10a;hsa-mir-138-1;hsa-mir-138-2;hsa-mir-410;hsa-mir-885;

module 183 hsa-mir-125b-1;hsa-mir-125b-2;hsa-mir-126;hsa-mir-143;hsa-mir-145;hsa-mir-146a;hsa-mir-15a;hsa-mir-17;hsa-mir-181a-1;hsa-mir-181a-2;hsa-mir-181b-1;hsa-mir-181b-2;hsa-mir-181c;hsa-mir-181d;hsa-mir-18a;hsa-mir-196b;hsa-mir-19a;hsa-mir-20a;hsa-mir-210;hsa-mir-221;hsa-mir-222;hsa-mir-223;hsa-mir-24-1;hsa-mir-342;hsa-mir-34a;hsa-mir-636;hsa-mir-640;

module 184

module 185

module 186

module 187 hsa-mir-1224;hsa-mir-124-1;hsa-mir-124-2;hsa-mir-124-3;hsa-mir-125a;hsa-mir-128-1;hsa-mir-128-2;hsa-mir-137;hsa-mir-1471;hsa-mir-148a;hsa-mir-152;hsa-mir-153-1;hsa-mir-153-2;hsa-mir-188;hsa-mir-1909;hsa-mir-383;hsa-mir-483;hsa-mir-488;hsa-mir-494;hsa-mir-497;hsa-mir-519c;hsa-mir-561;hsa-mir-590;hsa-mir-622;hsa-mir-630;hsa-mir-671;hsa-mir-720;hsa-mir-765;

module 188 hsa-mir-216b;

module 189

module 190

module 191 hsa-mir-105-1;hsa-mir-105-2;hsa-mir-1180;hsa-mir-1202;hsa-mir-1224;hsa-mir-1231;hsa-mir-1233-1;hsa-mir-1233-2;hsa-mir-1234;hsa-mir-1236;hsa-mir-1249;hsa-mir-124a-2;hsa-mir-124a-3;hsa-mir-1258;hsa-mir-1271;hsa-mir-1285-1;hsa-mir-1285-2;hsa-mir-1290;hsa-mir-1293;hsa-mir-1301;hsa-mir-1303;hsa-mir-1471;hsa-mir-147a;hsa-mir-148b;hsa-mir-187;hsa-mir-189;hsa-mir-190a;hsa-mir-1915;hsa-mir-197;hsa-mir-199a-2;hsa-mir-200;hsa-mir-202;hsa-mir-208b;hsa-mir-2110;hsa-mir-217;hsa-mir-26;hsa-mir-298;hsa-mir-301b;hsa-mir-3179-1;hsa-mir-3179-2;hsa-mir-3179-3;hsa-mir-320a;hsa-mir-320b-1;hsa-mir-320b-2;hsa-mir-320c-1;hsa-mir-320c-2;hsa-mir-320d-1;hsa-mir-320d-2;hsa-mir-320e;hsa-mir-323b;hsa-mir-324;hsa-mir-328;hsa-mir-340;hsa-mir-345;hsa-mir-346;hsa-mir-362;hsa-mir-365a;hsa-mir-365b;hsa-mir-371;hsa-mir-379;hsa-mir-409;hsa-mir-411;hsa-mir-423;hsa-mir-425;hsa-mir-449b;hsa-mir-450a-1;hsa-mir-450a-2;hsa-mir-450b;hsa-mir-484;hsa-mir-489;hsa-mir-490;hsa-mir-492;hsa-mir-493;hsa-mir-498;hsa-mir-499b;hsa-mir-500a;hsa-mir-500b;hsa-mir-502;hsa-mir-503;hsa-mir-505;hsa-mir-506;hsa-mir-508;hsa-mir-510;hsa-mir-512-1;hsa-mir-512-2;hsa-mir-515-1;hsa-mir-515-2;hsa-mir-517a;hsa-mir-517c;hsa-mir-518b;hsa-mir-519c;hsa-mir-519d;hsa-mir-519e;hsa-mir-522;hsa-mir-526a-1;hsa-mir-526a-2;hsa-mir-526b;hsa-mir-527;hsa-mir-532;hsa-mir-545;hsa-mir-550a-3;hsa-mir-550b-1;hsa-mir-550b-2;hsa-mir-571;hsa-mir-584;hsa-mir-590;hsa-mir-602;hsa-mir-608;hsa-mir-612;hsa-mir-615;hsa-mir-632;hsa-mir-636;hsa-mir-637;hsa-mir-638;hsa-mir-650;hsa-mir-655;hsa-mir-657;hsa-mir-661;hsa-mir-663a;hsa-mir-744;hsa-mir-765;hsa-mir-874;hsa-mir-92;hsa-mir-922;

module 192 hsa-mir-100;hsa-mir-101-1;hsa-mir-101-2;hsa-mir-126;hsa-mir-135b;hsa-mir-141;hsa-mir-142;hsa-mir-182;hsa-mir-183;hsa-mir-192;hsa-mir-196a-1;hsa-mir-196a-2;hsa-mir-200a;hsa-mir-200b;hsa-mir-200c;hsa-mir-203;hsa-mir-205;hsa-mir-214;hsa-mir-23b;hsa-mir-31;hsa-mir-3148;hsa-mir-375;hsa-mir-429;hsa-mir-569;hsa-mir-96;hsa-mir-99a;

module 193 hsa-let-7a;hsa-mir-134;hsa-mir-151b;hsa-mir-212;hsa-mir-374a;hsa-mir-449a;hsa-mir-485;hsa-mir-574;

module 194

module 195 hsa-mir-106b;hsa-mir-125b-1;hsa-mir-125b-2;hsa-mir-15a;hsa-mir-16-1;hsa-mir-17;hsa-mir-181a-1;hsa-mir-181a-2;hsa-mir-18a;hsa-mir-19a;hsa-mir-19b-1;hsa-mir-19b-2;hsa-mir-20a;hsa-mir-92a-1;hsa-mir-92a-2;

module 196 hsa-mir-15b;hsa-mir-196a-1;hsa-mir-196a-2;hsa-mir-28;hsa-mir-382;hsa-mir-424;hsa-mir-512-2;hsa-mir-518a-1;hsa-mir-518a-2;

module 197 hsa-mir-100;hsa-mir-125b-1;hsa-mir-125b-2;hsa-mir-126;hsa-mir-142;hsa-mir-143;hsa-mir-145;hsa-mir-146a;hsa-mir-146b;hsa-mir-150;hsa-mir-16-1;hsa-mir-17;hsa-mir-197;hsa-mir-203;hsa-mir-20a;hsa-mir-21;hsa-mir-221;hsa-mir-222;hsa-mir-31;hsa-mir-34a;hsa-mir-378a;hsa-mir-384;hsa-mir-422a;hsa-mir-423;hsa-mir-487a;hsa-mir-492;hsa-mir-671;hsa-mir-720;hsa-mir-99a;

module 198

module 199 hsa-mir-124-1;hsa-mir-124-2;hsa-mir-124-3;hsa-mir-184;hsa-mir-345;hsa-mir-363;hsa-mir-494;hsa-mir-497;hsa-mir-499a;

module 200 hsa-let-7a-1;hsa-let-7a-2;hsa-let-7a-3;hsa-let-7b;hsa-let-7c;hsa-let-7d;hsa-let-7e;hsa-let-7f-1;hsa-let-7f-2;hsa-let-7g;hsa-let-7i;hsa-mir-125a;hsa-mir-132;hsa-mir-143;hsa-mir-210;hsa-mir-221;hsa-mir-222;hsa-mir-98;
