# Supplementary material for: Predicting miRNA-Disease Association Based on Modularity Preserving Heterogeneous Network Embedding
Source: Front Cell Dev Biol. 2021 Jun 10;9:603758. doi: 10.3389/fcell.2021.603758 (PMC8223753; doi:10.3389/fcell.2021.603758)
Supplement: Supplementary file 4 [file Table_4.DOCX]

module 1 Anxiety Disorders;Asthma;Carotid Artery Diseases;Cataract;Cholesteatoma;Digestive System Neoplasms;Gastrointestinal Neoplasms;Hyperglycemia;Hypertrophy;Inflammation;Influenza, Human;Liposarcoma;Marek Disease;Mycosis Fungoides;Myelodysplastic Syndromes;Nevus, Pigmented;Obesity;Pituitary Neoplasms;Sarcoma, Ewing's;Sarcoma, Kaposi;Supranuclear Palsy, Progressive;Sarcoma, Synovial;Precursor B-Cell Lymphoblastic Leukemia-Lymphoma;Atherosclerosis;Lymphoma, Primary Effusion;

module 2

module 3

module 4 Adenoma;Albuminuria;Alopecia;Alzheimer Disease;Angina, Unstable;Behcet Syndrome;Carcinoma;Cardiomyopathy, Hypertrophic;Cardiovascular Diseases;Carotid Artery Diseases;Chlamydia Infections;Cholesteatoma;Chondrodysplasia Punctata;Choriocarcinoma;Colitis, Ulcerative;Cryptosporidium;Dermatitis, Atopic;Dyspepsia;Eczema;Endometriosis;Endothelium, Vascular;Erythropoiesis;Esophagus;Giant Cell Tumors;Glomerulonephritis;Granulosa Cell Tumor;Huntington Disease;Hyperglycemia;Hypopharyngeal Neoplasms;Creutzfeldt-Jakob Syndrome;Laryngeal Neoplasms;Leiomyosarcoma;Leprosy;Leukemia, Myeloid;Leukoplakia, Oral;Lupus Erythematosus, Systemic;Marek Disease;Metabolic Diseases;Mouth Neoplasms;Myotonic Dystrophy;Nasal Polyps;Neuroma, Acoustic;Nevus, Pigmented;Obesity;Osteolysis;Periodontal Diseases;Polycythemia Vera;Psoriasis;Pulmonary Fibrosis;Radiation Injuries;Rectal Neoplasms;Sarcoma;Sarcoma, Ewing's;Osteosarcoma;Schistosomiasis;Scleroderma, Systemic;Sjogren's Syndrome;Stomach Diseases;Supranuclear Palsy, Progressive;Prolactinoma;Myocardial Reperfusion Injury;Leukemia, B-Cell;Precursor B-Cell Lymphoblastic Leukemia-Lymphoma;Leukemia, Biphenotypic, Acute;Leukemia, Promyelocytic, Acute;HIV-1;AIDS Dementia Complex;HIV Infections;Retinal Neovascularization;Gerstmann-Straussler-Scheinker Disease;Lymphoma, T-Cell;Lymphoma, Large B-Cell, Diffuse;Pemphigus, Benign Familial;Myocardial Ischemia;Aortic Aneurysm, Abdominal;Lung Diseases, Interstitial;Focal Epithelial Hyperplasia;Lichen Planus, Oral;Hepatoblastoma;Carcinoma, Ductal, Breast;Carcinoma, Small Cell;Hematologic Neoplasms;Hypoxia-Ischemia, Brain;ACTH-Secreting Pituitary Adenoma;Dyslipidemias;Precursor Cell Lymphoblastic Leukemia-Lymphoma;Precursor T-Cell Lymphoblastic Leukemia-Lymphoma;Fatty Liver, Non-Alcoholic;

module 5 Lung Neoplasms;Colorectal Neoplasms;

module 6 Cerebral Infarction;Fanconi Anemia;Tourette Syndrome;Infertility, Male;Liver Cirrhosis;Lung Neoplasms;Retinoblastoma;Osteosarcoma;Scleroderma, Localized;Stomach Neoplasms;Leukemia, Promyelocytic, Acute;Neoplasms, Squamous Cell;Multiple Endocrine Neoplasia Type 1;Cocaine-Related Disorders;

module 7

module 8 Alzheimer Disease;Amyotrophic Lateral Sclerosis;Intracranial Aneurysm;Diabetes Mellitus, Type 2;Fanconi Anemia;Tourette Syndrome;Hodgkin Disease;Laryngeal Neoplasms;Lung Diseases;Mesothelioma;Obesity;Pain;Pre-Eclampsia;Schizophrenia;Supranuclear Palsy, Progressive;Synapses;Toxoplasmosis;Leukemia, lymphoblastic, Chronic;Leukemia, Promyelocytic, Acute;Lung Diseases, Interstitial;Precursor Cell Lymphoblastic Leukemia-Lymphoma;Small Cell Lung Carcinoma;

module 9 Anxiety Disorders;Breast Neoplasms;Carcinoma, Non-Small-Cell Lung;Endometriosis;Ependymoma;Fibrosarcoma;Gout;Heart Failure;Hemangioma;Neoplasms;Periodontitis;Pregnancy, Ectopic;Stomach Neoplasms;Neoplasms, Squamous Cell;SARS Virus;Azoospermia;

module 10 Head and Neck Neoplasms;Nasopharyngeal Neoplasms;

module 11 Colonic Neoplasms;Lung Neoplasms;Pancreatic Neoplasms;Colorectal Neoplasms;

module 12 Adenocarcinoma;Amyloidosis;Anus Neoplasms;Arthritis;Autoimmune Diseases;Carotid Artery Diseases;Uterine Cervical Neoplasms;Cryptosporidium;Eclampsia;Fatty Liver, Alcoholic;Ischemia;Kidney Neoplasms;Lymphoproliferative Disorders;Marek Disease;Multiple Myeloma;Muscular Dystrophies;Myositis Ossificans;Odontogenic Tumors;Sarcoma;Spinal Cord Injuries;Tongue Neoplasms;Reperfusion Injury;Leukemia, lymphoblastic, Chronic;Arthritis, Psoriatic;Panic Disorder;Liver Failure;Prion Diseases;Myocardial Ischemia;Adrenocortical Adenoma;Adrenocortical Carcinoma;Ischemic Preconditioning;Atherosclerosis;Acute Lung Injury;Drug-Induced Liver Injury;Kidney Failure, Acute;

module 13 Breast Neoplasms;Prostatic Neoplasms;Stomach Neoplasms;

module 14 Adenoma;Albuminuria;Alopecia;Arthritis;Autoimmune Diseases;Barrett Esophagus;Brain Injury;Carcinoma;Cardiomyopathy, Dilated;Cardiovascular Diseases;Cholesteatoma;Choriocarcinoma;Colitis, Ulcerative;Cryptosporidium;Demyelinating Diseases;Dermatitis, Atopic;Diabetes Mellitus, Type 2;Eclampsia;Esophagus;Fatty Liver, Alcoholic;Fibrosis;Tourette Syndrome;Granulosa Cell Tumor;Hamartoma Syndrome, Multiple;Heart Diseases;Hodgkin Disease;Hypopharyngeal Neoplasms;Kidney Neoplasms;Laryngeal Neoplasms;Leiomyoma;Leiomyosarcoma;Leprosy;Leukoplakia, Oral;Liver Neoplasms;Lung Diseases;Lymphoma, Non-Hodgkin;Metabolic Diseases;Cardiomyopathies;Neuroma, Acoustic;Neutropenia;Obesity;Osteolysis;Polycythemia Vera;Psoriasis;Pulmonary Fibrosis;Radiation Injuries;Salivary Gland Neoplasms;Sarcoma, Ewing's;Schistosomiasis;Sezary Syndrome;Spinal Cord Injuries;Vascular Diseases;Wounds and Injuries;Inflammatory Bowel Diseases;Myocardial Reperfusion Injury;AIDS Dementia Complex;Lymphoma, Large B-Cell, Diffuse;Myocardial Ischemia;Aortic Aneurysm, Abdominal;Focal Epithelial Hyperplasia;Lichen Planus, Oral;Carcinoma, Endometrioid;Carcinoma, Ductal, Breast;Carcinoma, Neuroendocrine;Carcinoma, Small Cell;Hypoxia-Ischemia, Brain;ACTH-Secreting Pituitary Adenoma;Acute Lung Injury;Fatty Liver, Non-Alcoholic;

module 15 Adenoviridae Infections;Anemia, Sickle Cell;Anxiety Disorders;Down Syndrome;Endomyocardial Fibrosis;Hand, Foot and Mouth Disease;Myositis Ossificans;Neurilemmoma;Pheochromocytoma;Lymphoma, T-Cell;Neurofibromatosis 2;Neurodegenerative Diseases;Dyslipidemias;Vascular Calcification;

module 16

module 17 Child Development Disorders, Pervasive;Fanconi Anemia;Graves Disease;Inflammation;Medulloblastoma;Meningioma;Nervous System Diseases;Osteosarcoma;Leukemia, Myeloid, Acute;Leukemia, Promyelocytic, Acute;Atherosclerosis;

module 18 Breast Neoplasms;

module 19 Aging;Anoxia;Breast Neoplasms;Carcinoma, Renal Cell;Colonic Neoplasms;Hand, Foot and Mouth Disease;Carcinoma, Hepatocellular;Liver Cirrhosis, Biliary;Lupus Nephritis;Myasthenia Gravis;Myopia;Pregnancy, Ectopic;Rhinitis, Allergic, Perennial;Sarcoma, Kaposi;Cystitis, Interstitial;

module 20 Urinary Bladder Neoplasms;Endometriosis;Multiple Myeloma;Reperfusion Injury;Atherosclerosis;

module 21

module 22

module 23 Alopecia;Alzheimer Disease;Amyotrophic Lateral Sclerosis;Intracranial Aneurysm;Diabetes Mellitus, Type 2;Endomyocardial Fibrosis;Huntington Disease;Kidney Failure, Chronic;Lung Neoplasms;Mesothelioma;Muscular Dystrophies;Myositis Ossificans;Myotonic Dystrophy;Nasal Polyps;Nevus, Pigmented;Obesity;Pain;Rhabdomyosarcoma;RNA Virus Infections;Sarcoma;Sjogren's Syndrome;Synapses;Prolactinoma;Leukemia, lymphoblastic, Chronic;Leukemia, Biphenotypic, Acute;Leukemia, Promyelocytic, Acute;HIV-1;Pemphigus, Benign Familial;Panic Disorder;Endometrial Neoplasms;Carcinoma, Endometrioid;Hematologic Neoplasms;Mastocytosis, Systemic;Dyslipidemias;Small Cell Lung Carcinoma;

module 24 Colonic Neoplasms;Lung Neoplasms;Pancreatic Neoplasms;Colorectal Neoplasms;

module 25 Leukemia, Myeloid, Acute;

module 26 Carcinoma, Hepatocellular;

module 27 Alzheimer Disease;Brain Neoplasms;Cerebral Infarction;Diabetes Mellitus, Type 1;Glioblastoma;Glioma;Hodgkin Disease;Intellectual Disability;Multiple Sclerosis;Neoplasms, Glandular and Epithelial;Neuroblastoma;Oligodendroglioma;Trophoblasts;Leukemia-Lymphoma, Adult T-Cell;Panic Disorder;Lung Diseases, Interstitial;Dyslipidemias;Precursor Cell Lymphoblastic Leukemia-Lymphoma;

module 28 Acquired Immunodeficiency Syndrome;Adrenal Cortex Neoplasms;Atrophy;Burns;Central Nervous System Diseases;Colonic Neoplasms;Ependymoma;Eye Abnormalities;Fragile X Syndrome;Carcinoma, Hepatocellular;Ischemia;Keratoconus;Liver Neoplasms;Lupus Vulgaris;Lupus Nephritis;Myocardial Infarction;Myocarditis;Osteoporosis;Psoriasis;Pulmonary Embolism;Sarcoma, Ewing's;Tongue Neoplasms;Leukemia, Myelogenous, Chronic, BCR-ABL Positive;Retinal Neovascularization;Gerstmann-Straussler-Scheinker Disease;Prion Diseases;Adrenocortical Adenoma;Ischemic Preconditioning;Precursor T-Cell Lymphoblastic Leukemia-Lymphoma;Frontotemporal Lobar Degeneration;Kidney Failure, Acute;

module 29 Carcinoma, Non-Small-Cell Lung;Fragile X Syndrome;Glioblastoma;Lung Neoplasms;Pulmonary Embolism;Trophoblasts;Mastocytosis, Systemic;

module 30 Atrophy;Carcinoma, Basal Cell;Carcinoma, Squamous Cell;Fanconi Anemia;Hepatitis B;Leukemia;Myelodysplastic Syndromes;Osteoarthritis;Osteosarcoma;Leukemia, Myeloid, Acute;Leukemia, Promyelocytic, Acute;Neoplasms, Squamous Cell;Cocaine-Related Disorders;Precursor Cell Lymphoblastic Leukemia-Lymphoma;

module 31

module 32 Alzheimer Disease;Anemia, Sickle Cell;Burns;Intracranial Aneurysm;Endomyocardial Fibrosis;Glomerulonephritis;HIV;Huntington Disease;Leukemia;Lupus Nephritis;Waldenstrom Macroglobulinemia;Myopia;Nasopharyngeal Neoplasms;Neoplasms;Pheochromocytoma;Thyroid Neoplasms;Leukemia, Myelogenous, Chronic, BCR-ABL Positive;HIV Infections;Panic Disorder;Lymphoma, Large-Cell, Anaplastic;Cervical Intraepithelial Neoplasia;Small Cell Lung Carcinoma;

module 33

module 34 Abortion, Habitual;Anus Neoplasms;Barrett Esophagus;Carcinoma, Non-Small-Cell Lung;Cardiomyopathy, Dilated;Intracranial Aneurysm;Cerebral Infarction;Uterine Cervical Neoplasms;Gastrointestinal Neoplasms;Hemangioma;Hepatitis, Chronic;Keloid;Intellectual Disability;Mesothelioma;Mouth Neoplasms;Moyamoya Disease;Neurilemmoma;Oligodendroglioma;Parkinson Disease;Rhinitis, Allergic, Perennial;Trophoblasts;Vitiligo;Reperfusion Injury;Retinal Neovascularization;Neurofibromatosis 2;Liver Failure;Hepatoblastoma;Adrenocortical Carcinoma;Pulmonary Disease, Chronic Obstructive;Azoospermia;Small Cell Lung Carcinoma;

module 35 Adenocarcinoma;Anxiety Disorders;Asthma;Ependymoma;Kidney Neoplasms;Multiple Myeloma;Muscular Dystrophies;Myositis Ossificans;Ovarian Neoplasms;Sarcoma, Synovial;Thyroid Neoplasms;Reperfusion Injury;Panic Disorder;Endometrial Neoplasms;Liver Failure;Atherosclerosis;Drug-Induced Liver Injury;

module 36 Head and Neck Neoplasms;

module 37 Carcinoma, Hepatocellular;Lung Neoplasms;

module 38 Anemia, Sickle Cell;Carcinoma, Renal Cell;Central Nervous System Diseases;Child Development Disorders, Pervasive;Ependymoma;Eye Abnormalities;Fragile X Syndrome;Infertility, Male;Keratoconus;Leiomyoma;Waldenstrom Macroglobulinemia;Meningioma;Myasthenia Gravis;Polycythemia Vera;Pulmonary Embolism;Tongue Neoplasms;Trophoblasts;Precursor B-Cell Lymphoblastic Leukemia-Lymphoma;Retinal Neovascularization;Cervical Intraepithelial Neoplasia;Nerve Sheath Neoplasms;Muscular Dystrophy, Facioscapulohumeral;Mastocytosis, Systemic;

module 39 Amyloidosis;Anemia, Sickle Cell;Anus Neoplasms;Arthritis;Arthritis, Rheumatoid;Autoimmune Diseases;Behcet Syndrome;Carotid Artery Diseases;Central Nervous System Diseases;Chlamydia Infections;Chondrodysplasia Punctata;Colitis;Colorectal Neoplasms, Hereditary Nonpolyposis;Coronary Artery Disease;Cystic Fibrosis;Diabetes Mellitus, Type 1;Down Syndrome;Dyspepsia;Eczema;Encephalomyelitis, Autoimmune, Experimental;Endothelium, Vascular;Erythropoiesis;Fatty Liver, Alcoholic;Fibroblasts;Francisella;Gastritis, Atrophic;Giant Cell Tumors;Glomerulonephritis;Graft vs Host Disease;Hepatitis;Hepatitis B;Huntington Disease;Hyperglycemia;Hyperlipidemias;Hypertension;Infertility, Male;Intervertebral Disk;Creutzfeldt-Jakob Syndrome;Liver Cirrhosis, Biliary;Liver Diseases;Lymphoproliferative Disorders;Marek Disease;Moyamoya Disease;Multiple Sclerosis;Myositis Ossificans;Obesity;Odontogenic Tumors;Osteoarthritis;Periodontal Diseases;Polycythemia Vera;Psychotic Disorders;Scleroderma, Systemic;Sjogren's Syndrome;Spinal Cord Injuries;Leukemia, lymphoblastic, Chronic;Arthritis, Psoriatic;HIV Infections;Gerstmann-Straussler-Scheinker Disease;Lymphoma, B-Cell;Panic Disorder;Liver Failure;Prion Diseases;Lichen Planus, Oral;Carcinoma, Neuroendocrine;Sepsis;Neurodegenerative Diseases;Hepatitis B, Chronic;Hepatitis C, Chronic;Stroke;Lymphoma, Mantle-Cell;Hypoxia-Ischemia, Brain;Diabetes Complications;ACTH-Secreting Pituitary Adenoma;Dyslipidemias;Atherosclerosis;Renal Insufficiency;Lipid Metabolism Disorders;Precursor T-Cell Lymphoblastic Leukemia-Lymphoma;Lymphoma, Extranodal NK-T-Cell;Acute Lung Injury;Drug-Induced Liver Injury;Fatty Liver, Non-Alcoholic;

module 40

module 41 Urinary Bladder Neoplasms;Carcinoma, Non-Small-Cell Lung;Central Nervous System Diseases;Coronary Artery Disease;Fragile X Syndrome;Glioblastoma;Liver Diseases, Alcoholic;Lung Neoplasms;Myopia;Pulmonary Embolism;Thyroid Neoplasms;Leukemia, Myelogenous, Chronic, BCR-ABL Positive;Cervical Intraepithelial Neoplasia;Neoplasms, Squamous Cell;Neurodegenerative Diseases;Cocaine-Related Disorders;

module 42

module 43 Anxiety Disorders;Carcinoma, Non-Small-Cell Lung;Colonic Neoplasms;Endometriosis;Ependymoma;Fibrosarcoma;Gout;Hemangioma;Sarcoma, Synovial;Retinal Neovascularization;Adrenocortical Carcinoma;SARS Virus;Precursor Cell Lymphoblastic Leukemia-Lymphoma;

module 44 Abortion, Habitual;Adenocarcinoma;Anus Neoplasms;Arthritis;Astrocytoma;Atrophy;Autoimmune Diseases;Barrett Esophagus;Cardiomyopathy, Dilated;Cerebral Infarction;Colitis, Ulcerative;Crohn Disease;Dementia;Diabetes Mellitus, Type 2;Erythropoiesis;Fatty Liver;Fatty Liver, Alcoholic;Tourette Syndrome;Glioma;Heart Diseases;Hepatitis;Hepatitis B;Hepatitis C;Hyperlipidemias;Keloid;Kidney Failure, Chronic;Liver Diseases, Alcoholic;Lymphoproliferative Disorders;Moyamoya Disease;Myasthenia Gravis;Odontogenic Tumors;Pancreatic Neoplasms;Pituitary Neoplasms;RNA Virus Infections;Spinal Cord Injuries;Supranuclear Palsy, Progressive;Tongue Neoplasms;Vascular Diseases;Vitiligo;Inflammatory Bowel Diseases;Reperfusion Injury;Arthritis, Psoriatic;Liver Failure;Myocardial Ischemia;Nerve Sheath Neoplasms;Hepatitis B, Chronic;Hepatitis C, Chronic;ACTH-Secreting Pituitary Adenoma;Lipid Metabolism Disorders;Acute Lung Injury;Drug-Induced Liver Injury;

module 45 Cerebral Infarction;Cholesteatoma;Diabetes Mellitus;Diabetes Mellitus, Type 1;Endometriosis;Ependymoma;Graves Disease;Hemangioma;Hepatitis C;Periodontitis;Retinoblastoma;Salivary Gland Neoplasms;Sarcoma, Synovial;Trophoblasts;Adrenocortical Carcinoma;

module 46 Astrocytoma;Head and Neck Neoplasms;

module 47

module 48 Carcinoma;Cerebellar Neoplasms;Uterine Cervical Neoplasms;Ependymoma;Fatty Liver;Fibrosarcoma;Glioma;Head and Neck Neoplasms;Hepatitis, Chronic;Kidney Failure, Chronic;Lupus Nephritis;Neoplasms;Neoplasms, Germ Cell and Embryonal;Pre-Eclampsia;Retinal Degeneration;Sarcoma, Ewing's;Skin Neoplasms;Testicular Neoplasms;Precursor B-Cell Lymphoblastic Leukemia-Lymphoma;Lung Diseases, Interstitial;Carcinoma, Embryonal;Cervical Intraepithelial Neoplasia;Nerve Sheath Neoplasms;Azoospermia;

module 49

module 50 Colonic Neoplasms;Endometriosis;

module 51 Hepatitis;Infertility, Male;Myeloproliferative Disorders;Parkinson Disease;Hepatitis C, Chronic;Lipid Metabolism Disorders;Frontotemporal Lobar Degeneration;

module 52 Adenocarcinoma;Colonic Neoplasms;Dementia;Diabetes Mellitus, Type 2;Fatty Liver;Tourette Syndrome;Hypertension;Kidney Failure, Chronic;Liver Diseases, Alcoholic;Lung Neoplasms;Myocardial Infarction;Pain;Pancreatic Neoplasms;Pre-Eclampsia;RNA Virus Infections;Schizophrenia;Supranuclear Palsy, Progressive;

module 53 Medulloblastoma;

module 54 Abortion, Habitual;Adrenal Cortex Neoplasms;Anus Neoplasms;Autistic Disorder;Carcinoma, Squamous Cell;Uterine Cervical Neoplasms;Dementia;Gastrointestinal Neoplasms;Giant Cell Tumors;Glioma;Head and Neck Neoplasms;Keloid;Kidney Failure, Chronic;Leiomyoma;Liver Diseases, Alcoholic;Lung Diseases;Intellectual Disability;Mesothelioma;Mouth Neoplasms;Neurilemmoma;Neuroblastoma;Obesity;Oligodendroglioma;Rhinitis, Allergic, Perennial;Schizophrenia;Supranuclear Palsy, Progressive;Thyroid Neoplasms;Vitiligo;Neurofibromatosis 2;Adrenocortical Adenoma;Adrenocortical Carcinoma;Ischemic Preconditioning;Pulmonary Disease, Chronic Obstructive;Dyslipidemias;Azoospermia;Small Cell Lung Carcinoma;Eosinophilic Esophagitis;Kidney Failure, Acute;

module 55 Crohn Disease;Endometriosis;Esophageal Neoplasms;

module 56 Acquired Immunodeficiency Syndrome;Alzheimer Disease;Amyotrophic Lateral Sclerosis;Anemia, Sickle Cell;Autistic Disorder;Biliary Atresia;Central Nervous System Diseases;Intracranial Aneurysm;Child Development Disorders, Pervasive;Cicatrix;Dementia;Diabetes Mellitus, Type 2;Fragile X Syndrome;Liver Diseases, Alcoholic;Lung Diseases;Lung Neoplasms;Waldenstrom Macroglobulinemia;Meningioma;Intellectual Disability;Mesothelioma;Myotonic Dystrophy;Neoplasms;Nervous System Diseases;Neurilemmoma;Obesity;Oligodendroglioma;Periodontal Diseases;Pulmonary Embolism;Rhabdomyosarcoma;Rhinitis, Allergic, Perennial;Schizophrenia;Scleroderma, Localized;Supranuclear Palsy, Progressive;Synapses;Leukemia, Myeloid, Chronic-Phase;Lymphoma, B-Cell;Neurofibromatosis 2;Aortic Aneurysm, Thoracic;Lung Diseases, Interstitial;Irritable Bowel Syndrome;Vascular Calcification;

module 57

module 58 Adenocarcinoma;Adenoma;Adrenal Cortex Neoplasms;Albuminuria;Amyotrophic Lateral Sclerosis;Anus Neoplasms;Aortic Valve Stenosis;Barrett Esophagus;Urinary Bladder Neoplasms;Brain Injury;Burns;Carcinoma;Carcinoma, Ehrlich Tumor;Cataract;Central Nervous System Diseases;Choriocarcinoma;Crohn Disease;Cryptosporidium;Diabetic Nephropathies;Diabetic Retinopathy;Endomyocardial Fibrosis;Erythropoiesis;Esophagus;Fatty Liver;Fibrosis;Gastrointestinal Neoplasms;Giant Cell Tumors;Glomerulonephritis;Glomerulonephritis, IGA;Granulosa Cell Tumor;Helplessness, Learned;Hepatitis B;Hodgkin Disease;Huntington Disease;Keloid;Kidney Neoplasms;Leiomyoma;Leiomyosarcoma;Leukoplakia, Oral;Liver Cirrhosis;Liver Neoplasms;Lupus Erythematosus, Systemic;Lymphoma;Meningioma;Mouth Neoplasms;Muscular Dystrophies;Nephrosclerosis;Pain;Pheochromocytoma;Pulmonary Fibrosis;Radiation Injuries;Retinal Degeneration;Schistosomiasis;Stomach Diseases;Thyroid Neoplasms;Tongue Neoplasms;Trophoblasts;Vitiligo;Prolactinoma;Precursor B-Cell Lymphoblastic Leukemia-Lymphoma;Leukemia, Biphenotypic, Acute;Leukemia-Lymphoma, Adult T-Cell;HIV-1;Endometrial Neoplasms;Adrenocortical Adenoma;Cholangiocarcinoma;Cervical Intraepithelial Neoplasia;Ischemic Preconditioning;Hepatitis B, Chronic;Hypoxia-Ischemia, Brain;Hearing Loss;ACTH-Secreting Pituitary Adenoma;Azoospermia;Small Cell Lung Carcinoma;Drug-Induced Liver Injury;Kidney Failure, Acute;

module 59

module 60 Atrophy;Dementia;Cardiomegaly;Heart Failure;Hemangioma;Hypertension;Kidney Failure, Chronic;Myocardial Infarction;Myocarditis;Myopia;Polycythemia Vera;RNA Virus Infections;Scleroderma, Systemic;Supranuclear Palsy, Progressive;Trophoblasts;Reperfusion Injury;Precursor B-Cell Lymphoblastic Leukemia-Lymphoma;Retinal Neovascularization;Nerve Sheath Neoplasms;

module 61 Breast Neoplasms;Prostatic Neoplasms;

module 62 Colonic Neoplasms;

module 63 Adenocarcinoma;Colonic Neoplasms;Crohn Disease;Fatty Liver;Kidney Failure, Chronic;Pancreatic Neoplasms;RNA Virus Infections;

module 64 Hepatitis B;

module 65 Adenoma;Aging;Albuminuria;Amyotrophic Lateral Sclerosis;Anoxia;Anus Neoplasms;Aortic Valve Stenosis;Astrocytoma;Biliary Tract Neoplasms;Brain Injury;Brain Neoplasms;Cardiomyopathy, Dilated;Cardiomyopathy, Hypertrophic;Cardiovascular Diseases;Carotid Artery Diseases;Cerebellar Neoplasms;Intracranial Aneurysm;Uterine Cervical Neoplasms;Cholesteatoma;Chondrodysplasia Punctata;Choriocarcinoma;Colitis, Ulcerative;Crohn Disease;Cryptosporidium;Demyelinating Diseases;Dermatitis, Atopic;Dyspepsia;Eclampsia;Endothelium, Vascular;Esophagus;Fibrosis;Giant Cell Tumors;Granulosa Cell Tumor;Hamartoma Syndrome, Multiple;Heart Diseases;Cardiomegaly;Hepatitis, Chronic;HIV;Huntington Disease;Hyperglycemia;Hypopharyngeal Neoplasms;Kidney Neoplasms;Leiomyosarcoma;Leprosy;Leukemia;Liver Cirrhosis;Long QT Syndrome;Lymphoma, Non-Hodgkin;Marek Disease;Metabolic Diseases;Cardiomyopathies;Myocardium;Neoplasms, Germ Cell and Embryonal;Neuroma, Acoustic;Neutropenia;Obesity;Osteolysis;Polycythemia Vera;Pulmonary Fibrosis;Radiation Injuries;RNA Virus Infections;Sarcoma;Sarcoma, Ewing's;Osteosarcoma;Sezary Syndrome;Spinal Cord Injuries;Testicular Neoplasms;Vascular Diseases;Wounds and Injuries;Inflammatory Bowel Diseases;Reperfusion Injury;Myocardial Reperfusion Injury;Precursor B-Cell Lymphoblastic Leukemia-Lymphoma;AIDS Dementia Complex;HIV Infections;Retinal Neovascularization;Lymphoma, B-Cell;Myocardial Ischemia;Aortic Aneurysm, Abdominal;Focal Epithelial Hyperplasia;Hepatoblastoma;Carcinoma, Embryonal;Carcinoma, Endometrioid;Carcinoma, Ductal, Breast;Carcinoma, Neuroendocrine;Cholangiocarcinoma;Carcinoma, Small Cell;Cystitis, Interstitial;Ischemic Preconditioning;Muscular Dystrophy, Duchenne;Hypoxia-Ischemia, Brain;Muscular Disorders, Atrophic;Pulmonary Disease, Chronic Obstructive;Myocytes, Cardiac;SARS Virus;ACTH-Secreting Pituitary Adenoma;Kidney Failure, Acute;Fatty Liver, Non-Alcoholic;

module 66

module 67 Abortion, Habitual;Arthritis, Rheumatoid;Astrocytoma;Carcinoma, Non-Small-Cell Lung;Carcinoma, Squamous Cell;Cerebral Infarction;Cerebral Ischemia;Uterine Cervical Neoplasms;Chlamydia Infections;Eczema;Erythropoiesis;Eye Abnormalities;Gastrointestinal Neoplasms;Ischemia;Creutzfeldt-Jakob Syndrome;Keratoconus;Lymphoma;Waldenstrom Macroglobulinemia;Moyamoya Disease;Myocarditis;Psychotic Disorders;Tongue Neoplasms;Vitiligo;Arthritis, Psoriatic;Retinal Neovascularization;Gerstmann-Straussler-Scheinker Disease;Prion Diseases;Cholangiocarcinoma;Cervical Intraepithelial Neoplasia;Sepsis;Neurodegenerative Diseases;Cocaine-Related Disorders;Stroke;Precursor T-Cell Lymphoblastic Leukemia-Lymphoma;

module 68

module 69

module 70 Mouth Neoplasms;

module 71

module 72 Carcinoma, Ehrlich Tumor;Fatty Liver;Helplessness, Learned;Hepatitis, Chronic;Medulloblastoma;Pain;Pheochromocytoma;Retinal Degeneration;Leukemia, Biphenotypic, Acute;Cervical Intraepithelial Neoplasia;Sepsis;Hearing Loss;

module 73 Burns;Carcinoma, Basal Cell;Ependymoma;Fatty Liver;Laryngeal Neoplasms;Leukemia;Lupus Nephritis;Neoplasms;Polycythemia Vera;Trophoblasts;Precursor B-Cell Lymphoblastic Leukemia-Lymphoma;Leukemia-Lymphoma, Adult T-Cell;Leukemia, Myelogenous, Chronic, BCR-ABL Positive;Retinal Neovascularization;Cervical Intraepithelial Neoplasia;

module 74

module 75 Neuroblastoma;Thyroid Neoplasms;Neoplasms, Squamous Cell;

module 76 Amyloidosis;Anus Neoplasms;Asthma;Atrophy;Barrett Esophagus;Biliary Tract Neoplasms;Brain Injury;Carcinoma;Cardiomyopathy, Dilated;Cataract;Cholesteatoma;Colonic Neoplasms;Crohn Disease;Digestive System Neoplasms;Endometriosis;Ependymoma;Gastrointestinal Neoplasms;Head and Neck Neoplasms;Hodgkin Disease;Hyperglycemia;Hypertrophy;Inflammation;Influenza, Human;Leiomyoma;Liposarcoma;Liver Neoplasms;Mesothelioma;Mycosis Fungoides;Myelodysplastic Syndromes;Nasopharyngeal Neoplasms;Nevus, Pigmented;Periodontitis;Pituitary Neoplasms;Retinoblastoma;Sarcoma, Ewing's;Sarcoma, Kaposi;Spinal Cord Injuries;Stomach Diseases;Sarcoma, Synovial;Leukemia, Myeloid, Acute;Endometrial Neoplasms;Lung Diseases, Interstitial;Hepatoblastoma;Adrenocortical Carcinoma;Multiple Endocrine Neoplasia Type 1;Neurodegenerative Diseases;Cocaine-Related Disorders;Stroke;ACTH-Secreting Pituitary Adenoma;Lymphoma, Primary Effusion;

module 77 Lung Neoplasms;Colorectal Neoplasms;

module 78 Abortion, Habitual;Barrett Esophagus;Esophageal Neoplasms;Hepatitis, Chronic;Keloid;Mouth Neoplasms;Moyamoya Disease;Vitiligo;Leukemia, Myeloid, Acute;Sepsis;Small Cell Lung Carcinoma;

module 79

module 80 Acquired Immunodeficiency Syndrome;Adenoma;Albuminuria;Alzheimer Disease;Amyotrophic Lateral Sclerosis;Biliary Atresia;Brain Injury;Cardiovascular Diseases;Intracranial Aneurysm;Chondrodysplasia Punctata;Cicatrix;Diabetes Mellitus;Diabetic Nephropathies;Dyspepsia;Endomyocardial Fibrosis;Endothelium, Vascular;Erythropoiesis;Fibrosis;Granulosa Cell Tumor;HIV;Hypopharyngeal Neoplasms;Inflammation;Influenza, Human;Ischemia;Kidney Diseases;Leiomyosarcoma;Liver Cirrhosis;Liver Diseases;Liver Neoplasms;Lupus Erythematosus, Systemic;Meningioma;Metabolic Diseases;Mouth Neoplasms;Multiple Myeloma;Multiple Sclerosis;Myocardial Infarction;Myocardium;Myotonic Dystrophy;Nervous System Diseases;Parkinson Disease;Pre-Eclampsia;Pulmonary Fibrosis;Radiation Injuries;Retinoblastoma;Rhabdomyosarcoma;Sarcoma;Osteosarcoma;Scleroderma, Localized;Scleroderma, Systemic;Leukemia, lymphoblastic, Chronic;Leukemia, Myeloid, Chronic-Phase;AIDS Dementia Complex;Lymphoma, B-Cell;Aortic Aneurysm, Thoracic;Carcinoma, Small Cell;Muscular Dystrophy, Duchenne;Irritable Bowel Syndrome;Atherosclerosis;Precursor Cell Lymphoblastic Leukemia-Lymphoma;Eosinophilic Esophagitis;

module 81 Adenocarcinoma;Carcinoma, Ehrlich Tumor;Intracranial Aneurysm;Endomyocardial Fibrosis;Fatty Liver;Gastrointestinal Neoplasms;Kidney Failure, Chronic;Mesothelioma;Pain;Pancreatic Neoplasms;Retinal Degeneration;RNA Virus Infections;Endometrial Neoplasms;Nerve Sheath Neoplasms;Hearing Loss;Small Cell Lung Carcinoma;

module 82 Carcinoma, Non-Small-Cell Lung;Lung Neoplasms;

module 83 Carcinoma, Basal Cell;Carcinoma, Squamous Cell;Head and Neck Neoplasms;Mouth Neoplasms;Skin Neoplasms;

module 84 Abortion, Habitual;Astrocytoma;Autistic Disorder;Biliary Tract Neoplasms;Tourette Syndrome;Glioblastoma;Graves Disease;Hemangioma;Lymphoma;Myocarditis;Periodontitis;Skin Neoplasms;Trophoblasts;Tuberculosis, Pulmonary;Prolactinoma;Inflammatory Bowel Diseases;Leukemia, Myelogenous, Chronic, BCR-ABL Positive;Leukemia, Promyelocytic, Acute;Retinal Neovascularization;Gerstmann-Straussler-Scheinker Disease;Prion Diseases;Sepsis;Neurodegenerative Diseases;Muscular Dystrophy, Duchenne;Mastocytosis, Systemic;Precursor T-Cell Lymphoblastic Leukemia-Lymphoma;Frontotemporal Lobar Degeneration;

module 85 Astrocytoma;Atrophy;Central Nervous System Diseases;Endomyocardial Fibrosis;Fragile X Syndrome;Glioblastoma;Myocarditis;Pulmonary Embolism;RNA Virus Infections;Muscular Dystrophy, Facioscapulohumeral;Mastocytosis, Systemic;

module 86

module 87 Astrocytoma;Carcinoma;Glioma;Neuroblastoma;

module 88

module 89 Abortion, Habitual;Carcinoma;Carcinoma, Ehrlich Tumor;Cerebellar Neoplasms;Choriocarcinoma;Diabetic Nephropathies;Diabetic Retinopathy;Fatty Liver;Fibrosarcoma;Giant Cell Tumors;Glomerulonephritis;Glomerulonephritis, IGA;Helplessness, Learned;Hepatitis;Hepatitis B;Hepatitis, Chronic;Hepatitis C;Hyperlipidemias;Kidney Neoplasms;Leiomyoma;Liver Neoplasms;Lupus Erythematosus, Systemic;Lupus Nephritis;Neoplasms;Neoplasms, Germ Cell and Embryonal;Nephrosclerosis;Pheochromocytoma;Retinal Degeneration;Sarcoma, Ewing's;Skin Neoplasms;Testicular Neoplasms;Thyroid Neoplasms;Trophoblasts;Precursor B-Cell Lymphoblastic Leukemia-Lymphoma;Carcinoma, Embryonal;Cholangiocarcinoma;Cervical Intraepithelial Neoplasia;Hepatitis B, Chronic;Hepatitis C, Chronic;Hearing Loss;Lipid Metabolism Disorders;Azoospermia;Drug-Induced Liver Injury;

module 90 Child Development Disorders, Pervasive;Fanconi Anemia;Inflammation;Liposarcoma;Meningioma;Nervous System Diseases;Leukemia, Myeloid, Acute;Leukemia, Promyelocytic, Acute;Neurodegenerative Diseases;Atherosclerosis;Precursor Cell Lymphoblastic Leukemia-Lymphoma;

module 91

module 92 Astrocytoma;Brain Neoplasms;Carcinoma;Carcinoma, Squamous Cell;Eye Abnormalities;Glioblastoma;Head and Neck Neoplasms;Keratoconus;Kidney Failure, Chronic;Leiomyoma;Leiomyosarcoma;Leukemia, Myeloid;Waldenstrom Macroglobulinemia;Mouth Neoplasms;RNA Virus Infections;Skin Neoplasms;Stomach Neoplasms;Thyroid Neoplasms;Tongue Neoplasms;Retinal Neovascularization;Panic Disorder;Carcinoma, Embryonal;Cervical Intraepithelial Neoplasia;Nerve Sheath Neoplasms;Muscular Dystrophy, Facioscapulohumeral;Dyslipidemias;

module 93 Abortion, Habitual;Atrophy;Coronary Artery Disease;Dementia;Cardiomegaly;Heart Failure;Hypertension;Ischemia;Kidney Failure, Chronic;Myasthenia Gravis;Myocardial Infarction;Myocarditis;RNA Virus Infections;Reperfusion Injury;Nerve Sheath Neoplasms;Muscular Dystrophy, Facioscapulohumeral;

module 94 Aging;Anoxia;Intracranial Aneurysm;Hepatitis, Chronic;Mesothelioma;Cardiomyopathies;Parkinson Disease;Sarcoma;Sezary Syndrome;Testicular Neoplasms;Reperfusion Injury;Carcinoma, Small Cell;Pulmonary Disease, Chronic Obstructive;SARS Virus;Small Cell Lung Carcinoma;

module 95 Adrenal Cortex Neoplasms;Arthritis;Diabetes Mellitus;Diabetes Mellitus, Type 2;Graves Disease;Hand, Foot and Mouth Disease;Medulloblastoma;Muscular Dystrophies;Myasthenia Gravis;Myositis Ossificans;Periodontitis;Prostatic Neoplasms;Salivary Gland Neoplasms;Sarcoma, Synovial;Hepatoblastoma;Adrenocortical Adenoma;Adrenocortical Carcinoma;Ischemic Preconditioning;Muscular Disorders, Atrophic;Kidney Failure, Acute;

module 96 Child Development Disorders, Pervasive;Glioblastoma;Lung Neoplasms;Medulloblastoma;Meningioma;Muscular Dystrophies;Neoplasms, Glandular and Epithelial;Nervous System Diseases;Pancreatic Neoplasms;Pregnancy, Ectopic;Trophoblasts;Leukemia, Myeloid, Acute;Cervical Intraepithelial Neoplasia;Mastocytosis, Systemic;

module 97 Abortion, Habitual;Adenoma;Aging;Albuminuria;Alopecia;Amyotrophic Lateral Sclerosis;Anemia, Sickle Cell;Angina, Unstable;Anoxia;Anus Neoplasms;Anxiety Disorders;Aortic Valve Stenosis;Arthritis;Arthritis, Rheumatoid;Asthma;Atrial Fibrillation;Atrophy;Barrett Esophagus;Behcet Syndrome;Biliary Atresia;Brain Injury;Brain Neoplasms;Burkitt Lymphoma;Burns;Cardiomyopathy, Dilated;Cardiovascular Diseases;Carotid Artery Diseases;Cataract;Central Nervous System Diseases;Cerebellar Neoplasms;Intracranial Aneurysm;Cerebral Hemorrhage;Cerebral Infarction;Cerebral Ischemia;Chlamydia Infections;Cholesteatoma;Chondrodysplasia Punctata;Chordoma;Choriocarcinoma;Cicatrix;Colitis;Colitis, Ulcerative;Colorectal Neoplasms, Hereditary Nonpolyposis;Coronary Artery Disease;Cryptosporidium;Cystic Fibrosis;Dementia;Demyelinating Diseases;Dermatitis, Atopic;Diabetes Mellitus;Diabetes Mellitus, Type 1;Diabetes Mellitus, Type 2;Diabetic Nephropathies;Diabetic Retinopathy;Down Syndrome;Dyspepsia;Eclampsia;Eczema;Encephalomyelitis, Autoimmune, Experimental;Endothelium, Vascular;Erythropoiesis;Esophagus;Eye Abnormalities;Fanconi Anemia;Fibroblasts;Fibrosarcoma;Fibrosis;Fragile X Syndrome;Francisella;Gastritis, Atrophic;Giant Cell Tumors;Tourette Syndrome;Glomerulonephritis;Glomerulonephritis, IGA;Graft vs Host Disease;Granulosa Cell Tumor;Graves Disease;Hamartoma Syndrome, Multiple;Hand, Foot and Mouth Disease;Heart Diseases;Hemangioma;Hepatitis;HIV;Huntington Disease;Hyperglycemia;Hyperlipidemias;Hypertension;Hypertrophy;Hypopharyngeal Neoplasms;Inflammation;Influenza, Human;Intervertebral Disk;Ischemia;Creutzfeldt-Jakob Syndrome;Keloid;Keratoconus;Kidney Diseases;Kidney Neoplasms;Leiomyosarcoma;Leprosy;Leukoplakia, Oral;Liposarcoma;Liver Cirrhosis, Biliary;Liver Diseases, Alcoholic;Liver Neoplasms;Long QT Syndrome;Lung Diseases;Lupus Erythematosus, Systemic;Lupus Nephritis;Lymphoma, Non-Hodgkin;Lymphoproliferative Disorders;Waldenstrom Macroglobulinemia;Marek Disease;Intellectual Disability;Metabolic Diseases;Moyamoya Disease;Multiple Sclerosis;Myasthenia Gravis;Mycosis Fungoides;Myeloproliferative Disorders;Cardiomyopathies;Myocarditis;Myocardium;Myopia;Myositis Ossificans;Nasal Polyps;Neoplasms, Germ Cell and Embryonal;Neoplasms, Glandular and Epithelial;Neuroma, Acoustic;Neutropenia;Nevus, Pigmented;Obesity;Oligodendroglioma;Osteoarthritis;Osteolysis;Osteoporosis;Periodontal Diseases;Polycythemia Vera;Pregnancy, Ectopic;Psoriasis;Psychotic Disorders;Pulmonary Embolism;Pulmonary Fibrosis;Radiation Injuries;RNA Virus Infections;Sarcoma;Sarcoma, Ewing's;Schistosomiasis;Scleroderma, Localized;Scleroderma, Systemic;Sezary Syndrome;Sjogren's Syndrome;Spinal Cord Injuries;Stomach Diseases;Supranuclear Palsy, Progressive;Synapses;Sarcoma, Synovial;Tuberculosis, Pulmonary;Vascular Diseases;Vitiligo;Wounds and Injuries;Inflammatory Bowel Diseases;Reperfusion Injury;Myocardial Reperfusion Injury;Precursor B-Cell Lymphoblastic Leukemia-Lymphoma;Leukemia, Biphenotypic, Acute;Leukemia, Myelogenous, Chronic, BCR-ABL Positive;HIV-1;AIDS Dementia Complex;Arthritis, Psoriatic;HIV Infections;Gerstmann-Straussler-Scheinker Disease;Lymphoma, Large B-Cell, Diffuse;Pemphigus, Benign Familial;Neurofibromatosis 2;Antiphospholipid Syndrome;Prion Diseases;Myocardial Ischemia;Aortic Aneurysm, Abdominal;Aortic Aneurysm, Thoracic;Focal Epithelial Hyperplasia;Lichen Planus, Oral;Lymphoma, Large-Cell, Anaplastic;Carcinoma, Embryonal;Carcinoma, Endometrioid;Carcinoma, Ductal, Breast;Carcinoma, Neuroendocrine;Cholangiocarcinoma;Carcinoma, Small Cell;Cervical Intraepithelial Neoplasia;Nerve Sheath Neoplasms;Multiple Endocrine Neoplasia Type 1;Ischemic Preconditioning;Neurodegenerative Diseases;Hepatitis B, Chronic;Hepatitis C, Chronic;Muscular Dystrophy, Duchenne;Stroke;Hypoxia-Ischemia, Brain;Myocytes, Cardiac;Hearing Loss;Mastocytosis, Systemic;Irritable Bowel Syndrome;SARS Virus;Diabetes Complications;ACTH-Secreting Pituitary Adenoma;Dyslipidemias;Atherosclerosis;Renal Insufficiency;Lipid Metabolism Disorders;Acute Coronary Syndrome;Precursor T-Cell Lymphoblastic Leukemia-Lymphoma;Lymphoma, Extranodal NK-T-Cell;Small Cell Lung Carcinoma;Drug-Induced Liver Injury;Kidney Failure, Acute;Vascular Calcification;Fatty Liver, Non-Alcoholic;

module 98 Lung Neoplasms;Colorectal Neoplasms;

module 99

module 100

module 101

module 102 Amyloidosis;Angina, Unstable;Anus Neoplasms;Arthritis;Arthritis, Rheumatoid;Autoimmune Diseases;Cardiovascular Diseases;Carotid Artery Diseases;Cholesteatoma;Chondrodysplasia Punctata;Choriocarcinoma;Colitis;Colitis, Ulcerative;Coronary Artery Disease;Dyspepsia;Endothelium, Vascular;Erythropoiesis;Fatty Liver, Alcoholic;Fibroblasts;Giant Cell Tumors;Hepatitis;Hepatitis C;Hodgkin Disease;Hyperglycemia;Hyperlipidemias;Hypertension;Ischemia;Kidney Neoplasms;Polycystic Kidney Diseases;Laryngeal Neoplasms;Leiomyosarcoma;Leukemia;Leukoplakia, Oral;Liver Diseases;Lymphoma;Lymphoproliferative Disorders;Marek Disease;Metabolic Diseases;Multiple Myeloma;Multiple Sclerosis;Myelodysplastic Syndromes;Odontogenic Tumors;Pituitary Neoplasms;Polycythemia Vera;Radiation Injuries;Rectal Neoplasms;Sarcoma;Sarcoma, Ewing's;Osteosarcoma;Schistosomiasis;Spinal Cord Injuries;Reperfusion Injury;Leukemia, B-Cell;Leukemia-Lymphoma, Adult T-Cell;AIDS Dementia Complex;Arthritis, Psoriatic;HIV Infections;Retinal Neovascularization;Lymphoma, B-Cell;Liver Failure;Myocardial Ischemia;Hepatoblastoma;Sepsis;Hepatitis B, Chronic;Hepatitis C, Chronic;Stroke;Lymphoma, Mantle-Cell;Hypoxia-Ischemia, Brain;ACTH-Secreting Pituitary Adenoma;Lipid Metabolism Disorders;Precursor T-Cell Lymphoblastic Leukemia-Lymphoma;Acute Lung Injury;Drug-Induced Liver Injury;Fatty Liver, Non-Alcoholic;

module 103

module 104 Asthma;Cholesteatoma;Digestive System Neoplasms;Hypertrophy;Inflammation;Liposarcoma;Mycosis Fungoides;Pituitary Neoplasms;Sarcoma, Kaposi;Sarcoma, Synovial;Lymphoma, Primary Effusion;

module 105 Abortion, Habitual;Amyotrophic Lateral Sclerosis;Anoxia;Anus Neoplasms;Aortic Valve Stenosis;Arrhythmias, Cardiac;Astrocytoma;Atrial Fibrillation;Atrophy;Urinary Bladder Neoplasms;Carcinoma, Squamous Cell;Cardiomyopathy, Hypertrophic;Cataract;Central Nervous System Diseases;Intracranial Aneurysm;Cerebral Ischemia;Chondrodysplasia Punctata;Chordoma;Coronary Artery Disease;Dyspepsia;Eclampsia;Endomyocardial Fibrosis;Endothelium, Vascular;Erythropoiesis;Fibroblasts;Francisella;Gastrointestinal Neoplasms;Glomerulonephritis;Head and Neck Neoplasms;Heart Defects, Congenital;Cardiomegaly;Hepatitis C;Hypertension;Hypertrophy;Laryngeal Neoplasms;Liver Cirrhosis;Liver Neoplasms;Long QT Syndrome;Waldenstrom Macroglobulinemia;Muscular Dystrophies;Musculoskeletal Abnormalities;Myelodysplastic Syndromes;Myocardial Infarction;Myocarditis;Myocardium;Myopia;Myotonic Dystrophy;Osteoporosis;Parkinson Disease;Retinal Degeneration;Rhabdomyosarcoma;Osteosarcoma;Synapses;Testicular Neoplasms;Thyroid Neoplasms;Tongue Neoplasms;Hypertrophy, Left Ventricular;Aortic Aneurysm, Thoracic;Adrenocortical Carcinoma;Carcinoma, Endometrioid;Muscular Dystrophy, Duchenne;Stroke;Pulmonary Disease, Chronic Obstructive;Myocytes, Cardiac;Distal Myopathies;Atherosclerosis;Acute Coronary Syndrome;

module 106

module 107

module 108 Adenocarcinoma;Breast Neoplasms;Colonic Neoplasms;Muscular Dystrophies;Myeloproliferative Disorders;Ovarian Neoplasms;Prostatic Neoplasms;Salivary Gland Neoplasms;Skin Neoplasms;Stomach Neoplasms;Panic Disorder;Lung Diseases, Interstitial;Frontotemporal Lobar Degeneration;

module 109 Amyloidosis;Aortic Valve Insufficiency;Aortic Valve Stenosis;Cataract;Cerebral Hemorrhage;Esophageal Neoplasms;Heart Defects, Congenital;Ischemia;Lymphoma;Multiple Myeloma;Pre-Eclampsia;Skin Neoplasms;Lymphoma, T-Cell;Prion Diseases;Carcinoma, Embryonal;Cholangiocarcinoma;Nerve Sheath Neoplasms;Multiple Endocrine Neoplasia Type 1;Sepsis;Ischemic Preconditioning;Neurodegenerative Diseases;Cocaine-Related Disorders;Lymphoma, Mantle-Cell;SARS Virus;Atherosclerosis;Renal Insufficiency;Kidney Failure, Acute;

module 110 Carcinoma, Squamous Cell;Head and Neck Neoplasms;Nasopharyngeal Neoplasms;Thyroid Neoplasms;

module 111 Alopecia;Carotid Artery Diseases;Cerebral Ischemia;Coronary Artery Disease;Eclampsia;Fanconi Anemia;Tourette Syndrome;HIV;Hyperglycemia;Infertility, Male;Inflammation;Ischemia;Liver Cirrhosis;Lupus Erythematosus, Systemic;Marek Disease;Metabolic Diseases;Multiple Myeloma;Myelodysplastic Syndromes;Obesity;Osteoarthritis;Polycythemia Vera;Pre-Eclampsia;Radiation Injuries;Sarcoma;Osteosarcoma;Reperfusion Injury;Leukemia, lymphoblastic, Chronic;Precursor B-Cell Lymphoblastic Leukemia-Lymphoma;Leukemia, Promyelocytic, Acute;Lymphoma, Large B-Cell, Diffuse;Ischemic Preconditioning;Cocaine-Related Disorders;Stroke;Atherosclerosis;Precursor Cell Lymphoblastic Leukemia-Lymphoma;Precursor T-Cell Lymphoblastic Leukemia-Lymphoma;Kidney Failure, Acute;

module 112 Glomerulonephritis;

module 113

module 114 Atrial Fibrillation;Melanoma;

module 115 Colonic Neoplasms;Lung Neoplasms;Pancreatic Neoplasms;Colorectal Neoplasms;

module 116 Acquired Immunodeficiency Syndrome;Biliary Atresia;Brain Injury;Cicatrix;Crohn Disease;Diabetic Nephropathies;Endomyocardial Fibrosis;Fibrosis;Hepatitis B;Hepatitis C;Influenza, Human;Ischemia;Kidney Diseases;Liver Diseases;Liver Neoplasms;Lupus Erythematosus, Systemic;Meningioma;Myocardium;Nervous System Diseases;Retinoblastoma;Rhabdomyosarcoma;Scleroderma, Localized;Scleroderma, Systemic;Leukemia, Myeloid, Chronic-Phase;Aortic Aneurysm, Thoracic;Carcinoma, Small Cell;Hepatitis B, Chronic;Muscular Dystrophy, Duchenne;Irritable Bowel Syndrome;Drug-Induced Liver Injury;Vascular Calcification;

module 117 Asthma;Cerebral Ischemia;Digestive System Neoplasms;Gastrointestinal Neoplasms;Head and Neck Neoplasms;Hypertrophy;Liposarcoma;Mycosis Fungoides;Myelodysplastic Syndromes;Nasopharyngeal Neoplasms;Pituitary Neoplasms;Sarcoma, Kaposi;Sarcoma, Synovial;Endometrial Neoplasms;Cocaine-Related Disorders;Stroke;Lymphoma, Primary Effusion;

module 118 Adenocarcinoma;Adrenal Cortex Neoplasms;Urinary Bladder Neoplasms;Carcinoma, Squamous Cell;Colonic Neoplasms;Diabetes Mellitus;Hypertension;Pregnancy, Ectopic;Prostatic Neoplasms;Scleroderma, Systemic;Carcinoma, Endometrioid;SARS Virus;Atherosclerosis;

module 119 Neoplasms;

module 120 Cerebellar Neoplasms;Uterine Cervical Neoplasms;Ependymoma;Fatty Liver;Fibrosarcoma;Heart Failure;Hepatitis, Chronic;Lymphoma;Medulloblastoma;Neoplasms, Germ Cell and Embryonal;Skin Neoplasms;Testicular Neoplasms;Precursor B-Cell Lymphoblastic Leukemia-Lymphoma;Prion Diseases;Carcinoma, Embryonal;Cholangiocarcinoma;Cervical Intraepithelial Neoplasia;Neurodegenerative Diseases;Azoospermia;

module 121 Abortion, Habitual;Adenocarcinoma;Carcinoma, Squamous Cell;Esophageal Neoplasms;Head and Neck Neoplasms;Mouth Neoplasms;Pancreatic Neoplasms;Small Cell Lung Carcinoma;

module 122 Alopecia;Alzheimer Disease;Angina, Unstable;Burkitt Lymphoma;Carcinoma, Basal Cell;Hemangiosarcoma;Hodgkin Disease;Polycystic Kidney Diseases;Laryngeal Neoplasms;Leiomyoma;Lung Diseases;Mesothelioma;Musculoskeletal Abnormalities;Neuroblastoma;Obesity;Periodontal Diseases;Osteosarcoma;Toxoplasma;Toxoplasmosis;Vascular Diseases;Inflammatory Bowel Diseases;Leukemia, B-Cell;Leukemia, Biphenotypic, Acute;HIV-1;Antiphospholipid Syndrome;Lung Diseases, Interstitial;Lymphoma, Large-Cell, Anaplastic;Hematologic Neoplasms;Lymphoma, Mantle-Cell;

module 123 Alopecia;Anus Neoplasms;Arthritis, Rheumatoid;Atrial Fibrillation;Autistic Disorder;Barrett Esophagus;Behcet Syndrome;Burns;Cardiomyopathy, Dilated;Carotid Artery Diseases;Cerebral Ischemia;Chlamydia Infections;Chondrodysplasia Punctata;Colitis;Colorectal Neoplasms, Hereditary Nonpolyposis;Cystic Fibrosis;Dementia;Demyelinating Diseases;Diabetes Mellitus;Diabetes Mellitus, Type 2;Down Syndrome;Dyspepsia;Eczema;Encephalomyelitis, Autoimmune, Experimental;Endothelium, Vascular;Erythropoiesis;Fibroblasts;Francisella;Gastritis, Atrophic;Giant Cell Tumors;Glomerulonephritis;Glomerulonephritis, IGA;Graft vs Host Disease;HIV;Hyperglycemia;Influenza, Human;Intervertebral Disk;Creutzfeldt-Jakob Syndrome;Kidney Failure, Chronic;Laryngeal Neoplasms;Leiomyosarcoma;Leukoplakia, Oral;Liver Diseases, Alcoholic;Liver Neoplasms;Lung Diseases;Lupus Erythematosus, Systemic;Lupus Nephritis;Lymphoproliferative Disorders;Marek Disease;Moyamoya Disease;Myocardial Infarction;Myocarditis;Myopia;Obesity;Osteoarthritis;Periodontal Diseases;Polycythemia Vera;Psoriasis;Psychotic Disorders;Schistosomiasis;Scleroderma, Systemic;Sjogren's Syndrome;Stomach Diseases;Supranuclear Palsy, Progressive;Myocardial Reperfusion Injury;Precursor B-Cell Lymphoblastic Leukemia-Lymphoma;Leukemia-Lymphoma, Adult T-Cell;Leukemia, Myelogenous, Chronic, BCR-ABL Positive;Arthritis, Psoriatic;Gerstmann-Straussler-Scheinker Disease;Lymphoma, Large B-Cell, Diffuse;Prion Diseases;Lichen Planus, Oral;Carcinoma, Neuroendocrine;Sepsis;Ischemic Preconditioning;Neurodegenerative Diseases;Hypoxia-Ischemia, Brain;Diabetes Complications;Renal Insufficiency;Acute Coronary Syndrome;Precursor T-Cell Lymphoblastic Leukemia-Lymphoma;Lymphoma, Extranodal NK-T-Cell;Eosinophilic Esophagitis;Kidney Failure, Acute;

module 124 Leukemia, Myeloid;Nasal Polyps;Leukemia, Myeloid, Acute;Lymphoma, T-Cell;Hematologic Neoplasms;

module 125 Amyloidosis;Anemia, Sickle Cell;Aortic Valve Insufficiency;Aortic Valve Stenosis;Cataract;Intracranial Aneurysm;Cerebral Hemorrhage;Endomyocardial Fibrosis;Esophageal Neoplasms;Glioblastoma;Heart Defects, Congenital;Hypertrophy;Lymphoma;Nasopharyngeal Neoplasms;Parkinson Disease;Pituitary Neoplasms;Leukemia, Myeloid, Acute;Lymphoma, T-Cell;Prion Diseases;Cholangiocarcinoma;Cervical Intraepithelial Neoplasia;Multiple Endocrine Neoplasia Type 1;Neurodegenerative Diseases;Lymphoma, Mantle-Cell;Vascular Calcification;

module 126 Alopecia;Angina, Unstable;Arthritis, Rheumatoid;Atrial Fibrillation;Burkitt Lymphoma;Colonic Neoplasms;Ependymoma;Hamartoma Syndrome, Multiple;Hemangiosarcoma;Hodgkin Disease;Polycystic Kidney Diseases;Leiomyosarcoma;Leukemia;Leukemia, Myeloid;Lung Diseases;Lupus Erythematosus, Systemic;Medulloblastoma;Mesothelioma;Musculoskeletal Abnormalities;Mycosis Fungoides;Myeloproliferative Disorders;Nasal Polyps;Nevus, Pigmented;Obesity;Periodontitis;Retinoblastoma;RNA Virus Infections;Scleroderma, Systemic;Toxoplasma;Toxoplasmosis;Vascular Diseases;Inflammatory Bowel Diseases;Leukemia, B-Cell;Leukemia-Lymphoma, Adult T-Cell;HIV-1;Lymphoma, B-Cell;Lymphoma, T-Cell;Pemphigus, Benign Familial;Antiphospholipid Syndrome;Lung Diseases, Interstitial;Lymphoma, Large-Cell, Anaplastic;Hematologic Neoplasms;Muscular Dystrophy, Facioscapulohumeral;Lymphoma, Mantle-Cell;SARS Virus;Azoospermia;

module 127 Alopecia;Anemia, Sickle Cell;Anxiety Disorders;Astrocytoma;Brain Neoplasms;Carcinoma, Squamous Cell;Diabetes Mellitus, Type 1;Eclampsia;Ependymoma;Glioma;Leukemia;Neoplasms;Neuroblastoma;Obesity;Ovarian Neoplasms;Periodontal Diseases;Polycythemia Vera;Skin Neoplasms;Toxoplasmosis;Trophoblasts;Wounds and Injuries;Precursor B-Cell Lymphoblastic Leukemia-Lymphoma;Lung Diseases, Interstitial;Carcinoma, Embryonal;Cervical Intraepithelial Neoplasia;Nerve Sheath Neoplasms;Dyslipidemias;

module 128 Astrocytoma;Atrophy;Central Nervous System Diseases;Diabetes Mellitus, Type 1;Endomyocardial Fibrosis;Eye Abnormalities;Fragile X Syndrome;Glioblastoma;Head and Neck Neoplasms;Keratoconus;Kidney Failure, Chronic;Myocarditis;Myopia;Neoplasms, Glandular and Epithelial;Pulmonary Embolism;RNA Virus Infections;Skin Neoplasms;Tongue Neoplasms;Panic Disorder;Carcinoma, Embryonal;Muscular Dystrophy, Facioscapulohumeral;Mastocytosis, Systemic;

module 129 Adenocarcinoma;Breast Neoplasms;Eye Abnormalities;Keratoconus;Stomach Neoplasms;Frontotemporal Lobar Degeneration;

module 130 Arthritis;Urinary Bladder Neoplasms;Burkitt Lymphoma;Carcinoma, Basal Cell;Carcinoma, Ehrlich Tumor;Carcinoma, Squamous Cell;Fanconi Anemia;Fatty Liver;Hand, Foot and Mouth Disease;Hodgkin Disease;Laryngeal Neoplasms;Leiomyoma;Lung Diseases;Lymphoma;Myelodysplastic Syndromes;Osteoarthritis;Pain;Pheochromocytoma;Retinal Degeneration;Osteosarcoma;Skin Neoplasms;Toxoplasmosis;Trophoblasts;Leukemia, Biphenotypic, Acute;Leukemia, Promyelocytic, Acute;Hearing Loss;

module 131 Breast Neoplasms;Carcinoma, Hepatocellular;Stomach Neoplasms;

module 132 Acquired Immunodeficiency Syndrome;Adenoviridae Infections;Aortic Valve Insufficiency;Aortic Valve Stenosis;Atrial Fibrillation;Biliary Atresia;Carcinoma, Non-Small-Cell Lung;Carcinoma, Squamous Cell;Intracranial Aneurysm;Chordoma;Cicatrix;Cryptosporidium;Endomyocardial Fibrosis;Heart Defects, Congenital;Hepatitis C;Ischemia;Liver Neoplasms;Lung Neoplasms;Mesothelioma;Mouth Neoplasms;Myocardium;Ovarian Neoplasms;Parkinson Disease;Periodontitis;RNA Virus Infections;Sarcoma;Schizophrenia;Scleroderma, Localized;Leukemia, Myeloid, Chronic-Phase;Hypertrophy, Left Ventricular;Aortic Aneurysm, Thoracic;Multiple Endocrine Neoplasia Type 1;Muscular Dystrophy, Facioscapulohumeral;Small Cell Lung Carcinoma;Vascular Calcification;

module 133 Lung Neoplasms;Colorectal Neoplasms;

module 134 Adrenal Cortex Neoplasms;Diabetes Mellitus, Type 2;Laryngeal Neoplasms;Liver Neoplasms;Psoriasis;Adrenocortical Adenoma;Precursor Cell Lymphoblastic Leukemia-Lymphoma;

module 135 Alzheimer Disease;Anxiety Disorders;Arthritis, Rheumatoid;Astrocytoma;Cerebral Infarction;Cerebral Ischemia;Colonic Neoplasms;Gastrointestinal Neoplasms;Glioblastoma;Ischemia;Medulloblastoma;Intellectual Disability;Myelodysplastic Syndromes;Myositis Ossificans;Neoplasms, Glandular and Epithelial;Oligodendroglioma;Pheochromocytoma;Rectal Neoplasms;Wounds and Injuries;Panic Disorder;Endometrial Neoplasms;Hepatoblastoma;Adrenocortical Adenoma;Sepsis;Cocaine-Related Disorders;

module 136 Adenocarcinoma;Adrenal Cortex Neoplasms;Anus Neoplasms;Barrett Esophagus;Urinary Bladder Neoplasms;Burns;Carcinoma;Carcinoma, Ehrlich Tumor;Cataract;Choriocarcinoma;Diabetic Nephropathies;Diabetic Retinopathy;Endometriosis;Esophagus;Fatty Liver;Gastrointestinal Neoplasms;Glomerulonephritis, IGA;Helplessness, Learned;Huntington Disease;Keloid;Kidney Neoplasms;Leiomyosarcoma;Leukoplakia, Oral;Lupus Erythematosus, Systemic;Meningioma;Mouth Neoplasms;Muscular Dystrophies;Nephrosclerosis;Pain;Pheochromocytoma;Retinal Degeneration;Schistosomiasis;Tongue Neoplasms;Trophoblasts;Vitiligo;Leukemia, Biphenotypic, Acute;Leukemia-Lymphoma, Adult T-Cell;Endometrial Neoplasms;Adrenocortical Adenoma;Cholangiocarcinoma;Ischemic Preconditioning;Hearing Loss;Small Cell Lung Carcinoma;Drug-Induced Liver Injury;Kidney Failure, Acute;

module 137 Adrenal Cortex Neoplasms;Amyloidosis;Amyotrophic Lateral Sclerosis;Anemia, Sickle Cell;Anxiety Disorders;Arthritis;Asthma;Intracranial Aneurysm;Choriocarcinoma;Crohn Disease;Diabetes Mellitus, Type 2;Eclampsia;Graves Disease;Hand, Foot and Mouth Disease;Hepatitis, Chronic;Huntington Disease;Muscular Dystrophies;Myasthenia Gravis;Cardiomyopathies;Myositis Ossificans;Myotonic Dystrophy;Obesity;Parkinson Disease;Sarcoma;Osteosarcoma;Sjogren's Syndrome;Supranuclear Palsy, Progressive;Synapses;Sarcoma, Synovial;Prolactinoma;Leukemia, lymphoblastic, Chronic;HIV Infections;Panic Disorder;Hepatoblastoma;Adrenocortical Adenoma;Adrenocortical Carcinoma;Carcinoma, Small Cell;Ischemic Preconditioning;Hypoxia-Ischemia, Brain;Muscular Disorders, Atrophic;Pulmonary Disease, Chronic Obstructive;Mastocytosis, Systemic;Atherosclerosis;Small Cell Lung Carcinoma;Kidney Failure, Acute;

module 138 Nasopharyngeal Neoplasms;

module 139 Abortion, Habitual;Arthritis, Rheumatoid;Carcinoma, Squamous Cell;Uterine Cervical Neoplasms;Eye Abnormalities;Hemangioma;Hepatitis B;HIV;Keratoconus;Leiomyoma;Liver Neoplasms;Lymphoma;Lymphoproliferative Disorders;Waldenstrom Macroglobulinemia;Myocarditis;Pheochromocytoma;Testicular Neoplasms;Tongue Neoplasms;Tuberculosis, Pulmonary;Retinal Neovascularization;Lymphoma, Large B-Cell, Diffuse;Prion Diseases;Cervical Intraepithelial Neoplasia;Sepsis;Neurodegenerative Diseases;Hepatitis B, Chronic;Muscular Dystrophy, Facioscapulohumeral;Mastocytosis, Systemic;Precursor T-Cell Lymphoblastic Leukemia-Lymphoma;

module 140 Alzheimer Disease;Arthritis, Rheumatoid;Astrocytoma;Carcinoma, Squamous Cell;Cerebral Infarction;Cerebral Ischemia;Fanconi Anemia;Gastrointestinal Neoplasms;Myelodysplastic Syndromes;Neoplasms, Glandular and Epithelial;Neuroblastoma;Retinoblastoma;Cholangiocarcinoma;Sepsis;Cocaine-Related Disorders;Stroke;

module 141 Carcinoma;Carcinoma, Ehrlich Tumor;Diabetic Retinopathy;Fatty Liver;Helplessness, Learned;Hodgkin Disease;Mesothelioma;Multiple Sclerosis;Neoplasms, Glandular and Epithelial;Nephrosclerosis;Ovarian Neoplasms;Pain;Pheochromocytoma;Pre-Eclampsia;Retinal Degeneration;Skin Neoplasms;Leukemia, Biphenotypic, Acute;Endometrial Neoplasms;Lung Diseases, Interstitial;Carcinoma, Embryonal;Nerve Sheath Neoplasms;Hearing Loss;Precursor Cell Lymphoblastic Leukemia-Lymphoma;

module 142 Biliary Tract Neoplasms;Choriocarcinoma;Eclampsia;Cardiomegaly;Leukemia;Marek Disease;Myelodysplastic Syndromes;Polycythemia Vera;Wounds and Injuries;HIV Infections;Lymphoma, B-Cell;Hepatoblastoma;Myocytes, Cardiac;

module 143 Crohn Disease;Neoplasms;Ovarian Neoplasms;

module 144 Crohn Disease;Hepatitis B;Hepatitis C;Neoplasms;Hepatitis B, Chronic;

module 145 Neoplasms;

module 146 Abortion, Habitual;Adenocarcinoma;Adrenal Cortex Neoplasms;Barrett Esophagus;Crohn Disease;Endometriosis;Esophageal Neoplasms;Keloid;Mouth Neoplasms;Precursor Cell Lymphoblastic Leukemia-Lymphoma;Small Cell Lung Carcinoma;

module 147

module 148 Nasopharyngeal Neoplasms;

module 149

module 150

module 151 Asthma;Atrophy;Autoimmune Diseases;Barrett Esophagus;Biliary Tract Neoplasms;Brain Injury;Carcinoma;Cardiomyopathy, Dilated;Cataract;Cholesteatoma;Colonic Neoplasms;Crohn Disease;Digestive System Neoplasms;Gastrointestinal Neoplasms;Hodgkin Disease;Hyperglycemia;Hypertrophy;Inflammation;Influenza, Human;Leiomyoma;Liposarcoma;Mesothelioma;Mycosis Fungoides;Myelodysplastic Syndromes;Nevus, Pigmented;Periodontitis;Pituitary Neoplasms;Retinoblastoma;Sarcoma, Ewing's;Sarcoma, Kaposi;Spinal Cord Injuries;Sarcoma, Synovial;Hepatoblastoma;Adrenocortical Carcinoma;ACTH-Secreting Pituitary Adenoma;Lymphoma, Primary Effusion;Acute Lung Injury;

module 152 Adenocarcinoma;Anxiety Disorders;Asthma;Multiple Myeloma;Muscular Dystrophies;Myositis Ossificans;Neoplasms, Glandular and Epithelial;Ovarian Neoplasms;Prostatic Neoplasms;Panic Disorder;Endometrial Neoplasms;

module 153 Atrial Fibrillation;Burns;Carcinoma, Non-Small-Cell Lung;Glioblastoma;Glomerulonephritis;Graves Disease;Hemangioma;Leukemia;Lupus Nephritis;Myopia;Nasopharyngeal Neoplasms;Pregnancy, Ectopic;Thyroid Neoplasms;Trophoblasts;Leukemia-Lymphoma, Adult T-Cell;Leukemia, Myelogenous, Chronic, BCR-ABL Positive;Leukemia, Myeloid, Acute;Neurodegenerative Diseases;

module 154 Brain Neoplasms;Uterine Cervical Neoplasms;Leukemia;Lymphoma;Waldenstrom Macroglobulinemia;Lymphoma, B-Cell;

module 155 Atrial Fibrillation;Melanoma;Ovarian Neoplasms;Panic Disorder;Lung Diseases, Interstitial;

module 156 Endometriosis;Precursor Cell Lymphoblastic Leukemia-Lymphoma;

module 157 Amyotrophic Lateral Sclerosis;Intracranial Aneurysm;Hepatitis, Chronic;Muscular Dystrophies;Myositis Ossificans;Parkinson Disease;Rhabdomyosarcoma;Stomach Neoplasms;Synapses;Prolactinoma;Leukemia, lymphoblastic, Chronic;Panic Disorder;Carcinoma, Endometrioid;Pulmonary Disease, Chronic Obstructive;Distal Myopathies;Frontotemporal Lobar Degeneration;

module 158 Amyotrophic Lateral Sclerosis;Anemia, Sickle Cell;Angina, Unstable;Anus Neoplasms;Cataract;Central Nervous System Diseases;Intracranial Aneurysm;Diabetes Mellitus, Type 1;Eye Abnormalities;Fanconi Anemia;Fragile X Syndrome;Tourette Syndrome;Glomerulonephritis;Graves Disease;Hemangioma;Hepatitis;Hyperglycemia;Keratoconus;Leiomyosarcoma;Leukoplakia, Oral;Liver Cirrhosis, Biliary;Liver Diseases, Alcoholic;Lung Diseases;Intellectual Disability;Metabolic Diseases;Myasthenia Gravis;Myopia;Myositis Ossificans;Oligodendroglioma;Pregnancy, Ectopic;Pulmonary Embolism;Schistosomiasis;Supranuclear Palsy, Progressive;Vitiligo;Pemphigus, Benign Familial;Cervical Intraepithelial Neoplasia;Nerve Sheath Neoplasms;Hepatitis C, Chronic;Hypoxia-Ischemia, Brain;Mastocytosis, Systemic;Dyslipidemias;Lipid Metabolism Disorders;Precursor T-Cell Lymphoblastic Leukemia-Lymphoma;

module 159 Adenocarcinoma;Fatty Liver;Kidney Failure, Chronic;Mesothelioma;Pancreatic Neoplasms;RNA Virus Infections;Endometrial Neoplasms;

module 160 Melanoma;

module 161 Adrenal Cortex Neoplasms;Mouth Neoplasms;Psoriasis;

module 162 Alopecia;Carotid Artery Diseases;Coronary Artery Disease;Dermatitis, Atopic;Fanconi Anemia;Tourette Syndrome;HIV;Hyperglycemia;Infertility, Male;Inflammation;Ischemia;Lupus Erythematosus, Systemic;Marek Disease;Metabolic Diseases;Multiple Myeloma;Myelodysplastic Syndromes;Nasal Polyps;Nevus, Pigmented;Obesity;Osteoarthritis;Radiation Injuries;Sarcoma;Osteosarcoma;Leukemia, lymphoblastic, Chronic;Precursor B-Cell Lymphoblastic Leukemia-Lymphoma;Leukemia, Biphenotypic, Acute;Leukemia, Myeloid, Acute;Leukemia, Promyelocytic, Acute;HIV-1;HIV Infections;Lymphoma, B-Cell;Lymphoma, T-Cell;Pemphigus, Benign Familial;Myocardial Ischemia;Lichen Planus, Oral;Carcinoma, Small Cell;Neoplasms, Squamous Cell;Cocaine-Related Disorders;Atherosclerosis;Precursor Cell Lymphoblastic Leukemia-Lymphoma;Precursor T-Cell Lymphoblastic Leukemia-Lymphoma;

module 163

module 164 Anxiety Disorders;Breast Neoplasms;Colonic Neoplasms;Hand, Foot and Mouth Disease;Liver Cirrhosis, Biliary;Lung Neoplasms;Meningioma;Multiple Sclerosis;Colorectal Neoplasms;Neurodegenerative Diseases;

module 165 Anxiety Disorders;Autistic Disorder;Brain Neoplasms;Carcinoma, Squamous Cell;Carotid Artery Diseases;Coronary Artery Disease;Glomerulonephritis;Psoriasis;Rhinitis, Allergic, Perennial;Scleroderma, Systemic;Thyroid Neoplasms;Colorectal Neoplasms;Lymphoma, Large B-Cell, Diffuse;Hepatoblastoma;Cervical Intraepithelial Neoplasia;Neoplasms, Squamous Cell;Muscular Dystrophy, Facioscapulohumeral;

module 166 Colonic Neoplasms;Lung Neoplasms;Colorectal Neoplasms;

module 167 Adrenal Cortex Neoplasms;Carcinoma, Squamous Cell;Diabetes Mellitus, Type 2;Laryngeal Neoplasms;Liver Neoplasms;Mouth Neoplasms;Psoriasis;

module 168

module 169 Myelodysplastic Syndromes;Rectal Neoplasms;Hepatoblastoma;ACTH-Secreting Pituitary Adenoma;

module 170

module 171

module 172 Breast Neoplasms;Fibrosarcoma;Neoplasms, Glandular and Epithelial;Prostatic Neoplasms;Stomach Neoplasms;Panic Disorder;

module 173 Adrenal Cortex Neoplasms;Anus Neoplasms;Arthritis;Arthritis, Rheumatoid;Autoimmune Diseases;Barrett Esophagus;Diabetes Mellitus, Type 2;Diabetic Retinopathy;Fatty Liver, Alcoholic;Tourette Syndrome;Hepatitis;Hyperlipidemias;Ischemia;Laryngeal Neoplasms;Liver Neoplasms;Lymphoma;Lymphoproliferative Disorders;Marek Disease;Myasthenia Gravis;Odontogenic Tumors;Pheochromocytoma;Sarcoma;Sezary Syndrome;Spinal Cord Injuries;Wounds and Injuries;Arthritis, Psoriatic;Liver Failure;Myocardial Ischemia;Adrenocortical Adenoma;Adrenocortical Carcinoma;Sepsis;Ischemic Preconditioning;Hepatitis B, Chronic;Hepatitis C, Chronic;ACTH-Secreting Pituitary Adenoma;Lipid Metabolism Disorders;Precursor T-Cell Lymphoblastic Leukemia-Lymphoma;Acute Lung Injury;Drug-Induced Liver Injury;Kidney Failure, Acute;Fatty Liver, Non-Alcoholic;

module 174 Colonic Neoplasms;Hepatitis B;Hepatitis C;

module 175 Anxiety Disorders;Colitis, Ulcerative;Glomerulonephritis;Gout;Graves Disease;Leukemia;Liver Cirrhosis, Biliary;Myelodysplastic Syndromes;Pregnancy, Ectopic;Rectal Neoplasms;Prolactinoma;Frontotemporal Lobar Degeneration;

module 176

module 177 Adenocarcinoma;Alopecia;Amyotrophic Lateral Sclerosis;Angina, Unstable;Anus Neoplasms;Arthritis;Arthritis, Rheumatoid;Atrial Fibrillation;Burkitt Lymphoma;Burns;Carcinoma, Basal Cell;Cataract;Uterine Cervical Neoplasms;Chondrodysplasia Punctata;Colonic Neoplasms;Coronary Artery Disease;Dermatitis, Atopic;Ependymoma;Fibroblasts;Hamartoma Syndrome, Multiple;Hemangiosarcoma;Hodgkin Disease;Polycystic Kidney Diseases;Leiomyosarcoma;Leukemia;Leukemia, Myeloid;Lung Diseases;Lupus Erythematosus, Systemic;Lymphoma;Mesothelioma;Multiple Myeloma;Musculoskeletal Abnormalities;Mycosis Fungoides;Myeloproliferative Disorders;Myocardial Infarction;Nasal Polyps;Neuroblastoma;Nevus, Pigmented;Obesity;Odontogenic Tumors;Periodontitis;Retinoblastoma;RNA Virus Infections;Osteosarcoma;Scleroderma, Systemic;Stomach Diseases;Toxoplasma;Toxoplasmosis;Vascular Diseases;Inflammatory Bowel Diseases;Leukemia, B-Cell;Leukemia, Biphenotypic, Acute;Leukemia-Lymphoma, Adult T-Cell;HIV-1;Arthritis, Psoriatic;Lymphoma, B-Cell;Lymphoma, T-Cell;Pemphigus, Benign Familial;Antiphospholipid Syndrome;Myocardial Ischemia;Lung Diseases, Interstitial;Lymphoma, Large-Cell, Anaplastic;Hematologic Neoplasms;Muscular Dystrophy, Facioscapulohumeral;Lymphoma, Mantle-Cell;SARS Virus;Azoospermia;

module 178 Astrocytoma;Atrophy;Autistic Disorder;Cardiomyopathy, Hypertrophic;Child Development Disorders, Pervasive;Crohn Disease;Cryptosporidium;Dementia;Fanconi Anemia;Tourette Syndrome;Glioma;Heart Diseases;Hepatitis B;Hepatitis C;Hodgkin Disease;Inflammation;Ischemia;Keloid;Leukemia, Myeloid;Liver Diseases, Alcoholic;Liver Neoplasms;Myocardial Infarction;Neutropenia;Pancreatic Neoplasms;Periodontitis;Retinoblastoma;Osteosarcoma;Schizophrenia;Supranuclear Palsy, Progressive;Vascular Diseases;Leukemia, lymphoblastic, Chronic;Leukemia, Promyelocytic, Acute;Neoplasms, Squamous Cell;

module 179 Alzheimer Disease;Arthritis, Rheumatoid;Astrocytoma;Cerebral Infarction;Cerebral Ischemia;Gastrointestinal Neoplasms;Neoplasms, Glandular and Epithelial;Retinoblastoma;Endometrial Neoplasms;Cholangiocarcinoma;Sepsis;Cocaine-Related Disorders;Stroke;

module 180

module 181 Cerebellar Neoplasms;Intracranial Aneurysm;Uterine Cervical Neoplasms;Fibrosarcoma;Giant Cell Tumors;Glomerulonephritis;Hepatitis, Chronic;Hyperlipidemias;Intellectual Disability;Mesothelioma;Mouth Neoplasms;Neoplasms;Neoplasms, Germ Cell and Embryonal;Neurilemmoma;Oligodendroglioma;Parkinson Disease;Rhinitis, Allergic, Perennial;Testicular Neoplasms;Thyroid Neoplasms;Trophoblasts;Neurofibromatosis 2;Cervical Intraepithelial Neoplasia;Pulmonary Disease, Chronic Obstructive;Azoospermia;Small Cell Lung Carcinoma;

module 182 Anxiety Disorders;Breast Neoplasms;Graves Disease;Carcinoma, Hepatocellular;Pregnancy, Ectopic;Frontotemporal Lobar Degeneration;

module 183 Giant Cell Tumors;RNA Virus Infections;Carcinoma, Embryonal;

module 184 Atrial Fibrillation;Mesothelioma;Myotonic Dystrophy;Endometrial Neoplasms;

module 185

module 186 Uterine Cervical Neoplasms;Ependymoma;Glioblastoma;Medulloblastoma;Pheochromocytoma;Cervical Intraepithelial Neoplasia;Neoplasms, Squamous Cell;

module 187 Urinary Bladder Neoplasms;

module 188 Lung Neoplasms;

module 189 Adenoviridae Infections;Astrocytoma;Atrophy;Autistic Disorder;Cardiomyopathy, Hypertrophic;Child Development Disorders, Pervasive;Cryptosporidium;Dementia;Fanconi Anemia;Tourette Syndrome;Heart Diseases;Hepatitis B;Inflammation;Liver Diseases, Alcoholic;Nervous System Diseases;Periodontitis;Psychotic Disorders;Schizophrenia;Supranuclear Palsy, Progressive;Vascular Diseases;Leukemia, lymphoblastic, Chronic;Leukemia, Promyelocytic, Acute;Neoplasms, Squamous Cell;Dyslipidemias;

module 190 Cerebral Hemorrhage;Crohn Disease;Fatty Liver;Infertility, Male;Leiomyoma;Liver Cirrhosis, Biliary;Lung Neoplasms;Melanoma;Leukemia, Biphenotypic, Acute;Panic Disorder;Irritable Bowel Syndrome;

module 191 Neoplasms;Ovarian Neoplasms;

module 192 Adenoma;Albuminuria;Alzheimer Disease;Anemia, Sickle Cell;Anoxia;Aortic Valve Insufficiency;Aortic Valve Stenosis;Astrocytoma;Biliary Tract Neoplasms;Urinary Bladder Neoplasms;Brain Neoplasms;Carcinoma, Squamous Cell;Cardiomyopathy, Hypertrophic;Cardiovascular Diseases;Carotid Artery Diseases;Uterine Cervical Neoplasms;Colonic Neoplasms;Cryptosporidium;Eclampsia;Esophagus;Granulosa Cell Tumor;Head and Neck Neoplasms;Heart Defects, Congenital;HIV;Hodgkin Disease;Hypopharyngeal Neoplasms;Leiomyosarcoma;Leprosy;Leukemia;Long QT Syndrome;Waldenstrom Macroglobulinemia;Intellectual Disability;Nasopharyngeal Neoplasms;Neuroblastoma;Neuroma, Acoustic;Neutropenia;Oligodendroglioma;Radiation Injuries;Schizophrenia;Thyroid Neoplasms;Tongue Neoplasms;Wounds and Injuries;AIDS Dementia Complex;HIV Infections;Lymphoma, B-Cell;Endometrial Neoplasms;Lichen Planus, Oral;Adrenocortical Adenoma;Carcinoma, Ductal, Breast;Cervical Intraepithelial Neoplasia;Myocytes, Cardiac;Dyslipidemias;

module 193

module 194 Abortion, Habitual;Arrhythmias, Cardiac;Atrophy;Chordoma;Crohn Disease;Endomyocardial Fibrosis;Graves Disease;Hepatitis B;Hepatitis C;Liver Neoplasms;Myocardium;Hepatitis B, Chronic;

module 195 Abortion, Habitual;Acquired Immunodeficiency Syndrome;Arrhythmias, Cardiac;Arthritis, Rheumatoid;Cardiomyopathy, Dilated;Carotid Artery Diseases;Central Nervous System Diseases;Cerebral Infarction;Cerebral Ischemia;Chordoma;Coronary Artery Disease;Endothelium, Vascular;Fragile X Syndrome;Gastrointestinal Neoplasms;Glomerulonephritis;Hepatitis;Hyperlipidemias;Keloid;Lupus Nephritis;Moyamoya Disease;Myocarditis;Pulmonary Embolism;Thyroid Neoplasms;Vitiligo;Hepatitis B, Chronic;Hepatitis C, Chronic;Cocaine-Related Disorders;Stroke;Pulmonary Disease, Chronic Obstructive;Lipid Metabolism Disorders;Drug-Induced Liver Injury;

module 196 Uterine Cervical Neoplasms;Hemangioma;Hepatitis, Chronic;Keloid;Vitiligo;Reperfusion Injury;Leukemia, Myeloid, Acute;Retinal Neovascularization;

module 197 Anus Neoplasms;Anxiety Disorders;Arthritis;Arthritis, Rheumatoid;Autistic Disorder;Brain Neoplasms;Burns;Central Nervous System Diseases;Child Development Disorders, Pervasive;Dementia;Eye Abnormalities;Fanconi Anemia;Fragile X Syndrome;Graves Disease;Carcinoma, Hepatocellular;Keratoconus;Leiomyoma;Liver Diseases, Alcoholic;Lung Diseases;Lung Neoplasms;Lymphoma;Waldenstrom Macroglobulinemia;Intellectual Disability;Neurilemmoma;Psychotic Disorders;Pulmonary Embolism;Osteosarcoma;Schizophrenia;Scleroderma, Systemic;Supranuclear Palsy, Progressive;Tongue Neoplasms;Toxoplasmosis;Prolactinoma;Lymphoma, Large B-Cell, Diffuse;Neurofibromatosis 2;Neoplasms, Squamous Cell;Muscular Dystrophy, Facioscapulohumeral;Mastocytosis, Systemic;

module 198 Urinary Bladder Neoplasms;Carcinoma, Basal Cell;Carcinoma, Renal Cell;Prostatic Neoplasms;

module 199 Autistic Disorder;Lymphoma;Melanoma;Synapses;Prolactinoma;Neurodegenerative Diseases;Mastocytosis, Systemic;Frontotemporal Lobar Degeneration;

module 200
