# Supplementary material for: Predicting miRNA-Disease Association Based on Modularity Preserving Heterogeneous Network Embedding
Source: Front Cell Dev Biol. 2021 Jun 10;9:603758. doi: 10.3389/fcell.2021.603758 (PMC8223753; doi:10.3389/fcell.2021.603758)
Supplement: Supplementary file 5 [file Table_5.DOCX]

18 0.392545

16 0.496206

15 0.324342

44 0.397388

74 0.469594

522 0.214774

26 0.333336

23 0.455312

59 0.283175

26 0.486696

23 0.426571

38 0.401408

28 0.590310

25 0.407826

19 0.329538

30 0.327338

20 0.540857

26 0.331451

2 0.679903

40 0.446047

32 0.415871

10 0.475753

35 0.428323

45 0.444704

52 0.390452

18 0.337341

42 0.226556

13 0.469441

37 0.387329

4 0.860202

3 0.679006

22 0.461699

24 0.375792

131 0.363659

3 0.572923

27 0.576500

31 0.419518

8 0.454919

180 0.185079

13 0.777145

21 0.437741

30 0.391611

18 0.406498

36 0.427621

2 0.320068

44 0.374267

86 0.394739

41 0.466458

60 0.445476

12 0.801692

2 0.683596

24 0.415542

26 0.467047

2 0.679903

102 0.397281

26 0.405792

11 0.498068

14 0.544526

26 0.452728

2 0.395135

24 0.359199

62 0.337046

79 0.392001

22 0.407002

43 0.354785

2 0.964029

8 0.595520

25 0.441361

7 0.507565

121 0.378646

23 0.572293

505 0.212078

12 0.509691

23 0.663886

87 0.227593

8 0.433552

153 0.204235

27 0.420641

21 0.468655

14 0.416395

24 0.339718

11 0.442137

10 0.618298

27 0.412740

21 0.477508

60 0.384368

38 0.332909

25 0.389266

42 0.345057

119 0.220800

14 0.381410

16 0.436541

103 0.384915

24 0.466639

3 0.470543

28 0.301398

20 0.543514

248 0.112746

2 0.475537

21 0.401634

17 0.382547

5 0.586867

11 0.496360

36 0.425616

8 0.707643

19 0.425958

16 0.360999

36 0.358198

23 0.419320

33 0.434833

8 0.355684

9 0.253019

27 0.392388

21 0.338718

32 0.338975

15 0.597428

5 0.523402

27 0.543845

28 0.359023

124 0.295256

26 0.535149

8 0.387543

15 0.687566

9 0.334412

29 0.434948

9 0.485663

18 0.689357

40.354331 0.440923
