# Supplementary material for: Predicting miRNA-Disease Association Based on Modularity Preserving Heterogeneous Network Embedding
Source: Front Cell Dev Biol. 2021 Jun 10;9:603758. doi: 10.3389/fcell.2021.603758 (PMC8223753; doi:10.3389/fcell.2021.603758)
Supplement: Supplementary file 6 [file Table_6.DOCX]

25 0.022806

88 0.024751

2 0.360098

14 0.071828

22 0.043914

16 0.031697

2 0.140941

4 0.358525

35 0.047113

3 0.406913

69 0.034452

14 0.021645

11 0.082537

15 0.038927

5 0.207440

35 0.028690

4 0.358525

18 0.052628

31 0.026967

7 0.060774

14 0.087492

22 0.028378

31 0.047949

17 0.090189

2 0.449187

23 0.019446

82 0.037162

16 0.062580

13 0.043668

51 0.047213

15 0.022725

2 0.244042

24 0.035756

2 0.234160

7 0.018679

17 0.107438

38 0.044503

3 0.121552

41 0.024904

76 0.031332

19 0.046433

2 0.422456

7 0.166747

98 0.031605

34 0.051233

12 0.033668

15 0.014240

3 0.115236

50 0.038613

2 0.360098

11 0.087960

60 0.046728

16 0.053204

2 0.408847

5 0.272817

27 0.027798

11 0.030271

4 0.127414

43 0.029660

11 0.083780

26 0.055635

16 0.086360

15 0.078514

20 0.021512

14 0.052157

218 0.027198

2 0.360098

74 0.041722

11 0.017633

67 0.041665

13 0.109094

27 0.056639

4 0.194357

37 0.067504

2 0.020250

4 0.358525

31 0.047774

17 0.040355

13 0.096455

19 0.050406

8 0.217314

29 0.031566

85 0.031543

5 0.043703

25 0.045242

46 0.032945

27 0.040601

22 0.037544

6 0.104983

26 0.045281

3 0.426836

35 0.044792

2 0.360098

7 0.041987

25 0.069434

45 0.030457

45 0.030500

29 0.034791

16 0.092483

23 0.031362

13 0.025699

3 0.047208

5 0.119483

11 0.089494

37 0.034347

11 0.070174

18 0.047537

6 0.110640

5 0.055816

2 0.095525

16 0.021699

43 0.019951

7 0.150892

3 0.041678

41 0.054170

10 0.108973

17 0.077719

3 0.383441

7 0.115596

4 0.121173

6 0.087656

41 0.039418

3 0.180006

12 0.035297

67 0.035393

33 0.061732

13 0.095821

25 0.033096

6 0.043226

3 0.000000

4 0.062767

7 0.066499

24 0.036900

11 0.056185

2 0.000000

52 0.040347

12 0.035540

31 0.051208

8 0.068120

38 0.030690

4 0.172667

8 0.024242

22.246479 0.093899
