# Supplementary material for: Predicting miRNA-Disease Association Based on Modularity Preserving Heterogeneous Network Embedding
Source: Front Cell Dev Biol. 2021 Jun 10;9:603758. doi: 10.3389/fcell.2021.603758 (PMC8223753; doi:10.3389/fcell.2021.603758)
Supplement: Supplementary file 7 [file Table_7.DOCX]

18 0.888889

16 0.9375

15 0.666667

44 0.863636

74 0.77027

522 0.461686

26 0.576923

23 0.826087

59 0.508475

26 0.807692

23 0.782609

38 0.578947

28 0.964286

25 1

19 0.842105

30 0.733333

20 0.85

26 0.653846

2 1

40 0.7

32 0.75

10 1

35 0.714286

45 0.888889

52 0.846154

18 0.777778

42 0.666667

13 0.769231

37 0.648649

4 1

3 1

22 0.772727

24 0.708333

131 0.89313

3 1

27 0.851852

31 0.612903

8 0.875

180 0.516667

13 1

21 0.761905

30 0.633333

18 0.944444

36 0.888889

2 1

44 0.863636

86 0.790698

41 0.829268

60 0.7

12 1

2 1

24 0.833333

26 0.807692

2 1

102 0.921569

26 0.692308

11 1

14 0.928571

26 0.769231

2 1

24 0.958333

62 0.903226

79 0.721519

22 0.727273

43 0.744186

2 1

8 0.75

25 0.76

7 0.857143

121 0.958678

23 0.826087

505 0.449505

12 0.916667

23 0.956522

87 0.724138

8 1

153 0.555556

27 0.814815

21 0.857143

14 1

24 0.708333

11 1

10 1

27 0.925926

21 0.809524

60 0.883333

38 0.578947

25 0.8

42 0.857143

119 0.647059

14 0.642857

16 1

103 0.92233

24 0.791667

3 1

28 0.642857

20 0.9

248 0.177419

2 1

21 0.857143

17 0.882353

5 1

11 0.909091

36 1

8 1

19 0.631579

16 0.9375

36 0.666667

23 0.695652

33 0.757576

8 1

9 0.666667

27 0.962963

21 0.666667

32 0.65625

15 0.866667

5 1

27 0.851852

28 0.607143

124 0.669355

26 0.807692

8 1

15 1

9 0.888889

29 0.793103

9 0.777778

18 1

40.3543 0.821975
