# Supplementary material for: Predicting miRNA-Disease Association Based on Modularity Preserving Heterogeneous Network Embedding
Source: Front Cell Dev Biol. 2021 Jun 10;9:603758. doi: 10.3389/fcell.2021.603758 (PMC8223753; doi:10.3389/fcell.2021.603758)
Supplement: Supplementary file 8 [file Table_8.DOCX]

25 0.52

88 0.431818

2 1

14 0.5

22 0.409091

16 0.625

2 1

4 1

35 0.371429

3 1

69 0.782609

14 0.285714

11 0.545455

15 0.4

5 0.8

35 0.428571

4 1

18 0.444444

31 0.290323

7 0.714286

14 0.785714

22 0.363636

31 0.419355

17 0.470588

2 1

23 0.26087

82 0.317073

16 0.5

13 0.538462

51 0.313725

15 0.4

2 1

24 0.375

2 1

7 0.571429

17 0.470588

38 0.447368

3 1

41 0.317073

76 0.328947

19 0.368421

2 1

7 0.571429

98 0.622449

34 0.5

12 0.75

15 0.466667

3 1

50 0.54

2 1

11 0.727273

60 0.55

16 0.5

2 1

5 0.8

27 0.296296

11 0.363636

4 1

43 0.27907

11 0.454545

26 0.346154

16 0.375

15 0.6

20 0.4

14 0.428571

218 0.330275

2 1

74 0.351351

11 0.818182

67 0.447761

13 0.538462

27 0.444444

4 1

37 0.459459

2 1

4 1

31 0.677419

17 0.705882

13 0.769231

19 0.473684

8 0.75

29 0.62069

85 0.4

5 1

25 0.68

46 0.608696

27 0.444444

22 0.181818

6 0.5

26 0.346154

3 1

35 0.457143

2 1

7 0.571429

25 0.48

45 0.4

45 0.244444

29 0.310345

16 0.875

23 0.565217

13 0.538462

3 1

5 0.8

11 0.636364

37 0.594595

11 0.818182

18 0.444444

6 0.833333

5 0.8

2 1

16 0.625

43 0.069767

7 0.714286

3 1

41 0.512195

10 0.6

17 0.588235

3 1

7 0.857143

4 1

6 0.666667

41 0.317073

3 1

12 0.333333

67 0.507463

33 0.545455

13 0.923077

25 0.44

6 0.5

3 0.333333

4 1

7 0.714286

24 0.458333

11 0.363636

2 1

52 0.634615

12 0.5

31 0.290323

8 0.5

38 0.236842

4 1

8 0.5

22.2464 0.612789
